# Extending the Projection-Based Embedding Technique to Open-Shell Systems Using the Huzinaga Equation

**DOI:** 10.1021/acs.jctc.5c00687

**Published:** 2025-07-17

**Authors:** Bence Hégely, Mihály Kállay

**Affiliations:** † Department of Physical Chemistry and Materials Science, Faculty of Chemical Technology and Biotechnology, Budapest University of Technology and Economics, Műegyetem rkp. 3., H-1111 Budapest, Hungary; ‡ HUN-REN−BME Quantum Chemistry Research Group, Műegyetem rkp. 3., H-1111 Budapest, Hungary; ¶ MTA−BME Lendület Quantum Chemistry Research Group, Műegyetem rkp. 3., H-1111 Budapest, Hungary

## Abstract

In this work, we present an approach for the embedding of wave function theory (WFT) and density functional theory (DFT) methods in a lower-level density functional approximation using the projection-based embedding (PbE) technique for open-shell systems. Our method is based on the Huzinaga equation, which is implemented in both spin-restricted and spin-unrestricted forms. While the unrestricted PbE approach has been previously reported in the literature and follows naturally from the theory for closed-shell systems, the restricted formulation required the development of a new theory, building on earlier work by Roothaan (*Rev. Mod. Phys.*, **1960**, *32*, 179) as well as Shaik and Filatov (*Chem. Phys. Lett.*, **1999**, *304*, 429). Our implementation allows for the use of arbitrary combinations of restricted and unrestricted wave functions for the high- and low-level methods, which can be advantageous for the full-system low-level calculations. The various spin-restricted and unrestricted wave function-based PbE schemes are thoroughly tested, examining how the error in reaction energies depends on the size of the subsystem treated at the high level. Additionally, we compared the performance of PbE to that of other focused multilevel approaches, such as vacuum embedding, “our-own n-layered integrated molecular orbital and molecular mechanics” (ONIOM), and multilevel local correlation (MLC). The results showed that MLC performed the best among the tested methods, while only those PbE and ONIOM variants were proved to be competitive whose low-level methods employed at most a generalized gradient approximation (GGA). It is not straightforward to determine whether PbE or ONIOM is generally more advantageous: the latter can sometimes be more accurate and computationally cheaper, while PbE offers greater robustness and the possibility of systematic improvement.

## Graphical abstract

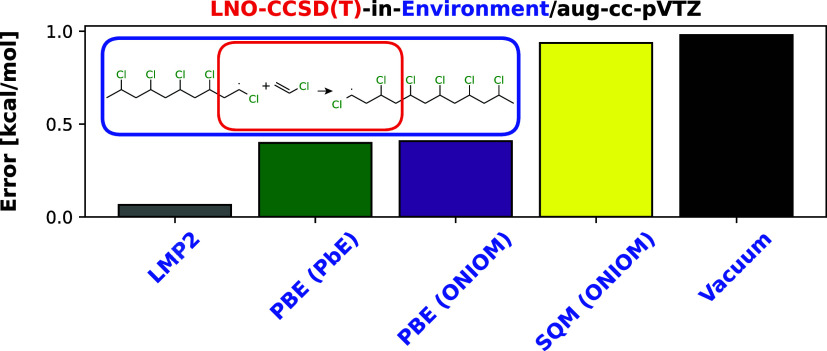

## 1 Introduction

Efficient and accurate modeling of matter is desirable in many fields of science, but the computational requirements of accurate methods increase sharply with the size of the system. In terms of accuracy, the gold standard is the coupled-cluster method with singles, doubles, and perturbative treatment of triple excitations [CCSD­(T)],^1,2^ the costs of which increase by the seventh power of the system size. The expenses of density functional theory (DFT)^3^ calculations using a hybrid-type functional, which often turns out to be the method with the best accuracy/cost ratio, increase by the fourth power of the system size, while DFT using pure functionals still exhibits cubic scaling. Even these DFT approaches or the less accurate wave function theory (WFT) methods, for instance, second-order Mo̷ller-Plesset perturbation theory (MP2), have too steep scaling that prevents their application to molecular dynamics simulations, but more simple tasks, for example, geometry optimizations, can be computationally challenging for large systems with several hundreds of atoms. Therefore, the development of efficient algorithms that reduce costs with limited loss of accuracy is desirable.

One of the established strategies for cost reduction follows the divide-and-conquer principle, which separates the system into smaller subsystems, and the costly calculations are performed only on these subsystems. This strategy is used by fragmentation-based local correlation methods,^4–8^ where the “near-sightedness of electrons” are exploited by utilizing a series of approximations based on the localized Hartree–Fock (HF) molecular orbitals (MOs). These techniques require carefully tuned semiempirical parameters to construct representative but reduced spaces where the computationally demanding operations are carried out. However, the application of these approaches is often hindered by the determination of the reference HF wave function in the case of large systems. The subsystem DFT (sDFT) procedure offers the possibility of reduced-cost calculations at the mean-field level.^9^ In this approach, the system is separated a priori into subsystems atom-wise, and in each subsystem, the Kohn–Sham (KS) equations of the embedded region are solved in the constrained electron density of the embedding subsystems. By dynamically switching the roles of the embedded and embedding subsystems in a “freeze-and-thaw” fashion, self-consistency can be achieved, and a fully relaxed electron density can be obtained without solving the KS equations for the full system.^10^ However, the resulting KS orbitals overlap, thus approximations have to be made to the nonadditive kinetic energy. The treatment of the nonadditive kinetic energy becomes cumbersome for practical applications where the subsystems are covalently bound as the approximate functionals lack the required accuracy. The fragment MO scheme of Fedorov^11^ can also be considered in the category of divide-and-conquer strategies, but the basic idea of this approach lies in the expansion of the energy using many-body terms instead of exploiting the spatial-locality of interactions.

Another common strategy for reducing costs is the application of focused models, where the total system, which is also referenced as supersystem, is divided into a chemically interesting (active) part and its environment at the atomic level. Then, the costly calculations are performed only on the active subsystem, while an approximate, more efficient technique is used for the environment. Local correlation methods can naturally adopt this principle by treating the localized MOs assigned to the subsystem with lower scaling, highly efficient methods.^12–14^ Cluster model calculations (or vacuum embedding), polarizable continuum models (PCM),^15,16^ and quantum mechanics/molecular mechanics (QM/MM)^17,18^ approaches are further examples of focused models where the environment is modeled as vacuum, continuum, or point-like atoms, respectively. A generalized version of the QM/MM approach is “Our own N-layered Integrated molecular Orbital and Molecular mechanics” (ONIOM)^19^ technique, where the energy of the total system is calculated at the low level, which can be either a QM or an MM method, and the difference of high- and low-level energies are taken for the active subsystem. Vacuum embedding, QM/MM, and the ONIOM techniques are all characterized by simple gradient theory and satisfactory accuracy if the subsystems are connected by noncovalent bonds. However, strongly interacting subsystems require link atoms to close the dangling bonds, which can lead to unwanted artifacts. Frozen-density embedding (FDE),^20^ which can be considered as a variant of sDFT, bypasses the problem of link atoms by solving the equations of the high-level method with a predetermined density of the environment, although the treatment of nonadditive kinetic energy remains an issue in the case of strongly interacting subsystems. The multilevel DFT/HF approach of Koch and Ho̷yvik^21–23^ also employs a predetermined fixed environment density, but the initial density matrix of the total system is first reconstructed to be *n*-representable, and the equations of the high-level method are solved in a reduced MO basis.

Another solution to the nonadditive kinetic energy problem of the FDE approach is offered by the projector-based embedding (PbE) method of Manby and Miller.^24,25^ In this technique, a low level method is applied to the supersystem, the MOs are localized and sorted into the active and environment subsystems based on certain criteria such as the Mulliken population of MOs on the predefined set of (active) atoms. Then, the energy of the active subsystem is determined using the low-level method, and finally, the orbitals of the active subsystem are reoptimized using a high-level method in the effective, fixed potential of the environment while keeping the orbitals of the different subsystems mutually orthogonal by the projection involving the environment density. This procedure is relatively simple, and it can be successfully applied to systems where the subsystems are covalently bound without the insertion of artificial link atoms or the approximate treatment of the nonadditive kinetic energy. For readers interested in a more detailed discussion of the origins of the PbE and its relations to sDFT, we recommend ref 26.

PbE has undergone significant development since its initial publication: Bennie et al.^27,28^ truncated the atomic orbital (AO) basis set to accelerate the construction of the high-level Fock matrix. Claudino and Mayhall^29,30^ presented a localization technique of occupied orbitals, called SPADE, that can assign orbitals to the subsystems without empirical parameters, and also developed a concentric localization method to reduce the virtual space, which is crucial for efficient correlation calculations. Lee and co-workers developed the gradient theory for PbE,^31^ thus molecular structure determination became available. Even-handed orbital selection^32^ and direct orbital selection^33–35^ schemes also represented significant innovations because these made it possible to avoid working with different numbers of active orbitals at different points of the potential energy surface (PES). Our research group also successfully contributed to the developments: by introducing the Huzinga operator,^13^ the method turned to a strictly exact embedding, which was also found to be more advantageous when the AOs are truncated.^36^ The dual basis set approach, which is similar to the method of Head-Gordon,^37^ was developed for PbE,^38^ which allowed more efficient calculations on all energy terms. The gradient theory of the Huzinaga operator-based PbE was presented recently by Csóka et al.,^39^ who showed that using DFT with a hybrid functional as a high-level method can be rather competitive with double-hybrid DFT approaches in structure determination tasks. Recently, it was also shown that projected atomic orbitals (PAO) are more preferable for excited state calculations as PAOs retain Rydberg states.^40^

The PbE method is a particularly popular approach for systems where an accurate method is essential to describe the active subsystem, such as catalytic systems containing transition metals or systems with open-shell electronic structures. In their comprehensive benchmark study of closed-shell molecules containing transition metals, Neugebauer and co-workers^41^ compared the performance of PbE and focused multilevel local correlation methods using CCSD­(T) as the reference method. They showed that the CCSD­(T)-in-MP2 approach is more accurate than CCSD­(T)-in-DFT techniques, moreover, a significant improvement in the accuracy of PbE is only achievable when double-hybrid DFT functionals are employed as the low-level theory. The first application of PbE to open-shell systems is reported in the paper of Miller and co-workers,^42^ where CCSD­(T) is utilized with a restricted open-shell reference to calculate the barrier heights of a hydrogen formation reaction employing a Co-based catalytic molecule. Using a technique similar to PbE, where the nonadditive kinetic potential is reconstructed in an exact manner, Goodpaster et al.^43^ scanned the PES of the ethylene-propylene dimer and established that WFT-in-DFT gives an accurate approximation. On the example of hexaaquairon­(II), they also showed that WFT-in-DFT significantly reduces the uncertainty of different DFT functionals regarding the energy difference of low- and high-spin states. Both unrestricted (UHF/UKS) and restricted open-shell (ROHF/ROKS) HF/KS formalisms were used for their calculations, however, the restricted calculations were only applied using the high-level method with the embedding potential of the unrestricted calculations. In a later paper,^44^ Chulhai and Goodpaster investigated the accuracy of the absolutely localized Huzinaga embedding technique^45^ using the UKS and ROKS formalisms. This approach can be considered as a variant of the PbE method, where the AOs of the subsystems are completely truncated for the respective subsystem. The reaction energy of a small thiol–ene system and the binding of a hydrogen molecule to the Fe-MOF-74 cluster were examined, and it was found that the error of the method decreases rapidly with increasing the size of the active subsystem. Using a spin-restricted formalism on metal clusters and organocatalytic molecules, Welborn and co-workers^32^ showed that the error of the PbE method in either DFT-in-DFT or WFT-in-DFT form decreases relatively rapidly with the size of the active subsystem. Similar conclusions were also drawn in the paper of Kolodzienski and Stein,^46^ where Au-clusters were studied.

In this article, the theory and implementation of the PbE technique based on the Huzinaga equation^47^ using UKS/UHF and ROKS/ROHF formalisms are presented. The motivation of this paper is 2-fold. First, theoretical and algorithmic developments are reported by deriving and implementing the spin-unrestricted and spin-restricted equations. While obtaining the spin-unrestricted equations is rather straightforward, this is not the case for the spin-restricted approach. For the latter, we utilize the ROHF method of Roothan,^48^ which was later extended to DFT by Filatov and Shaik.^49,50^ Contrary to the examples of the literature where the spin-constrained UKS method^51,52^ was utilized when a spin-restricted approach was required, the restricted open-shell (RO) scheme of the present paper is completely free of spin-contamination and its Fock matrix construction is more efficient, particularly when the method incorporates exact exchange terms. Second, the UKS and ROKS variants of the Huzinaga equation-based PbE are benchmarked on open-shell systems, and the results are also compared with other multilevel approaches. Generally, in the studies discussed above, the focus is mainly on the comparison of the PbE results with those obtained with the actual high-level method. However, the performance of PbE in comparison with other multilevel approaches is also interesting. In ref 53, where excitation energies of multilevel approaches were benchmarked, it was found that more approximate methods can be competitive with the PbE and FDE techniques. A similar conclusion was drawn in ref 54, where the authors compared the performance of ONIOM and FDE in the context of an enzymatic reaction. Thus, it is worth carrying out a similar investigation on open-shell systems, where it is well-known that the unpaired electrons tend to delocalize over large spatial moieties.

## 2 Theory

### 2.1 Notations

In the following, the usual notation of projection-based embedding theories will be used, that is, the system, which is also termed as supersystem (AB), is partitioned into an active (A) and an environment (B) subsystem at the MO (ϕ) level. The subsystems are described at different levels of theory (Λ): a high-level method (Λ = 1) is used on the active subsystem and a low-level technique is utilized on the environment (Λ = 2), moreover, on the interaction of the subsystems. The MOs, which are occupied by the electrons with α or β spins, are linear combinations of AOs (χ):

$$\phi_{p}^{\sigma }[r_{1}]=\sum_{\mu }(C^{\sigma }{)}_{\mu p}\chi_{\mu }[r_{1}]=\sum_{\mu }(C^{\sigma }{)}_{\mu p}|\chi_{\mu }\rangle$$ 1

where **r** denotes the spatial coordinates (*x*, *y*, *z*) of a particle. Throughout the article, σ is an element of the {α, β} set labeling the spin of the particles, and squared brackets emphasize a dependency of a quantity. Indices 1 and 2 of **r** denote electrons, while caligraphic letters will be used for the nuclei. **C** ^σ^ is the matrix of the MO coefficients, and Dirac’s bra-ket notation is introduced. The overlap integrals of the AOs are collected in matrix **S** with elements *S*_μν_ = ⟨χ_μ_|χ_ν_⟩. Indices *p*, *q* symbolize general MOs; *i*, *j* are used for occupied MOs; and *s*, *t* are reserved for virtuals. The MOs can be used to define the spin-dependent density matrices for the various subsystems:

$$D_{\mu \nu }^{S,\sigma }=\underset{i}{\sum }n_{i}^{\sigma }C_{\mu i}^{S,\sigma }C_{\nu i}^{S,\sigma }=\underset{i}{\sum }n_{i}^{\sigma }(R_{i}^{S,\sigma }{)}_{\mu \nu },S\in \{A,\;B,\;\mathrm{AB}\}$$ 2

where *n*_*i*_^σ^ stands for the occupation number of the *i*th orbital, which is 0 for a virtual and 1 for an occupied MO. Here, the projector of a given subspace, **R**_*i*_^*S*,σ^, is also introduced. The above equation can be recast using a simplified notation as

$$\begin{array}{cc} D^{S,\sigma }=\sum_{i}n_{i}^{\sigma }C_{i}^{S,\sigma }(C_{i}^{S,\sigma }{)}^{T} & S\in \{A,\;B,\;\mathrm{AB}\} \end{array}$$ 3

where **C**_*i*_^*S*, σ^ denotes the *i*th column of the matrix **C** ^*S*,σ^. Based on the above definitions, the spin-dependent electron densities can be defined as

$$\begin{array}{cc} \rho^{S,\sigma }[r_{1}]=\sum_{\mu \nu }D_{\mu \nu }^{S,\sigma }\chi_{\mu }[r_{1}]\chi_{\nu }[r_{1}] & S\in \{A,\;B,\;\mathrm{AB}\} \end{array}$$ 4

Furthermore, the sums of the spin-densities give the total densities

$$\begin{array}{cc} \rho^{S}[r_{1}]=\rho^{S,\alpha }[r_{1}]+\rho^{S,\beta }[r_{1}] & S\in \{A,\;B,\;\mathrm{AB}\} \end{array}$$ 5

and the supersystem density is the sum of the subsystem densities:

$$\rho^{\mathrm{AB}}\left[r_{1}\right]=\rho^{A}\left[r_{1}\right]+\rho^{B}\left[r_{1}\right]$$ 6

Note that the subsystems have integer number of electrons:

$$\begin{array}{cc} n^{S,\sigma }=\int \rho^{S,\sigma }[r_{1}]dr_{1} & S\in \{A,\;B,\;\mathrm{AB}\} \end{array}$$ 7

The usual notation is used for the HF-related operators:

$$\left(h_{\mathrm{core}}{)}_{\mu \nu }=\int \chi_{\mu }[r_{1}]\left(-\frac{1}{2}\nabla^{2}-\sum_{A}\frac{Z_{A}}{\left|r_{1}-r_{A}\right|}\right)\chi_{\nu }[r_{1}]dr_{1}=\langle \chi_{\mu }|-\frac{1}{2}\nabla^{2}-\sum_{A}\frac{Z_{A}}{\left|r_{1}-r_{A}\right|}|\chi_{\nu }\right\rangle$$ 8

$$J_{\mu \nu }[D]=\sum_{\tau \kappa }D_{\tau \kappa }\langle \chi_{\mu }[r_{1}]\chi_{\tau }[r_{2}]|\frac{1}{\left|r_{1}-r_{2}\right|}|\chi_{\nu }[r_{1}]\chi_{\kappa }[r_{2}]\rangle =\sum_{\tau \kappa }D_{\tau \kappa }\langle \chi_{\mu }\chi_{\tau }|\chi_{\nu }\chi_{\kappa }\rangle$$ 9

$$K_{\mu \nu }[D]=\sum_{\tau \kappa }D_{\tau \kappa }\langle \chi_{\mu }[r_{1}]\chi_{\tau }[r_{2}]|\frac{1}{\left|r_{1}-r_{2}\right|}|\chi_{\kappa }[r_{1}]\chi_{\nu }[r_{2}]\rangle =\sum_{\tau \kappa }D_{\tau \kappa }\langle \chi_{\mu }\chi_{\tau }|\chi_{\kappa }\chi_{\nu }\rangle$$ 10

where **h**_core_, **J**, and **K** are the core-Hamiltonian, the Coulomb operator, and the exact-exchange operator, respectively, $Z_{A}$ denotes the charge of the atomic nuclei A , and $\nabla^{2}=\left(\frac{\partial^{2}}{\partial x^{2}},\;\frac{\partial^{2}}{\partial y^{2}},\;\frac{\partial^{2}}{\partial z^{2}}\right)$ is the del operator. The DFT-related exchange–correlation energy will be denoted by *E*_xc,Λ_[**D** ^α^, **D** ^β^], and the derivative of this functional is the exchange–correlation potential:

$$\begin{array}{cc} V_{\mathrm{xc},\Lambda }^{\sigma }[D^{\alpha },\;D^{\beta }]=\frac{\partial E_{\mathrm{xc},\Lambda }[D^{\alpha },\;D^{\beta }]}{\partial D^{\sigma }} & \Lambda \in \{1,\;2\} \end{array}$$ 11

The mixing of the HF and DFT exchange terms will be expressed by the *a*_HF,Λ_ parameter, furthermore, the Tr symbol will be used to denote the trace of matrices. Finally, the repulsion energy of the nuclei is evaluated by equation

$$V_{\mathrm{nn}}=\underset{A<B}{\sum }\frac{Z_{A}Z_{B}}{\left|r_{A}-r_{B}\right|}$$ 12

### 2.2 Unrestricted Open-Shell Huzinaga Embedding

The energy of the PbE method in the spin-unrestricted Ansatz can be written as the Taylor series of the multilevel energy, *E*_12,UKS_, truncated at first-order at the density of subsystem A as

$$E_{\mathrm{PbE},\mathrm{UKS}}=E_{12,\mathrm{UKS}}+\underset{\sigma }{\sum }\mathrm{Tr}\left\{\left({\overset{\sim }{D}}^{\sigma ,A}-D^{\sigma ,A}\right)\frac{\partial E_{12,\mathrm{UKS}}}{\partial D^{\sigma ,A}}\right\}$$ 13

The composite energy *E*_12,UKS_ employs a subtractive scheme, that is, the low-level method is utilized on the supersystem, and the difference of the low- and high-level energies are taken in the case of the active subsystem:

$$E_{12,\mathrm{UKS}}=E_{2,\mathrm{UKS}}[D^{\alpha ,\mathrm{AB}},\;D^{\beta ,\mathrm{AB}}]-E_{2,\mathrm{UKS}}[D^{\alpha ,A},\;D^{\beta ,A}]+E_{1,\mathrm{UKS}}[{\tilde{D}}^{\alpha ,A},\;{\tilde{D}}^{\beta ,A}]+V_{\mathrm{nn}}$$ 14

where the matrices with the tilde are obtained with a high-level method. In general, the above energy terms of the low- and high-level methods can be written as

$$\begin{array}{cc} E_{\Lambda ,\mathrm{UKS}}[D^{\alpha },\;D^{\beta }]=\mathrm{Tr}\{h_{\mathrm{core}}(D^{\alpha }+D^{\beta })\}+\frac{1}{2}\mathrm{Tr}\{(D^{\alpha }+D^{\beta })J[D^{\alpha }+D^{\beta }]\}-a_{\mathrm{HF},\Lambda }\frac{1}{2}\mathrm{Tr}\{D^{\alpha }K[D^{\alpha }]+D^{\beta }K[D^{\beta }]\}+(1-a_{\mathrm{HF},\Lambda })E_{\mathrm{xc},\Lambda }[D^{\alpha },\;D^{\beta }] & \Lambda \in \{1,\;2\} \end{array}$$ 15

It should be pointed out about the notation that *E*_1,UKS_[**D̃** ^A^, ^α^ **D̃** ^A,β^] also implicitly depends on **D** ^α,AB^ and **D** ^β,AB^, but this is omitted throughout the article. In the following, a concise version of the evaluation of eq 13 is presented, while a more detailed description of the algorithm is provided in the Appendix.

In the first step of the spin-unrestricted Huzinaga embedding procedure, *E*_2,UKS_[**D** ^α,AB^, **D** ^β,AB^] is minimized by solving the conventional KS equations for the supersystem at the low-level

$$F_{2,\mathrm{UKS}}^{\sigma }[D^{\alpha ,\mathrm{AB}},\;D^{\beta ,\mathrm{AB}}]C_{i}^{\sigma }=\sum_{j}SC_{j}^{\sigma }(\epsilon_{2,\mathrm{UKS}}^{\sigma }{)}_{ji}$$ 16

where **F**_2, UKS_^σ^ is the Fock (Kohn–Sham) matrix

$$F_{2,\mathrm{UKS}}^{\sigma }[D^{\alpha ,\mathrm{AB}},\;D^{\beta ,\mathrm{AB}}]=h_{\mathrm{core}}+J[D^{\alpha ,\mathrm{AB}}+D^{\beta ,\mathrm{AB}}]-a_{\mathrm{HF},2}K[D^{\sigma ,\mathrm{AB}}]+(1-a_{\mathrm{HF},2})V_{\mathrm{xc},2}^{\sigma }[D^{\alpha ,\mathrm{AB}},\;D^{\beta ,\mathrm{AB}}]$$ 17

Next, the orbitals with alpha and beta spin localized separately. The resulting localized MOs are assigned to either the active or the environment subsystems, and the corresponding density matrices of the subsystems, **D** ^σ,A^ and **D** ^σ,B^, are constructed. Subsequently, the subsystem energy at the low level, *E*_2,UKS_[**D** ^α,A^, **D** ^β,A^], and the first part of the correction term, $\underset{\sigma }{\sum }\mathrm{Tr}\left\{-D^{\sigma ,A}\frac{\partial E_{12,\mathrm{UKS}}}{\partial D^{\sigma ,A}}\right\}$ , are evaluated. The orbitals of the active subsystem are then reoptimized in the presence of the embedding potential using the Huzinaga-equation

$${\overset{\sim }{H}}_{\mathrm{UKS}}^{\sigma }[{\overset{\sim }{D}}^{\alpha ,A},\;{\overset{\sim }{D}}^{\beta ,A}]{\overset{\sim }{C}}_{i}^{\sigma ,A}=\underset{j}{\sum }S{\overset{\sim }{C}}_{j}^{\sigma ,A}({\overset{\sim }{\epsilon }}_{\mathrm{UKS}}^{\sigma }{)}_{ji}$$ 18

where **C̃** ^**σ**,A^ contains the coefficients of the reoptimized MOs and (ϵ̃_UKS_^σ^)_*ji*_ are the corresponding Lagrange multipliers. The spin-dependent Huzinaga operator, **H̃**_UKS_^σ^, is constructed from the embedded Fock matrix, **F̃** ^σ,A^, the reoptimized density matrix, **D̃** ^σ,A^, and the projector of the environment subsystem, **R** ^σ,B^ = **D** ^σ,B^

$${\tilde{H}}_{\mathrm{UKS}}^{\sigma }[{\tilde{D}}^{\alpha ,A},\;{\tilde{D}}^{\beta ,A}]={\tilde{F}}_{\mathrm{UKS}}^{\sigma }[{\tilde{D}}^{\alpha ,A},\;{\tilde{D}}^{\beta ,A}]-{\tilde{F}}_{\mathrm{UKS}}^{\sigma }[{\tilde{D}}^{\alpha ,A},\;{\tilde{D}}^{\beta ,A}]R^{\sigma ,B}S-SR^{\sigma ,B}{\tilde{F}}_{\mathrm{UKS}}^{\sigma }[{\tilde{D}}^{\alpha ,A},\;{\tilde{D}}^{\beta ,A}]$$ 19

$${\tilde{F}}_{\mathrm{UKS}}^{\sigma }[{\tilde{D}}^{\alpha ,A},\;{\tilde{D}}^{\beta ,A}]=F_{2,\mathrm{UKS}}^{\sigma }[D^{\alpha ,\mathrm{AB}},\;D^{\beta ,\mathrm{AB}}]-F_{2,\mathrm{UKS}}^{\sigma }[D^{\alpha ,A},\;D^{\beta ,A}]+F_{1,\mathrm{UKS}}^{\sigma }[{\tilde{D}}^{\alpha ,A},\;{\tilde{D}}^{\beta ,A}]$$ 20

Solving eq 18 yields spin–orbitals for subsystem A that minimize *E*_PbE,UKS_, while preserving orthonormality between the active and environment orbitals of the same-spin. As a final step, the subsystem energy at the high-level, *E*_1,UKS_, and the second part of the correction term, $\mathrm{Tr}\left\{{\tilde{D}}^{\sigma ,A}\frac{\partial E_{12,\mathrm{UKS}}}{\partial D^{\sigma ,A}}\right\}$ , can be evaluated with the reoptimized spin–orbitals. If a post-Hartree–Fock approach is utilized as the high-level method, the final energy can be evaluated as

$$E_{\mathrm{U‐tot}}=E_{\mathrm{PbE},\mathrm{UKS}}+E_{\mathrm{cor}}[{\tilde{C}}^{\alpha ,A},\;{\tilde{C}}^{\beta ,A}]$$ 21

where *E*_cor_ is the correlation energy.

### 2.3 Restricted Open-Shell Huzinaga Embedding

#### 2.3.1 RO Energy and Eigenvalue Equations

Before the discussion of the spin-restricted open-shell projection-based embedding, let us recapitulate the ROKS scheme of Filatov and Shaik^49,50^ and the ROHF approach of Roothaan,^48^ where the orbitals are spatially constrained to be the same for different spins, that is, **C** ^α^ = **C** ^β^, therefore, the spin-indices of the coefficient matrices can be omitted in this formalism. *k*, *l* indices will be used for the MOs of the closed-shell (c), and *m*, *n* are reserved for the open-shell (o) part of the occupied space. In the RO scheme, the total density matrix, **D**, can be considered as an ensemble density matrix that can be represented as the linear combination of microstate (*L*) density matrices

$$D=\underset{L}{\sum }c_{L}\left(D_{L}^{\alpha }+D_{L}^{\beta }\right)$$ 22

where *c*_*L*_ are the coefficients of the microstates with the constraint ∑_*L*_ *c*_*L*_ = 1, and the spin-dependent density matrices of a microstate, **D**_*L*_^σ^, can be constructed from the closed-shell (**D** ^c^) and open-shell (**D**_*L*_^o, σ^) densities:

$$D_{L}^{\sigma }=\frac{1}{2}D_{L}^{c}+D_{L}^{o,\sigma }$$ 23

$$D_{L}^{c}=\underset{k}{\sum }n_{k,L}C_{k}{\left(C_{k}\right)}^{T}=\underset{k}{\sum }n_{k}R_{k}$$ 24

$$D_{L}^{o,\sigma }=\underset{m}{\sum }n_{m,L}^{\sigma }C_{m}{\left(C_{m}\right)}^{T}=\underset{m}{\sum }n_{m,L}^{\sigma }R_{m}$$ 25

The closed-shell density is the same in all microstates, that is, **D**_*L*_^c^ = **D** ^c^ and *n*_*k*,*L*_ = *n*_*k*_ = *n*_*k*_^α^ + *n*_*k*_^β^. The occupancy of the orbitals are *n*_*k*_ ∈{2} and *n*_*m*,*L*_^σ^ ∈{0, 1}, thus the occupancy of a given open-shell orbital can be fractional in the ensemble, which can be expressed with the

$$f_{m}=\frac{1}{2}\underset{L}{\sum }c_{L}\left(n_{m,L}^{\alpha }+n_{m,L}^{\beta }\right)$$ 26

fractional average occupation number. As a consequence of the above definitions, the total density can also be written as the sum of closed-shell and open-shell densities:

$$D=D^{c}+D^{o}=D^{c}+\sum_{L}c_{L}D_{L}^{o}=D^{c}+2\sum_{m}f_{m}C_{m}(C_{m}{)}^{T}$$ 27

Throughout the article, it will be assumed that the number of closed- and open-shell orbitals, the microstate coefficients, and the occupancy of the microstates are fixed. The ensemble density matrix is spin-adapted, while a microstate may possess mixed spin-symmetry, and it is assumed that it can be represented with a single determinant. The latter assumption is not guaranteed for all multiplets, thus our discussion is restricted to systems where the theory is valid, that is, specific electron configurations of atoms and linear molecules, and systems with a single open-shell where the degenerate open-shell orbitals are half-filled and all the spins are parallel. The latter case, i.e., high-spin states of molecules, is the focus of this study.

The total energy in the RO scheme can be written as the linear combination of microstate energies,

$$E_{\Lambda ,\mathrm{ROKS}}\left[C\right]=\underset{L}{\sum }c_{L}E_{\Lambda ,\mathrm{ROKS},L}\left[C\right]+V_{\mathrm{nn}}$$ 28

The energy of a microstate can be written as

$$E_{\Lambda ,\mathrm{ROKS},L}\left[C\right]=\mathrm{Tr}\left\{\underset{k}{\sum }n_{k}{\left(C_{k}\right)}^{T}h_{\mathrm{core}}C_{k}+\frac{1}{2}\underset{kl}{\sum }n_{k}n_{l}{\left(C_{l}\right)}^{T}\left(J\left[C_{k}{\left(C_{k}\right)}^{T}\right]-\frac{1}{2}a_{\mathrm{HF},\Lambda }K\left[C_{k}{\left(C_{k}\right)}^{T}\right]\right)C_{l}\right\}+\mathrm{Tr}\left\{\underset{m}{\sum }\left(n_{m,L}^{\alpha }+n_{m,L}^{\beta }\right){\left(C_{m}\right)}^{T}h_{\mathrm{core}}C_{m}\right\}+\frac{1}{2}\underset{mn}{\sum }\mathrm{Tr}\left\{\left(n_{m,L}^{\alpha }+n_{m,L}^{\beta }\right)\left(n_{n,L}^{\alpha }+n_{n,L}^{\beta }\right){\left(C_{m}\right)}^{T}J\left[C_{n}{\left(C_{n}\right)}^{T}\right]C_{m}\right\}-\frac{1}{2}\underset{mn}{\sum }\mathrm{Tr}\left\{\left(n_{m,L}^{\alpha }n_{n,L}^{\alpha }+n_{m,L}^{\beta }n_{n,L}^{\beta }\right)\frac{1}{2}a_{\mathrm{HF},\Lambda }{\left(C_{m}\right)}^{T}K\left[C_{n}{\left(C_{n}\right)}^{T}\right]C_{m}\right\}+\mathrm{Tr}\left\{\underset{k}{\sum }\underset{m}{\sum }n_{k}\left(n_{m,L}^{\alpha }+n_{m,L}^{\beta }\right){\left(C_{m}\right)}^{T}\left(J\left[C_{k}{\left(C_{k}\right)}^{T}\right]-\frac{1}{2}a_{\mathrm{HF},\Lambda }K\left[C_{k}{\left(C_{k}\right)}^{T}\right]\right)C_{m}\right\}+\left(1-a_{\mathrm{HF},\Lambda }\right)E_{\mathrm{xc},\Lambda }\left[D_{L}^{\alpha },D_{L}^{\beta }\right]$$ 29

or equivalently, the ensemble energy can be formulated directly as

$$E_{\Lambda ,\mathrm{ROKS}}\left[C\right]=\mathrm{Tr}\left\{\underset{k}{\sum }n_{k}{\left(C_{k}\right)}^{T}h_{\mathrm{core}}C_{k}+\frac{1}{2}\underset{kl}{\sum }n_{k}n_{l}{\left(C_{l}\right)}^{T}\left(J\left[C_{k}{\left(C_{k}\right)}^{T}\right]-\frac{1}{2}a_{\mathrm{HF},\Lambda }K\left[C_{k}{\left(C_{k}\right)}^{T}\right]\right)C_{l}\right\}+\mathrm{Tr}\left\{\underset{m}{\sum }2f_{m}{\left(C_{m}\right)}^{T}h_{\mathrm{core}}C_{m}+\frac{1}{2}\underset{mn}{\sum }2f_{m}2f_{n}{\left(C_{m}\right)}^{T}\left(a_{mn}J\left[C_{n}{\left(C_{n}\right)}^{T}\right]-b_{mn}\frac{1}{2}a_{\mathrm{HF},\Lambda }K\left[C_{n}{\left(C_{n}\right)}^{T}\right]\right)C_{m}\right\}+\mathrm{Tr}\left\{\underset{k}{\sum }\underset{m}{\sum }n_{k}2f_{m}{\left(C_{m}\right)}^{T}\left(J\left[C_{k}{\left(C_{k}\right)}^{T}\right]-\frac{1}{2}a_{\mathrm{HF},\Lambda }K\left[C_{k}{\left(C_{k}\right)}^{T}\right]\right)C_{m}\right\}+\underset{L}{\sum }c_{L}\left(1-a_{\mathrm{HF},\Lambda }\right)E_{\mathrm{xc},\Lambda }\left[D_{L}^{\alpha },D_{L}^{\beta }\right]+V_{\mathrm{nn}}$$ 30

where the first term on the right side of the equation is the kinetic, nuclear-electron attraction, Coulomb, and exchange energy that related to the closed shells, the second term comprises the corresponding contributions for the open shells, the third term is the Coulomb and exchange interaction energy of the subshells, and the final term incorporates the exchange–correlation part of all shells, including their interactions. *a*_*mn*_ and *b*_*mn*_ are orbital-dependent coupling parameters:

$$a_{mn}=\frac{1}{4f_{m}f_{n}}\underset{L}{\sum }c_{L}\left(n_{m,L}^{\alpha }+n_{m,L}^{\beta }\right)\left(n_{n,L}^{\alpha }+n_{n,L}^{\beta }\right)$$ 31

$$b_{mn}=\frac{1}{4f_{m}f_{n}}\underset{L}{\sum }c_{L}\left(n_{m,L}^{\alpha }n_{n,L}^{\alpha }+n_{m,L}^{\beta }n_{n,L}^{\beta }\right)$$ 32

In those systems, where the above-described single determinant description is valid, the open-shell orbitals share a common occupation number, thus, *f*_*n*_ = *f*_*m*_, and the indices of *f* can be dropped. Our goal is to describe large molecules with high-spin states hence $f=\frac{1}{2}$ , moreover, if positive microstate coefficients are assumed, then *a*_*mn*_ = *a* = 1, *b*_*mn*_ = *b* = 2, where *a* and *b* are the orbital-independent coupling parameters of Roothaan. Finally, the ensemble energy can be written in its most simple form as a function of the closed- and open-shell densities:

$$E_{\Lambda ,\mathrm{ROKS}}\left[D\right]=\mathrm{Tr}\left\{D^{c}h_{\mathrm{core}}+\frac{1}{2}D^{c}\left(J\left[D^{c}\right]-\frac{1}{2}a_{\mathrm{HF},\Lambda }K\left[D^{c}\right]\right)\right\}+\mathrm{Tr}\left\{D^{o}h_{\mathrm{core}}+\frac{1}{2}D^{o}\left(aJ\left[D^{o}\right]-b\frac{1}{2}a_{\mathrm{HF},\Lambda }K\left[D^{o}\right]\right)\right\}+\mathrm{Tr}\left\{D^{o}\left(J\left[D^{c}\right]-\frac{1}{2}a_{\mathrm{HF},\Lambda }K\left[D^{c}\right]\right)\right\}+\underset{L}{\sum }c_{L}\left(1-a_{\mathrm{HF},\Lambda }\right)E_{\mathrm{xc},\Lambda }\left[D_{L}^{\alpha },D_{L}^{\beta }\right]+V_{\mathrm{nn}}$$ 33

To obtain orbitals that minimize the energy in eq 33 under the constraint that the orbitals are mutually orthogonal, normalized, and the occupation numbers and microstate coefficients are fixed, one has to use the following Lagrangian:

$$L_{\Lambda ,\mathrm{ROKS}}=E_{\Lambda ,\mathrm{ROKS}}\left[D\right]-2\mathrm{Tr}\left\{\left(C^{T}SC-1\right)\epsilon_{\Lambda ,\mathrm{ROKS}}\right\}$$ 34

where **ϵ**_Λ,ROKS_ holds the corresponding multipliers. Minimizing the above expression with respect to C_μ*k*_ and C_μ*m*_ results in

$$F_{\Lambda ,\mathrm{ROKS}}^{c}\left[D\right]C_{k}=\underset{l}{\sum }SC_{l}{\left(\epsilon_{\Lambda ,\mathrm{ROKS}}\right)}_{lk}+\underset{n}{\sum }SC_{n}{\left(\epsilon_{\Lambda ,\mathrm{ROKS}}\right)}_{nk}$$ 35

$$fF_{\Lambda ,m,\mathrm{ROKS}}^{o}\left[D\right]C_{m}=\underset{n}{\sum }SC_{n}{\left(\epsilon_{\Lambda ,\mathrm{ROKS}}\right)}_{nm}+\underset{l}{\sum }SC_{l}{\left(\epsilon_{\Lambda ,\mathrm{ROKS}}\right)}_{lm}$$ 36

where eqs 35 and 36 are the equations of the closed-shell and open-shell orbitals, respectively, and the corresponding Fock matrices can be written as

$$F_{\Lambda ,\mathrm{ROKS}}^{c}\left[D\right]=h_{\mathrm{core}}+J\left[D^{c}\right]-a_{\mathrm{HF},\Lambda }\frac{1}{2}K\left[D^{c}\right]+J\left[D^{o}\right]-a_{\mathrm{HF},\Lambda }\frac{1}{2}K\left[D^{o}\right]+\left(1-a_{\mathrm{HF},\Lambda }\right)V_{\mathrm{xc},\Lambda }^{c}\left[D\right]$$ 37

$$F_{\Lambda ,m,\mathrm{ROKS}}^{o}\left[C\right]=h_{\mathrm{core}}+aJ\left[D^{o}\right]-a_{\mathrm{HF},\Lambda }b\frac{1}{2}K\left[D^{o}\right]+J\left[D^{c}\right]-a_{\mathrm{HF},\Lambda }\frac{1}{2}K\left[D^{c}\right]+\left(1-a_{\mathrm{HF},\Lambda }\right)V_{\mathrm{xc},\Lambda ,m}^{o}\left[D\right]$$ 38

and the exchange–correlation potentials are defined as

$$V_{\mathrm{xc},\Lambda }^{c}[D]=\frac{1}{2}\sum_{L}c_{L}(V_{\mathrm{xc},\Lambda ,L}^{\alpha }[D_{L}^{\alpha },\;D_{L}^{\beta }]+V_{\mathrm{xc},\Lambda ,L}^{\beta }[D_{L}^{\alpha },\;D_{L}^{\beta }])$$ 39

$$V_{\mathrm{xc},\Lambda ,m}^{o}[D]=\frac{1}{2f}\sum_{L}c_{L}(n_{m,L}^{\alpha }V_{\mathrm{xc},\Lambda ,L}^{\alpha }[D_{L}^{\alpha },\;D_{L}^{\beta }]+n_{m,L}^{\beta }V_{\mathrm{xc},\Lambda ,L}^{\beta }[D_{L}^{\alpha },\;D_{L}^{\beta }])$$ 40

$$V_{\mathrm{xc},\Lambda ,L}^{\sigma }[D_{L}^{\alpha },\;D_{L}^{\beta }]=\frac{\partial E_{\mathrm{xc},\Lambda }[D_{L}^{\alpha },\;D_{L}^{\beta }]}{\partial D_{L}^{\sigma }}$$ 41

Note that **V**_xc,Λ,*m*_^o^ is the same for all *m* in the case of high-spin states and will be denoted by **V**_xc,Λ_^o^. The Fock operators of the different shells have different forms, and the simultaneous iterative diagonalization procedure of the two shell operators would increase the cost of the calculations. However, it is possible to derive a unified equation with the use of the shell-coupling operators introduced by Roothaan, see the Appendix for more details. Here, only the final pseudoeigenvalue equation of the previous articles^48–50^ are shown, that is

$$F_{\Lambda ,\mathrm{ROKS}}\left[D\right]C_{i}^{\prime }=\frac{1}{\omega_{i}}\underset{j}{\sum }SC_{j}^{\prime }{\left(\epsilon_{\Lambda ,\mathrm{ROKS}}^{\prime }\right)}_{ji}$$ 42

where the MOs of the unified equation are related to the MOs of the subshell equations by a unitary transformation. Therefore, **ϵ**′_Λ,ROKS_ is different from the original multiplier matrix, **ϵ**_Λ,ROKS_, and **C**_*i*_ *′* is also equivalent to but not identical with **C**_*i*_. The unified ROKS Fock matrix, **F**_Λ,ROKS_, is defined as

$$F_{\Lambda ,\mathrm{ROKS}}\left[D\right]=F_{\Lambda }^{c}\left[D\right]-Q_{\Lambda }\left[D^{o}\right]+T_{\Lambda }\left[D\right]$$ 43

$$F_{\Lambda }^{c}\left[D\right]=h_{\mathrm{core}}+J\left[D\right]-a_{\mathrm{HF},\Lambda }\frac{1}{2}K\left[D\right]+\left(1-a_{\mathrm{HF},\Lambda }\right)V_{\mathrm{xc},\Lambda }^{c}\left[D\right]$$ 44

$$Q_{\Lambda }[D^{o}]=\overset{-}{a}J[D^{o}]-a_{\mathrm{HF},\Lambda }\overset{-}{b}\frac{1}{2}K[D^{o}]$$ 45

$$T_{\Lambda }[D]=SR^{c}Q_{\Lambda }[D^{o}]+Q_{\Lambda }[D^{o}]R^{c}S+f(SR^{o}Q_{\Lambda }[D^{o}]+Q_{\Lambda }[D^{o}]R^{o}S)+(1-a_{\mathrm{HF},\Lambda })\overset{-}{c}\gamma^{T}(V_{\mathrm{xc},\Lambda }^{c}[D]-V_{\mathrm{xc},\Lambda }^{o}[D])R^{o}S+(1-a_{\mathrm{HF},\Lambda })\overset{-}{c}SR^{o}(V_{\mathrm{xc},\Lambda }^{c}[D]-V_{\mathrm{xc},\Lambda }^{o}[D])\gamma$$ 46

and the corresponding projectors and parameters are

$$\gamma =R^{c}S-\frac{1}{\overset{-}{c}}1+\frac{1}{2\overset{-}{c}}R^{o}S$$ 47

$$\begin{array}{cc} R^{c}=\sum_{k}R_{k}, & R^{o}=\sum_{m}R_{m} \end{array}$$ 48

$$\begin{array}{l} \overset{-}{a}=\frac{1-a}{1-f},\overset{-}{b}=\frac{1-b}{1-f},\overset{-}{c}=\frac{1}{1-f},\\ \omega_{i}=\underset{k}{\sum }\delta_{ki}+\underset{m}{\sum }\delta_{mi}f \end{array}$$ 49

where δ_*ij*_ is the Kronecker delta.

As we mentioned in the introduction, the RO method is more efficient than the unrestricted approach. We would like to note here, however, that significant computational savings are only achieved with methods that also include exact exchange contributions. For pure DFT techniques, the ROKS approach still requires the calculation of both the alpha and the beta exchange–correlation potential matrices as well as the **T** matrix, and the treatment of the Coulomb potential has similar computational demands as well.

#### 2.3.2 Multilevel Energy and Eigenvalue Equations

In the RO version of the Huzinaga embedding, the energy can be written as

$$E_{\mathrm{PbE},\mathrm{ROKS}}=E_{12,\mathrm{ROKS}}+\mathrm{Tr}\left\{\left({\overset{\sim }{D}}^{c,A}-D^{c,A}\right)\frac{\partial E_{12,\mathrm{ROKS}}}{\partial D^{c,A}}+\left({\overset{\sim }{D}}^{o,A}-D^{o,A}\right)\frac{\partial E_{12,\mathrm{ROKS}}}{\partial D^{o,A}}\right\}$$ 50

and the combined energy expression is the same as eq 14:

$$E_{12,\mathrm{ROKS}}=E_{2,\mathrm{ROKS}}[D^{\mathrm{AB}}]-E_{2,\mathrm{ROKS}}[D^{A}]+E_{1,\mathrm{ROKS}}[{\tilde{D}}^{A}]+V_{\mathrm{nn}}$$ 51

except that the energy terms have different definitions. Again, the starting point of the embedding scheme is to minimize the supersystem energy at the low-level with the same constrains that mentioned in the previous section, therefore, the following Lagrangian is introduced:

$$L_{2,\mathrm{ROKS}}^{\mathrm{AB}}=E_{2,\mathrm{ROKS}}\left[D^{\mathrm{AB}}\right]-2\mathrm{Tr}\left\{\left({\left(C^{\mathrm{AB}}\right)}^{T}SC^{\mathrm{AB}}-1\right)\epsilon_{2,\mathrm{ROKS}}\right\}$$ 52

where **ϵ**_2,ROKS_ is the matrix of the corresponding multipliers, and if the above Lagrangian is minimized with respect C_μ*k*_ and C_μ*m*_, we get the equations of the low-level method for the supersystem after the transformations outlined in the Appendix:

$$F_{2,\mathrm{ROKS}}\left[D^{\mathrm{AB}}\right]C_{i}^{\prime ,\mathrm{AB}}=\frac{1}{\omega_{i}}\underset{j}{\sum }SC_{j}^{\prime ,\mathrm{AB}}{\left(\epsilon_{2,\mathrm{ROKS}}^{\prime }\right)}_{ji}$$ 53

Optimal orbitals can be obtained by the iterative diagonalization of **F**_2,ROKS_[**D** ^AB^], and the *E*_2,ROKS_[**D** ^AB^] term is evaluated after self-consistency is reached. Next, the orbitals of the closed and open shells are localized separately as

$$\begin{array}{l} L_{k}^{c}=\sum_{l}C_{l}^{\prime ,\mathrm{AB}}W_{lk}^{c}\\ L_{m}^{o}=\sum_{n}C_{n}^{\prime ,\mathrm{AB}}W_{nm}^{o} \end{array}$$ 54

where **W** ^c^ and **W** ^o^ are the unitary transformation matrices of the closed and open shells, respectively, and **L** holds the coefficients of the localized MOs. Subsequently, the localized orbitals are assigned to the environment and active subsystems as it was mentioned in the UHF-case:

$$\begin{array}{l} L^{c}=L^{c,A}\cup L^{c,B},\\ L^{o}=L^{o,A} \end{array}$$ 55

where **L** ^c,A^ and **L** ^c,B^ are the active and environment blocks of **L** ^c^, and it is assumed that the orbitals of the open shell are always localized in the active subsystem. After the system is separated, the subsystem density matrices are calculated using

$$\begin{array}{cc} D^{c,S}=2\sum_{k\in S}L_{k}(L_{k}{)}^{T} & S\in \{A,\;B\} \end{array}$$ 56

$$D^{o,A}=2f\underset{n\in A}{\sum }L_{n}{\left(L_{n}\right)}^{T}$$ 57

$$D^{A}=D^{c,A}+D^{o,A}$$ 58

to evaluate *E*_2,ROKS_[**D** ^A^]. Next, the embedding potential can be derived from the derivative of the composite energy as

$$\frac{\partial E_{12,\mathrm{ROKS}}}{\partial D^{c,A}}=J[D^{c,\mathrm{AB}}]-J[D^{c,A}]-a_{\mathrm{HF},2}\frac{1}{2}(K[D^{c,\mathrm{AB}}]-K[D^{c,A}])+\frac{1}{2}(1-a_{\mathrm{HF},2})\underset{L}{\sum }c_{L}(V_{\mathrm{xc},2,L}^{\alpha ,\mathrm{AB}}[D_{L}^{\alpha ,\mathrm{AB}},\;D_{L}^{\beta ,\mathrm{AB}}]+\frac{1}{2}(1-a_{\mathrm{HF},2})\underset{L}{\sum }c_{L}(V_{\mathrm{xc},2,L}^{\beta ,\mathrm{AB}}[D_{L}^{\alpha ,\mathrm{AB}},\;D_{L}^{\beta ,\mathrm{AB}}]-\frac{1}{2}(1-a_{\mathrm{HF},2})\underset{L}{\sum }c_{L}V_{\mathrm{xc},2,L}^{\alpha ,A}[D_{L}^{\alpha ,A},\;D_{L}^{\beta ,A}])-\frac{1}{2}(1-a_{\mathrm{HF},2})\underset{L}{\sum }c_{L}(V_{\mathrm{xc},2,L}^{\beta ,A}[D_{L}^{\alpha ,A},\;D_{L}^{\beta ,A}])$$ 59

$$\frac{\partial E_{12,\mathrm{ROKS}}}{\partial D^{o,A}}=J[D^{c,\mathrm{AB}}]-J[D^{c,A}]-a_{\mathrm{HF},2}\frac{1}{2}(K[D^{c,\mathrm{AB}}]-K[D^{c,A}])+\frac{1}{2}(1-a_{\mathrm{HF},2})\underset{L}{\sum }c_{L}(V_{\mathrm{xc},2,L}^{\alpha ,\mathrm{AB}}[D_{L}^{\alpha ,\mathrm{AB}},\;D_{L}^{\beta ,\mathrm{AB}}]-\frac{1}{2}(1-a_{\mathrm{HF},2})\underset{L}{\sum }c_{L}V_{\mathrm{xc},2,L}^{\alpha ,A}[D_{L}^{\alpha ,A},\;D_{L}^{\beta ,A}])$$ 60

where it is exploited that $D^{\alpha }=\frac{1}{2}D^{c}+D^{o}$ and $D^{\beta }=\frac{1}{2}D^{c}$ , which are valid relations in the case of spatially restricted single determinant description of high-spin states. After the embedding potential is constructed, the first part of the correction term, $\mathrm{Tr}\left\{-D^{c,A}\frac{\partial E_{12,\mathrm{ROKS}}}{\partial D^{c,A}}-D^{o,A}\frac{\partial E_{12,\mathrm{ROKS}}}{\partial D^{o,A}}\right\}$ , can be evaluated.

To obtain orbitals that are optimal for the energy that is computed with a high-level method in the presence of the embedding potential while maintaining orthogonality of the subsystems, the following Lagrangian is defined:

$${\overset{\sim }{L}}_{\mathrm{ROKS}}^{A}=E_{\mathrm{PbE},\mathrm{ROKS}}-\mathrm{Tr}\{2(({\overset{\sim }{C}}^{A}{)}^{T}S{\overset{\sim }{C}}^{A}-1){\overset{\sim }{\epsilon }}_{\mathrm{ROKS}}+4(({\overset{\sim }{C}}^{A}{)}^{T}SL^{c,B}){\overset{\sim }{\eta }}_{\mathrm{ROKS}}\}$$ 61

where ${\tilde{\epsilon }}_{\mathrm{ROKS}}$ and ${\tilde{\eta }}_{\mathrm{ROKS}}$ stand for the corresponding Lagrange multiplier matrices. If the above equation is minimized with respect to **C̃**_μ*k*_^A^ and **C̃**_μ*m*_^A^, the following equations can be written down for the closed and open shells, respectively:

$${\tilde{F}}_{\mathrm{ROKS}}^{c}[{\tilde{D}}^{A}]{\tilde{C}}_{k}^{A}=\sum_{l}S{\tilde{C}}_{l}^{A}({\tilde{\epsilon }}_{\mathrm{ROKS}}{)}_{lk}+\sum_{n}S{\tilde{C}}_{n}^{A}({\tilde{\epsilon }}_{\mathrm{ROKS}}{)}_{nk}+\sum_{l}SL_{l}^{c,B}({\tilde{\eta }}_{\mathrm{ROKS}}{)}_{lk}$$ 62

$$f{\tilde{F}}_{\mathrm{ROKS}}^{o}[{\tilde{D}}^{A}]{\tilde{C}}_{m}^{A}=\sum_{n}S{\tilde{C}}_{n}^{A}({\tilde{\epsilon }}_{\mathrm{ROKS}}{)}_{nm}+\sum_{l}S{\tilde{C}}_{l}^{A}({\tilde{\epsilon }}_{\mathrm{ROKS}}{)}_{lm}+\sum_{l}SL_{l}^{c,B}({\tilde{\eta }}_{\mathrm{ROKS}}{)}_{lm}$$ 63

**F̃**_ROKS_^c^ and **F̃**_ROKS_^o^ are the closed- and open-shell Fock matrices of the high-level method, respectively, that are embedded in the potential of the environment. These operators can be written as

$${\tilde{F}}_{\mathrm{ROKS}}^{c}[{\tilde{D}}^{A}]=h_{\mathrm{core},\mathrm{ROKS}}^{A‐\mathrm{in}‐B}+J[{\tilde{D}}^{c,A}]-\frac{1}{2}a_{\mathrm{HF},1}K[{\tilde{D}}^{c,A}]+J[{\tilde{D}}^{o}]-a_{\mathrm{HF},1}\frac{1}{2}K[{\tilde{D}}^{o}]+(1-a_{\mathrm{HF},1}){\tilde{V}}_{\mathrm{xc}}^{c}[{\tilde{D}}^{A}]$$ 64

$${\tilde{F}}_{\mathrm{ROKS}}^{o}[{\tilde{D}}^{A}]=h_{\mathrm{core},\mathrm{ROKS}}^{A‐\mathrm{in}‐B}+aJ[{\tilde{D}}^{o}]-a_{\mathrm{HF},1}b\frac{1}{2}K[{\tilde{D}}^{o}]+J[{\tilde{D}}^{c,A}]-\frac{1}{2}a_{\mathrm{HF},1}K[{\tilde{D}}^{c,A}]+(1-a_{\mathrm{HF},1}){\tilde{V}}_{\mathrm{xc}}^{o}[{\tilde{D}}^{A}]$$ 65

where part of the embedding potential is incorporated into the core Hamiltonian:

$$h_{\mathrm{core},\mathrm{ROKS}}^{A‐\mathrm{in}‐B}=h_{\mathrm{core}}+J[D^{\mathrm{AB}}]-J[D^{A}]-a_{\mathrm{HF},2}\frac{1}{2}(K[D^{\mathrm{AB}}]-K[D^{A}])$$ 66

and the exchange–correlation part of the embedding potential is added to the closed-shell and open-shell exchange–correlation potentials:

$${Ṽ}_{\mathrm{xc}}^{c}[{\overset{\sim }{D}}^{A}]=\frac{1}{2}\underset{L}{\sum }c_{L}\left(V_{\mathrm{xc},1,L}^{\alpha }[{\overset{\sim }{D}}_{L}^{\alpha ,A},\;{\overset{\sim }{D}}_{L}^{\beta ,A}]\right)+\frac{1}{2}\underset{L}{\sum }c_{L}\left(V_{\mathrm{xc},1,L}^{\beta }[{\overset{\sim }{D}}_{L}^{\alpha ,A},\;{\overset{\sim }{D}}_{L}^{\beta ,A}]\right)+\frac{1}{2}\underset{L}{\sum }c_{L}\left(V_{\mathrm{xc},2,L}^{\alpha }[D_{L}^{\alpha ,\mathrm{AB}},\;D_{L}^{\beta ,\mathrm{AB}}]\right)+\frac{1}{2}\underset{L}{\sum }c_{L}\left(V_{\mathrm{xc},2,L}^{\beta }[D_{L}^{\alpha ,\mathrm{AB}},\;D_{L}^{\beta ,\mathrm{AB}}]\right)-\frac{1}{2}\underset{L}{\sum }c_{L}\left(V_{\mathrm{xc},2,L}^{\alpha }[D_{L}^{\alpha ,A},\;D_{L}^{\beta ,A}]\right)-\frac{1}{2}\underset{L}{\sum }c_{L}\left(V_{\mathrm{xc},2,L}^{\beta }[D_{L}^{\alpha ,A},\;D_{L}^{\beta ,A}]\right)$$ 67

$${Ṽ}_{\mathrm{xc}}^{o}[{\tilde{D}}^{A}]=\frac{1}{2f}\sum_{L}c_{L}(V_{\mathrm{xc},1,L}^{\alpha }[{\tilde{D}}_{L}^{\alpha ,A},\;{\tilde{D}}_{L}^{\beta ,A}])+\frac{1}{2f}\sum_{L}c_{L}(1-a_{\mathrm{HF},2})(V_{\mathrm{xc},2,L}^{\alpha }[D_{L}^{\alpha ,\mathrm{AB}},\;D_{L}^{\beta ,\mathrm{AB}}]-V_{\mathrm{xc},2,L}^{\alpha }[D_{L}^{\alpha ,A},\;D_{L}^{\beta ,A}])$$ 68

Note that the embedded operators depend also on **D** ^AB^ and **D** ^A^, but this dependence will be omitted throughout the article. The resulting equations, eqs 62 and 63, are formally the same as eqs 35 and 36, respectively, except for the active-environment coupling terms, furthermore, the core-Hamiltonian and the exchange–correlation potentials are replaced with their embedded variants. Applying the transformations outlined in the Appendix, one can derive equations for the closed and open shells where the operators on the left side are the same:

$${\tilde{F}}_{\mathrm{ROKS}}[{\tilde{D}}^{A}]{\tilde{C}}_{i}^{\prime ,A}=\frac{1}{{\tilde{\omega }}_{i}}\sum_{j}S{\tilde{C}}_{j}^{\prime ,A}({\tilde{\epsilon }}_{\mathrm{ROKS}}^{\prime }{)}_{ji}+\sum_{l}SL_{l}^{c,B}({\tilde{\eta }}_{\mathrm{ROKS}}{)}_{li}$$ 69

**C̃** ^*′*,A^ differs from **C̃** ^A^ by a unitary transformation, ${\tilde{\epsilon }}_{\mathrm{ROKS}}^{\prime }$ is the corresponding matrix of multipliers, and **F̃**_ROKS_ is the unified embedded Fock matrix:

$${\tilde{F}}_{\mathrm{ROKS}}[{\tilde{D}}^{A}]={\tilde{F}}_{\mathrm{ROKS}}^{c}[{\tilde{D}}^{A}]-Q_{1}[{\tilde{D}}^{o,A}]+\tilde{T}[{\tilde{D}}^{A}]$$ 70

$${\tilde{F}}_{\mathrm{ROKS}}^{c}[{\tilde{D}}^{A}]=h_{\mathrm{core},\mathrm{ROKS}}^{A‐\mathrm{in}‐B}+J[{\tilde{D}}^{A}]-\frac{1}{2}a_{\mathrm{HF},1}K[{\tilde{D}}^{A}]+(1-a_{\mathrm{HF},1}){\tilde{V}}_{\mathrm{xc}}^{c}[{\tilde{D}}^{A}]$$ 71

$$\tilde{T}[{\tilde{D}}^{A}]=S{\tilde{R}}^{c,A}Q_{1}[D^{o,A}]+Q_{1}[{\tilde{D}}^{o,A}]{\tilde{R}}^{c,A}S+f(S{\tilde{R}}^{o,A}Q_{1}[{\tilde{D}}^{o,A}]+Q_{1}[{\tilde{D}}^{o,A}]{\tilde{R}}^{o,A}S)+(1-a_{\mathrm{HF},1})\overset{-}{c}{\tilde{\gamma }}^{T}({\tilde{V}}_{\mathrm{xc}}^{c}[{\tilde{D}}^{A}]-{\tilde{V}}_{\mathrm{xc}}^{o}[{\tilde{D}}^{A}]){\tilde{R}}^{o,A}S+(1-a_{\mathrm{HF},1})\overset{-}{c}S{\tilde{R}}^{o,A}({\tilde{V}}_{\mathrm{xc}}^{c}[{\tilde{D}}^{A}]-{\tilde{V}}_{\mathrm{xc}}^{o}[{\tilde{D}}^{A}])\tilde{\gamma }$$ 72

$$\tilde{\gamma }={\tilde{R}}^{c,A}S-\frac{1}{\mathrm{c̅}}+\frac{1}{2\overset{-}{c}}{\tilde{R}}^{o,A}S$$ 73

$${\tilde{\omega }}_{i}=\sum_{k\in A}\delta_{ki}+f\sum_{m\in A}\delta_{mi}$$ 74

Next, the coupling of the active and environment orbitals is expressed with the following equation:

$$({\tilde{F}}_{\mathrm{ROKS}}[{\tilde{D}}^{A}]R^{c,B}S-SR^{c,B}{\tilde{F}}_{\mathrm{ROKS}}[{\tilde{D}}^{A}]){\tilde{C}}_{k}^{\prime ,A}=\sum_{l}SL_{l}^{c,B}({\tilde{\eta }}_{\mathrm{ROKS}}{)}_{li}$$ 75

where the projector is

$$R^{c,B}=L^{c,B}{\left(L^{c,B}\right)}^{T}$$ 76

By subtracting eq 75 from eq 69, one can obtain the final equation of the multilevel ROKS scheme:

$${\tilde{H}}_{\mathrm{ROKS}}[{\tilde{D}}^{A}]{\tilde{C}}_{i}^{\prime ,A}=\frac{1}{{\tilde{\omega }}_{i}}\sum_{j}S{\tilde{C}}_{j}^{\prime ,A}({\tilde{\epsilon }}_{\mathrm{ROKS}}^{\prime }{)}_{ji}$$ 77

where the operator on the left side is the Huzinaga operator of the ROKS approach:

$${\tilde{H}}_{\mathrm{ROKS}}={\tilde{F}}_{\mathrm{ROKS}}[{\tilde{D}}^{A}]-{\tilde{F}}_{\mathrm{ROKS}}[{\tilde{D}}^{A}]R^{c,B}S-SR^{c,B}{\tilde{F}}_{\mathrm{ROKS}}[{\tilde{D}}^{A}]$$ 78

The iterative diagonalization of **H̃**_ROKS_ produces orbitals that minimize the energy of subsystem A in a frozen embedding potential at a high level while guaranteeing mutually orthonormal orbitals between the subsystems. Finally, *E*_1,ROKS_[**D̃** ^A^] can be evaluated with the optimal orbitals, and the remaining part of the correction term, $\mathrm{Tr}\left\{{\tilde{D}}^{c,A}\frac{\partial E_{12,\mathrm{ROKS}}}{\partial D^{c,A}}+{\tilde{D}}^{o,A}\frac{\partial E_{12,\mathrm{ROKS}}}{\partial D^{o,A}}\right\}$ . Lastly, if the high-level method utilizes a post-HF technique, the final energy can be written as

$$E_{\mathrm{RO}‐\mathrm{tot}}=E_{\mathrm{PbE},\mathrm{ROKS}}+E_{\mathrm{cor}}[{\tilde{C}}^{\prime ,A}]$$ 79

where *E*_cor_ is the correlation energy.

### 2.4 Combination of the Restricted and Unrestricted Approaches

While the above-discussed RO embedding scheme seems more advantageous compared to its unrestricted counterpart due to being free from spin contamination and having reduced computational cost, it has its own drawbacks. Obviously, it cannot handle spin polarization, which is essential in modeling electron spin resonance spectra, moreover, it implicitly relies on the choice of the open-shell projector, **R** ^o^. If it is assumed that radicals are high-energy entities, then eigenvalue-based open-shell selection is a reasonable choice for the projector, however, there are no guarantees that this assumption holds true. Additionally, the unrestricted Ansatz requires twice as many wave function parameters, therefore, the extra degrees of freedom could be beneficial during wave function optimization. Therefore, it would be desirable to exploit the benefits of both approaches.

The first use-case is the situation where spin-polarization is only required in the active subsystem, while the RO scheme is sufficient for the environment, and the RO wave function is obtainable for the supersystem. In the technique denoted as UKS-in-ROKS (UHF-in-ROKS), eq 53 is solved, then the resulting restricted orbitals are used to construct the unrestricted Fock operator of the supersystem (eq 82), and the low-level supersystem energy is evaluated using eq 15. Next, the closed-shell and open-shell orbitals are localized separately, and the algorithm proceeds with the steps of the standard UKS embedding scheme. Note that the UKS-in-ROKS approach offers only minor computational savings, as the bottleneck in multilevel methods is typically the high-level method on the active subsystem.

The second combined method is termed ROKS-in-UKS (ROHF-in-UKS), which can be very useful when the low-level supersystem wave function is not obtainable using the RO scheme. Here, eq 81 is solved first, then the natural orbitals of the total density matrix, **D** ^α,AB^ + **D** ^β,AB^, are computed, and these spatially restricted orbitals are used to construct the operator in eq 82. Then, the doubly and singly occupied orbitals are localized separately, and the MOs are sorted for the active and environment subsystems. Next, the low-level subsystem Fock matrix, **F**_2, UKS_^σ^[**D** ^α,A^, **D** ^β,A^], is built using the spatially restricted orbitals, followed by the evaluation of the low-level subsystem energy and the first part of the correction term. At this point, all the quantities that are required for the high-level part of the RO embedding scheme can be obtained, including the projector of the environment (eq 76), the embedded core-Hamiltonian (eq 66), and the exchange–correlation embedding potential (eqs 67 and 68), thus one can proceed with the RO algorithm as discussed in Section 2.3.2.

## 3 Computational Details

The unrestricted and restricted open-shell versions of the Huzinaga-equation-based PbE have been implemented in the Mrcc program suite^55^ and will be included in its next release. All calculations were carried out using Mrcc, except for the semiempirical quantum mechanical (SQM) methods, for which Mrcc was interfaced with the xTB program developed by Grimme and co-workers.^56,57^

The assessment of multilevel techniques employed a range of WFT and DFT approaches. Among the WFT methods, the local natural orbital (LNO)-based CCSD­(T) was used,^6,58–60^ along with the local MP2 (LMP2) theory.^61^ Local correlation techniques employed the Boys–Foster localization^62^ algorithm for the occupied MOs, and the default parameter set, which corresponds to the localcc = 2024 and lcorthr = normal keywords, was used for the construction of subspaces. The DFT methods included several rungs from Jacob’s ladder of density functionals. Among the pure DFT functionals based on the generalized-gradient approximation (GGA), the Perdew–Burke–Ernzerhof (PBE) functional^63^ was used with Grimme’s D3 dispersion correction^64^ (PBE-D3). Of the hybrid-GGA functionals, where the exchange functional is mixed with the Hartree–Fock exchange, the PBE0 ^63,65^ approach was utilized with the D3 dispersion correction (PBE0-D3). For a double-hybrid functional, PBE0-2 ^66^ was selected, which combines the PBE correlation functional with MP2 correlation energy. The D3 corrections used the Becke and Johnson damping scheme.^67^ In addition, the semiempirical density functional tight-binding method GFN2-xTB (Geometry, Frequency, Noncovalent, eXtended TB)^68^ was also employed. DFT calculations utilized the default adaptive 590-point Lebedev grid^69^ along with the Log3 quadrature.^70^ Dunning’s correlation-consistent double- and triple-ζ AO basis sets (aug-cc-pVDZ, cc-pVTZ, aug-cc-pVTZ) were employed.^71,72^ The density-fitting technique was used for both the SCF and correlation calculations utilizing the corresponding auxiliary basis sets of Weigend.^73–75^ For closed-shell molecules, restricted orbitals were employed, while the unrestricted and restricted open-shell wave functions were used for molecules with open shells, except for GFN2-xTB calculations, where only a restricted open-shell formulation was utilized. In the case of open-shell LNO–CCSD­(T) calculations with the unrestricted reference wave function, quasi-restricted orbitals (QROs)^61,76^ were constructed. Note that our open-shell local correlation approaches are designed for restricted open-shell reference wave functions, thus the use of QROs allow computationally more tractable correlation calculations.

The Huzinaga-equation-based PbE, mechanical (ONIOM-ME) and electronic (ONIOM-EE) embedding variants of ONIOM, vacuum-embedding, and the multilevel local correlation (MLC) techniques were utilized in our assessment. The brief overview of the methods competing with the PbE method are presented in the Appendix. LNO–CCSD­(T), PBE0-2, and PBE0-D3 were used as high-level methods, thus the corresponding multilevel approaches included LNO–CCSD­(T)-in-PBE-D3, LNO–CCSD­(T)-in-PBE0-D3, PBE0-2-in-PBE-D3, PBE0-2-in-PBE0-D3, and PBE0-D3-in-PBE-D3. The ONIOM calculations were supplemented with the LNO–CCSD­(T)-in-GFN2-xTB, PBE0-2-in-GFN2-xTB, and PBE0-D3-in-GFN2-xTB techniques. When LNO–CCSD­(T) is the target method, the performance of the LNO–CCSD­(T)-in-LMP2 approach was also evaluated. In addition to embedding approaches based exclusively on ROHF (ROKS) and UHF (UKS) wave functions, ROKS-in-UKS (ROHF-in-UKS) and UKS-in-ROKS (UHF-in-ROKS) type embedding schemes were also tested in multilevel calculations. In the case of the Huzinaga embedding with UHF-in-UKS type calculations, the embedding potential was constructed as in ROHF-in-UKS type wave functions to ensure consistency between the embedding potential and the applied QROs in the correlation calculations. Note that this only affects LNO–CCSD­(T)-in-DFT computations, moreover, the PbE approach with PBE0-2 as high-level method evaluates the MP2 energy using the full virtual orbital MO set.

The Huzinaga-equation-based PbE approach employed the SPADE localization technique,^29^ and the MOs of the active subsystem were selected with the scheme that corresponds to SPADE. Note that the PbE method separates the molecules at the MO level, however, the predefined set of active atoms were used to calculate the subsystem D3 correction. MLC calculations utilized Boys–Foster localization, and the MOs of the active subsystem were selected based on their Mulliken-population on the atoms of the active subsystem. An MO was considered active if the latter quantity was grater than 0.3. The border atoms, which are active atom and environment atom pairs at the subsystem border, were selected manually for the ONIOM scheme. Hydrogen atoms were applied as link atoms, and they were positioned between the atoms of different layers, 1.08 Å away from the atom that belonged to the active subsystem. The ONIOM-EE calculation used the intrinsic atomic orbital (IAO) charges,^77^ except for the calculations with GFN2-xTB, where Mulliken charges were utilized. Charges assigned to environment border atoms were set to zero. The residual charge—calculated as the difference between the total point charges assigned to the active atoms and environment border atoms, and the integer charge of the active subsystem—was evenly distributed among the remaining point charges of the environment subsystem. In the case of vacuum embedding, the model systems were constructed in the same way as in the ONIOM method, by saturating the active subsystem with hydrogen atoms.

The geometries of the molecules used in the benchmark calculations were prepared in the following procedure: first, the two-dimensional structures were drawn in MolView,^78^ then the structures were converted into a three-dimensional form and edited with Molden.^79,80^ The structures were first relaxed with GFN2-xTB, then an additional relaxation was performed at the PBE0-D3/aug-cc-pVDZ level. Geometry optimization of the molecules with open-shell were performed using unrestricted wave function. The figures representing the subsystems of the test structures were prepared with MarvinJS.^81^

## 4 Results

To investigate the considered multilevel approaches, we selected four test systems—labeled with Roman numerals—that represent medium-sized molecules undergoing radical reactions in real-world systems, and which pose increasing challenges on multilevel methods due to the complexity of subsystem partitioning. The first test system (I) involves the chain growth of polyvinyl chloride (PVC), in which a PVC radical composed of five monomer units reacts with vinyl chloride to form a PVC radical with six monomer units. These are quasi one-dimensional molecules, where the electron density in the vicinity of the active radical is moderately polarized, and the molecular chains can be easily partitioned into active and environmental regions. The second test system (II) concerns the chain growth of poly­(methyl methacrylate) (PMMA), where a PMMA radical with three monomer units reacts with a methyl methacrylate molecule to produce a radical PMMA chain with four monomer units. Like the previous system, this one is also easily partitionable, although PMMA radicals are less linear. In the third test system (III), the thiol group of an alanine-cysteine-alanine (Ala-Cys-Ala) tripeptide reacts with a hydroxyl radical, resulting in the formation of a water molecule and a tripeptide containing a thiyl radical. This test represents a biological system, where the presence of peptide bonds makes it more challenging to divide the system into subsystems. The fourth test system (IV) models the singlet and triplet splitting of an N-heterocyclic carbene (NHC), where the conjugated electronic structure further complicates the partitioning of the system. The chemical structures of the test systems are shown in Figures 1, 4, 7, and 10, where the progressive expansion of the active subsystems for each multilevel method is also illustrated. This approach enables us to investigate the errors of multilevel methods as a function of active subsystem size.

**Figure 1:**
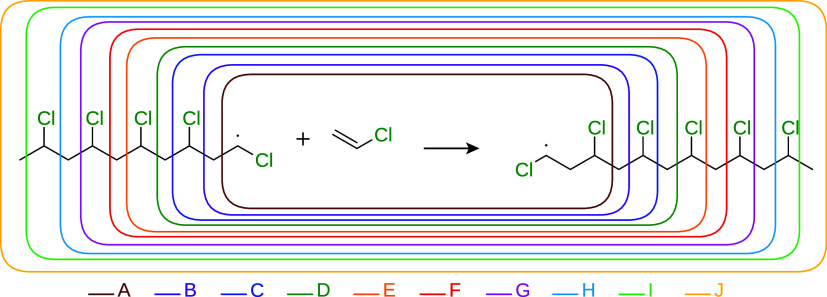
Test reaction I, where the chain growth of a PVC polymer is modeled. The active subsystems in the multilevel calculations are indicated by the rounded square areas in different colors. Each active subsystem is labeled by a capital letter, and the labels are color-coded to match the corresponding region.

**Figure 2:**
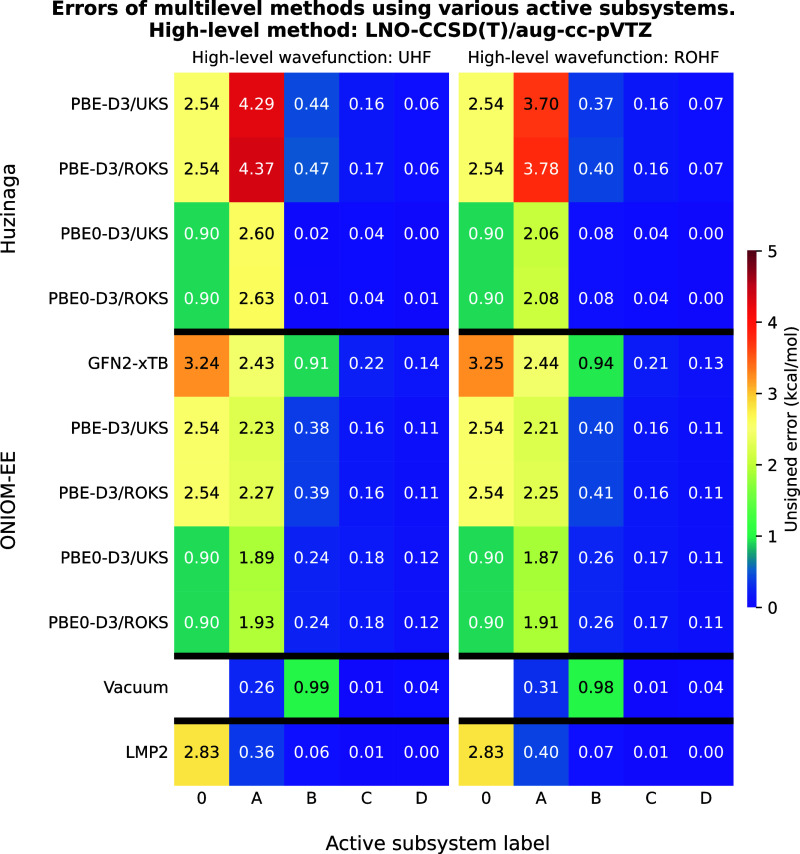
Unsigned errors of the multilevel methods as a function of the active subsystems for test reaction I. The meaning of the active subsystem labels is shown in Figure 1, moreover, label “0” denotes results of the low-level methods. LNO–CCSD­(T)/aug-cc-pVTZ was used as high-level method yielding a reaction energy of −30.08 kcal/mol with both UHF and ROHF reference wave functions. The leftmost column shows the type of the wave function Ansatz used for open-shell molecules and the theoretical method applied at the low-level. The techniques are grouped according to the multilevel approaches.

**Figure 3:**
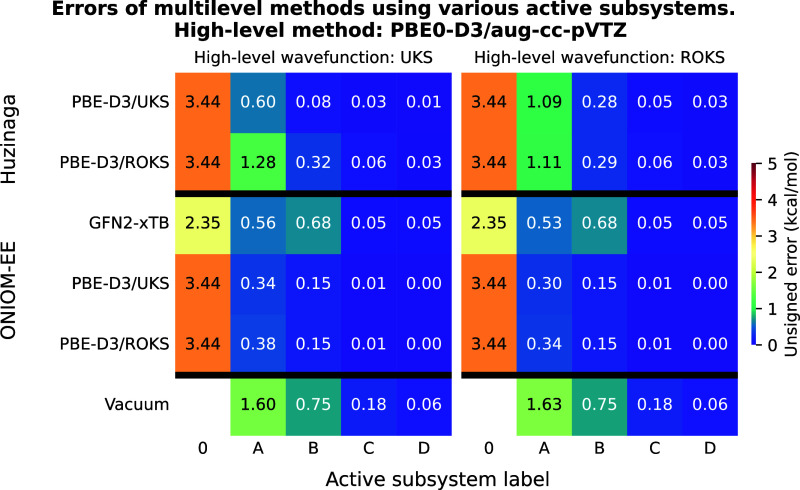
Unsigned errors of the multilevel methods as a function of the active subsystems for test reaction I. The meaning of the active subsystem labels is shown in Figure 1. PBE0-D3/aug-cc-pVTZ was used as high-level method yielding a reaction energy of −30.98 kcal/mol with both UKS and ROKS reference functions. For a detailed explanation of the axes, please refer to Figure 2.

**Figure 4:**
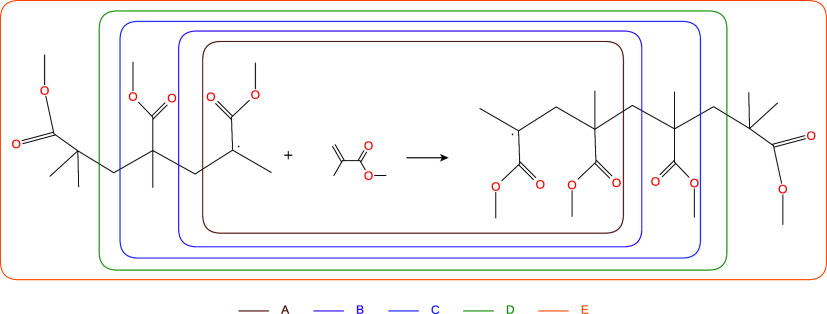
Test reaction II, where the chain growth of a PMMA polymer is modeled. The active subsystems in the multilevel calculations are indicated by the rounded square areas in different colors. Each active subsystem is labeled by a capital letter, and the labels are color-coded to match the corresponding region.

**Figure 5:**
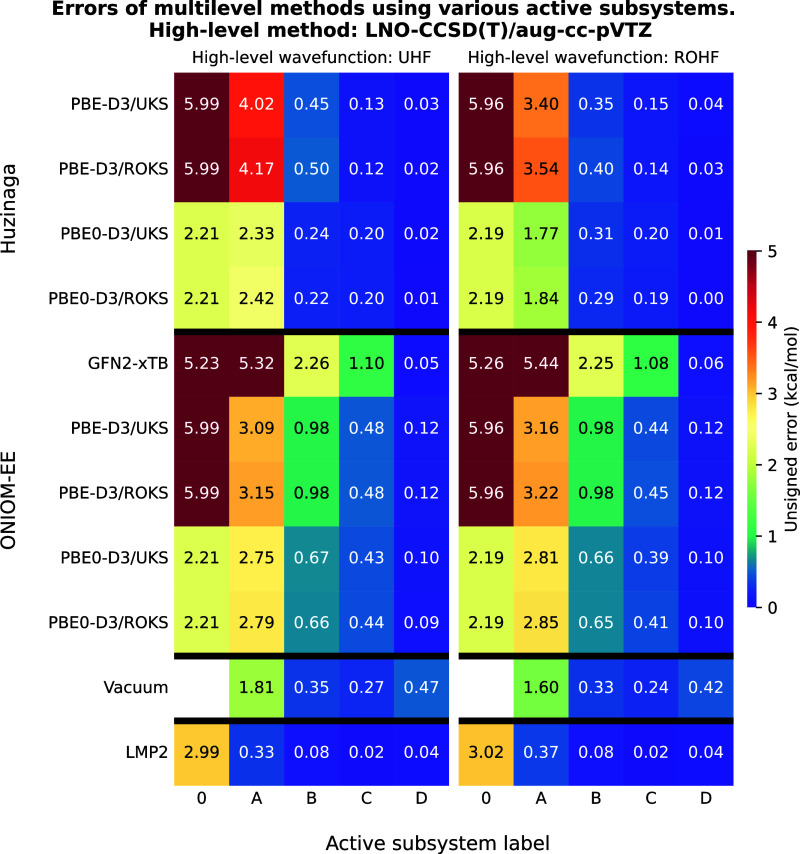
Unsigned errors of the multilevel methods as a function of the active subsystems for test reaction II. The meaning of the active subsystem labels is shown in Figure 4. LNO–CCSD­(T)/aug-cc-pVTZ was used as high-level method yielding a reaction energy of −24.79 and −24.77 kcal/mol with UHF and ROHF reference wave functions, respectively. For a detailed explanation of the axes, please refer to Figure 2.

**Figure 6:**
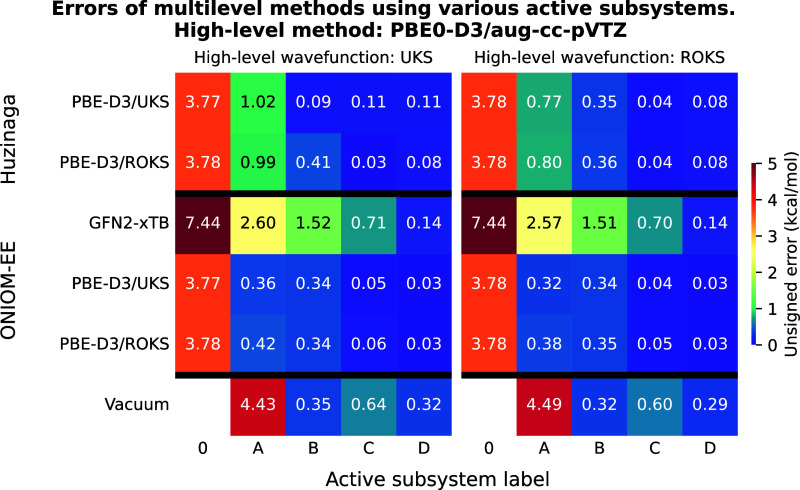
Unsigned errors of the multilevel methods as a function of the active subsystems for test reaction II. The meaning of the active subsystem labels is shown in Figure 4. PBE0-D3/aug-cc-pVTZ was used as high-level method yielding a reaction energy of −22.58 and −22.59 kcal/mol with UKS and ROKS reference functions, respectively. For a detailed explanation of the axes, please refer to Figure 2.

**Figure 7:**
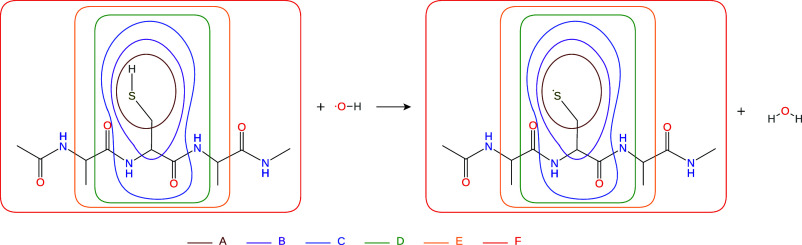
Test reaction III, where the hydroxyl-radical reacts with the thiol group of the Ala-Cys-Ala tripeptide. The active subsystems in the multilevel calculations are indicated by the areas in different colors. Each active subsystem is labeled by a capital letter, and the labels are color-coded to match the corresponding region. Note that the hydroxyl radical and the water molecule are always part of the active subsystem despite being shown otherwise in the figure.

To compare the multilevel approaches, we employed different low-level methods in combination with various high-level methods, including LNO–CCSD­(T), PBE0-2, and PBE0-D3, to calculate reaction energies. Our evaluation focuses on two main aspects: accuracy and computational cost, while also considering the robustness of each technique. We also examined the effects of using different reference wave functions for the various high- and low-level method combinations. In the main text, we present only the results obtained with LNO–CCSD­(T) and PBE0-D3 as the high-level methods since the results using PBE0-2 exhibits similar trends. The ONIOM-ME and ONIOM-EE methods also performed similarly, therefore, the results of the calculations using ONIOM-ME and PBE0-2 are provided in the Electronic Supporting Information (ESI). Note that we present only those multilevel calculations in which the choice of the active subsystem resulted in significant errors; the full set of results is available in the ESI.

### 4.1 Test Reaction I: Polyvinyl Chloride Chain Growth

The results of test reaction I obtained using the LNO–CCSD­(T) method are shown in Figure 2. The most accurate approach is LNO–CCSD­(T)-in-LMP2 as it requires only the carbon atom bearing the radical and its associated chloride atom (subsystem “A”) to be treated at the high-level in order to achieve an error below 1 kcal/mol. In contrast, the PbE and ONIOM techniques require one additional functional group (subsystem “B”) to reach comparable accuracy, while the use of GFN2-xTB as a low-level method still results in an error close to 1 kcal/mol even at this larger subsystem size. With vacuum embedding, accurate reaction energies can already be obtained using subsystem “A”, however, increasing the size of the active subsystem leads to a significant increase in the error (subsystem “B”). There is no notable difference between results obtained using ROHF (ROKS) and UHF (UKS) reference wave functions. For a given subsystem, the ROHF-in-UKS (UHF-in-UKS) approach seems marginally more accurate than ROHF-in-ROKS (UHF-in-ROKS). It is worth noting that using hybrid-GGA instead of GGA as a low-level technique in ONIOM and PbE calculations yields slightly improved accuracy, but accurate reaction energies still require the larger “B” subsystem—meaning these methods remain uncompetitive with LNO–CCSD­(T)-in-LMP2. Thus, the only robust and cost-efficient alternatives are the GGA-based PbE and ONIOM methods, with both offering similar accuracy. In its original formulation, PbE requires the use of the supersystem AO basis set for both the high- and low-level calculations, whereas ONIOM can leverage only the subsystem AO basis set at the high level. However, the adaptive AO basis set trunction^36^ allows more efficient calculations in PbE, thus it is not unclear which method is more favorable.

The results of the calculations using PBE0-D3 as the high-level method are shown in Figure 3. Among the considered techniques, the ONIOM PBE0-D3-in-PBE-D3 (ROKS-in-ROKS) approach offers the best accuracy-over-cost ratio, yielding accurate results already with subsystem “A”, and performing well with other wave function combinations as well. The PbE with ROKS reference only provides sufficient accuracy when subsystem “B” is the high-level region, whereas the UKS-in-UKS approach significantly improves the accuracy of the reaction energy. Comparing the different wave function combinations for a given subsystem calculation, we observe the same trend as for the LNO–CCSD­(T) example: ROKS-in-UKS (UKS-in-UKS) yields marginally more accurate results than ROKS-in-ROKS (UKS-in-ROKS). Using ONIOM in combination with GFN2-xTB also results in quite accurate predictions, although increasing the active subsystem from “A” to “B” slightly increases the error. The least accurate technique is vacuum embedding, which produces an error of 0.75 kcal/mol even when applied to the active subsystem “B”.

For methods employing PBE0-2, it can be stated that the convergence of errors with respect to the active subsystem size is similar to what was observed in the LNO–CCSD­(T) calculations, however, the errors are 1–2 kcal/mol lower when subsystem “A” is treated at the high-level. Finally, we would like to note concerning the ONIOM-EE method that the initial WFT-in-DFT and DFT-in-DFT calculations employed Mulliken point charges to represent the environment. However, these charges assigned unrealistically high values to the chloride atoms, leading to instability in the SCF calculations for the subsystems. Since high charge densities can easily occur in larger applications, we recommend exercising caution when interpreting results from ONIOM-EE methods based on Mulliken charges.

### 4.2 Test Reaction II: Poly­(methyl methacrylate) Chain Growth

The results of the multilevel methods using LNO–CCSD­(T) for the PMMA test system are shown in Figure 5. As in the previous example, LNO–CCSD­(T)-in-LMP2 provides the most accurate results, achieving high accuracy even with the smallest active subsystem. A key difference from the results of the previous test is that the second best method here is clearly the PbE approach as the ONIOM method still yields an error of around 1 kcal/mol even with active subsystem “B”. When ONIOM is combined with GFN2-xTB, the error reduction with increasing active subsystem size is even slower. Using vacuum embedding with subsystem “B” results in smaller errors than PbE, but surprisingly, the error increases again for subsystem “D”. Overall, in this example, the Huzinaga-based LNO–CCSD­(T)-in-PBE-D3 appears to be the most effective competitor to LNO–CCSD­(T)-in-LMP2—it is more cost-efficient, more accurate than ONIOM, and allows for systematic improvement compared to vacuum embedding.

The results of the calculations targeting PBE0-D3 reaction energies are shown in Figure 6, and they exhibit characteristics very similar to those observed for the previous example. The lowest errors are found for PBE0-D3-in-PBE-D3 calculations, where ONIOM clearly outperforms PbE. The oscillatory error behavior previously seen with vacuum embedding is also observed here, and the ONIOM approach using GFN2-xTB shows the slowest convergence of error with respect to the size of the active subsystem. As for the PBE0-2-based calculations, their overall behavior again mirrors that of the LNO–CCSD­(T) results, however, when using the “B” active subsystem, ONIOM achieves accuracy comparable to that of the PbE approach.

### 4.3 Test Reaction III: Formation of Thiyl Radical

The results of the local correlation calculations for test system III are presented in Figure 8. According to the data, the most accurate method is, once again, the LNO–CCSD­(T)-in-LMP2 approach. All other techniques fall significantly short in accuracy—particularly the GGA-based PbE method. The ONIOM approach using GFN2-xTB as a low-level technique proves to be accurate with small active subsystems, but the error trends become inconsistent as the subsystem size increases. The most efficient method is likely vacuum embedding, although it lacks robustness. A notable difference compared to previous examples is that the use of hybrid-GGA functionals leads to a marked improvement in accuracy for both the PbE and ONIOM techniques. This example also demonstrates the applicability of ROHF-in-UKS-type multilevel techniques as the ROKS equations could not be solved using the PBE functional. This is likely due to the self-interaction error inherent to GGAs, which can increase the energy level of the highest occupied MO (HOMO) to the level of the lowest unoccupied MO (LUMO), leading to a vanishing HOMO–LUMO gap and SCF instabilities. The results should also be interpreted in light of how subsystems were defined and how multilevel methods describe the interactions between the active and environment subsystems. Based on the atoms assigned to the singly occupied MO of the ROHF wave function by the Boughton–Pulay algorithm,^83^ the accurate description of this MO requires not only the AOs of the sulfur atom and its attached methylene group, but also the nearby AOs centered on the atoms of the peptide bond and the atoms of the Ala side chain. In this context, the relatively accurate ONIOM reaction energies are likely the result of fortunate error cancellation, and this also helps explain why the Huzinaga-equation-based LNO–CCSD­(T)-in-PBE0 requires at least subsystem “D” to be treated at high-level to yield accurate reaction energies. It is also worth noting that the PbE technique is significantly more robust than the ONIOM method as it can partition subsystems even across peptide bonds.

**Figure 8:**
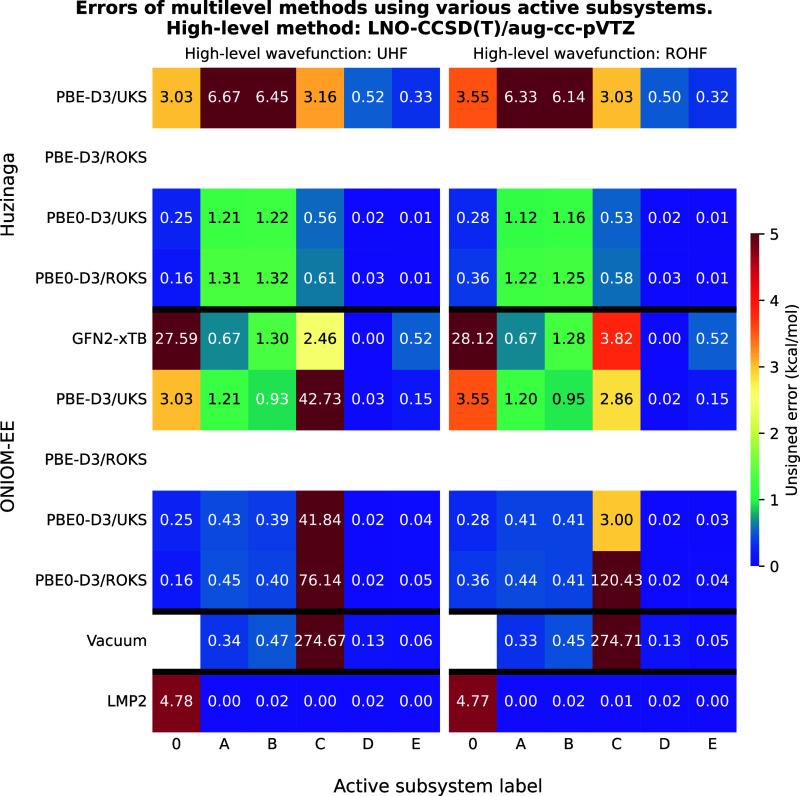
Unsigned errors of the multilevel methods as a function of the active subsystems for test reaction III. The meaning of the active subsystem labels is shown in Figure 7. LNO–CCSD­(T)/aug-cc-pVTZ was used as high-level method yielding a reaction energy of −34.14 and −33.62 kcal/mol with UHF and ROHF reference wave functions, respectively. For a detailed explanation of the axes, please refer to Figure 2. The empty rows indicate that low-level methods did not yield SCF solutions for the doublet state.

The results in which PBE0-D3 is used as the high-level method are shown in Figure 9. In the PBE0-D3-in-PBE-D3 case, the ONIOM and PbE approaches yield much more similar results. For the ROKS-in-UKS wave function, ONIOM proves to be the more accurate method, although it is unable to separate across the peptide bond. In contrast, for the UKS-in-UKS wave function, the PbE method is clearly more efficient as it only requires subsystem “A” to be treated at the high-level. Vacuum embedding with a ROKS wave function appears to be competitive, but the method lacks robustness, and increasing the size of the active subsystem does not necessarily reduce the error. With the ONIOM approach using the GFN2-xTB method, a similar trend is observed—albeit with larger errors—, however, the error increases substantially when the largest embedded subsystem is used.

**Figure 9:**
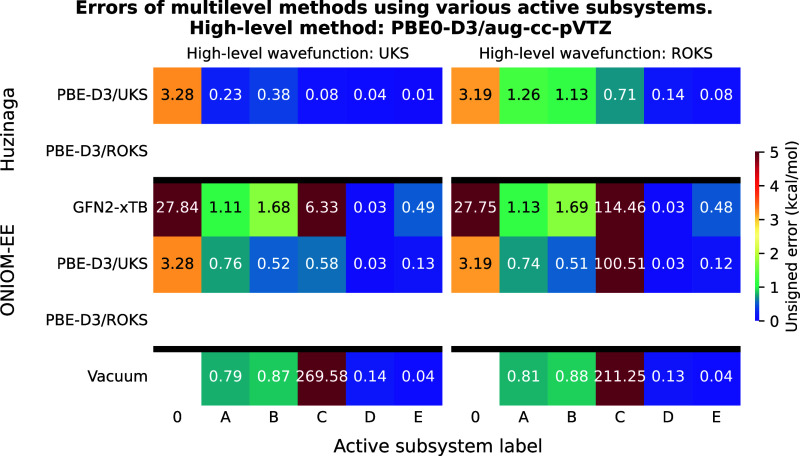
Unsigned errors of the multilevel methods as a function of the active subsystems for test reaction III. The meaning of the active subsystem labels is shown in Figure 7. PBE0-D3/aug-cc-pVTZ was used as high-level method yielding a reaction energy of −33.89 and −33.98 kcal/mol with UKS and ROKS reference functions, respectively. For a detailed explanation of the axes, please refer to Figure 2. The empty rows indicate that low-level methods did not yield SCF solutions for the doublet state.

**Figure 10:**
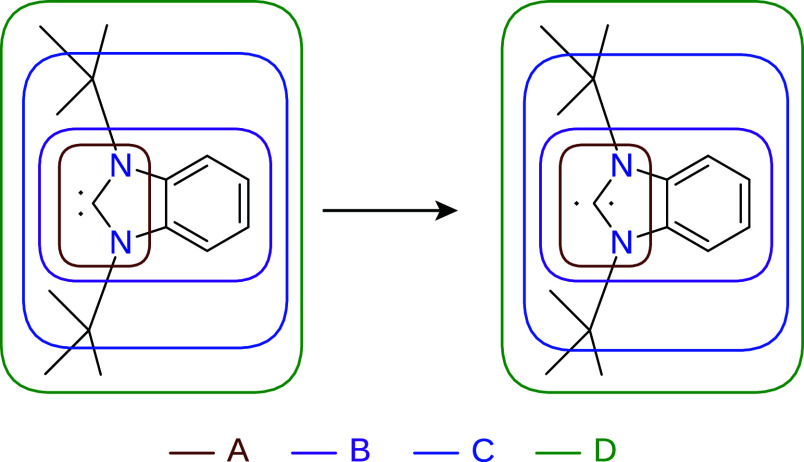
Test reaction IV, where the singlet–triplet splitting of an N-heterocyclic carbene is modeled. The vertically aligned dots symbol the singlet state, and the horizontally aligned dots denote the triplet state. The active subsystems in the multilevel calculations are indicated by the areas in different colors. Each active subsystem is labeled by a capital letter, and the labels are color-coded to match the corresponding region.

It is also worth briefly discussing the results obtained with PBE0-2. In the UKS-in-UKS (or UKS-in-ROKS) cases, the trends tend to follow those observed with PBE0-D3, whereas with the ROKS-in-UKS (or ROKS-in-ROKS) wave functions, the behavior is more reminiscent of what was seen for LNO–CCSD­(T). Notably, the PbE-based PBE0-2-in-PBE-D3 (UKS-in-UKS) approach clearly outperforms the ONIOM method—even when compared to ONIOM calculations using PBE0-2-in-PBE0-D3 (UKS-in-UKS). These ONIOM examples also exhibit an unusual pattern: errors remain small for smaller active subsystems but can increase by up to 1 kcal/mol when the “E” partitioning is used. This suggests that the error cancellation in ONIOM methods may not apply consistently across subsystem sizes.

### 4.4 Test Reaction IV: Singlet–Triplet Splitting of a Carbene Molecule

The LNO–CCSD­(T) results for test reaction IV are shown in Figure 11. The data once again indicate that LNO–CCSD­(T)-in-LMP2 is the most reliable method, although in this case, accurate results require the use of active subsystem “B”. Neither ONIOM nor PbE yields accurate singlet–triplet gaps when smaller active subsystems are used—for instance, in the case of subsystems “A” and “B”—in this example. Only the LNO–CCSD­(T)-in-PBE0-D3 (UHF/ROHF-in-UKS) calculation using ONIOM results in an error below 1 kcal/mol, but the error increases somewhat when subsystem “C” is used as active. Vacuum embedding proves inaccurate even with subsystem “C”, while ONIOM with GFN2-xTB performs better than with PBE-D3. As shown in the previous example, no SCF solution was found for the PBE method with the ROKS approach here as well, and the self-interaction error causes SCF instabilities even with the PBE0 functional for this Ansatz. Notably, increasing the exact HF exchange ratio (as in the case of PBE0-2) stabilizes the triplet state even with ROKS. Altogether, these are further examples illustrating that (i) hybrid GGAs play a more significant role for open-shell systems at the low level of theory, and (ii) ROHF-in-UKS multilayer approaches can be particularly useful in practice. It is also worth noting in the context of error convergence with respect to the active subsystem that the significant inaccuracy observed when separating aromatic systems using the PbE method with a local correlation approach as high-level method is consistent with earlier results obtained for closed-shell molecules.^13^

**Figure 11:**
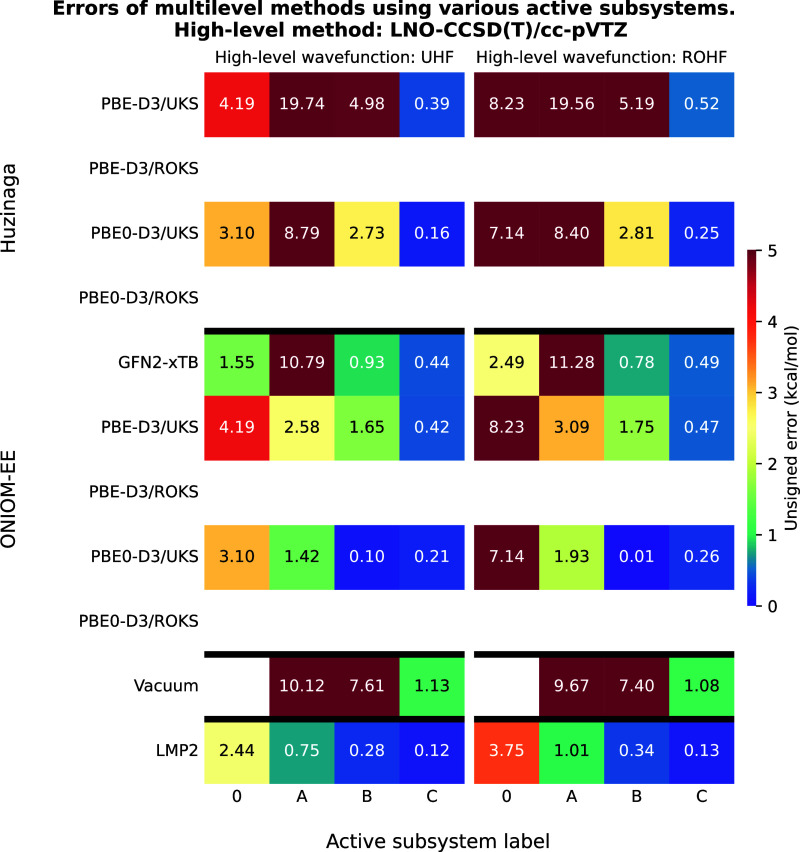
Unsigned errors of the multilevel methods as a function of the active subsystems for test reaction IV. The meaning of the active subsystem labels is shown in Figure 10. LNO–CCSD­(T)/cc-pVTZ was used as high-level method yielding a singlet–triplet splitting of 77.94 and 81.99 kcal/mol with UHF and ROHF reference wave functions, respectively. For a detailed explanation of the axes, please refer to Figure 2. The empty rows indicate that low-level methods did not yield SCF solutions for the triplet state.

The results targeting the PBE0-D3 level for test system IV are shown in Figure 12. In this case, the PbE method is clearly more favorable. For PBE0-D3-in-PBE-D3 (UKS-in-UKS), the errors with PbE and ONIOM are similar if subsystem “A” is used as active, the error decreases with PbE using the “B” active subsystem, whereas the error increases with ONIOM. Interestingly, this trend continues even if subsystem “C” is used as active with a greater change in errors. Applying ONIOM with GFN2-xTB yields even better results than with PBE for the larger active subsystems, with the error decreasing consistently. In contrast, vacuum embedding fails to provide accurate singlet–triplet gaps with any of the active subsystems.

**Figure 12:**
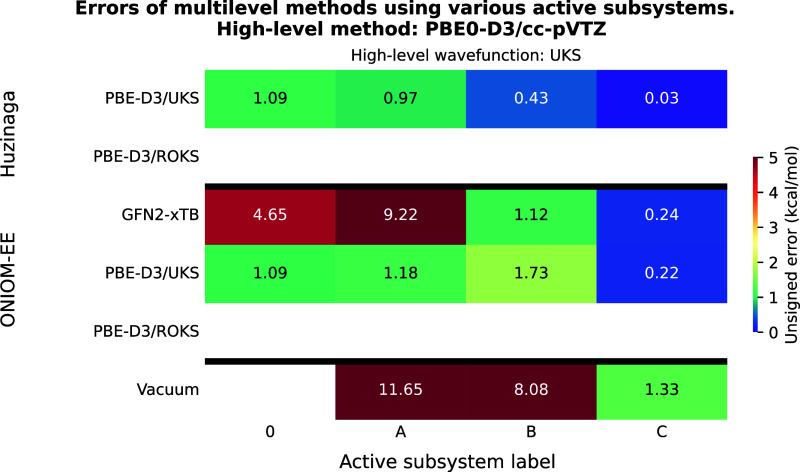
Unsigned errors of the multilevel methods as a function of the active subsystems for test reaction IV. The meaning of the active subsystem labels is shown in Figure 7. PBE0-D3/cc-pVTZ was used as high-level method yielding a singlet–triplet splitting of 74.85 kcal/mol with UKS reference functions. The target method does not yield an SCF solution with the ROKS Ansatz for the triplet state. For a detailed explanation of the axes, please refer to Figure 2. The empty rows indicate that low-level methods did not yield SCF solutions for the triplet state.

For the PBE0-2 results, a familiar trend can again be observed: the UKS-in-UKS results resemble those seen with PBE0-D3, while the accuracy of the ROKS-in-UKS calculations aligns more closely with what was observed for LNO–CCSD­(T). One possible explanation for the differing error characteristics observed with ROKS and UKS wave functions is related to the orbital localization procedure. In the RO Ansatz, only orbitals within the same shell are mixed during localization, which effectively leaves the open-shell orbitals delocalized. In contrast, using the UKS wave function allows mixing between orbitals of the same spin, enabling more localized orbitals to form. As a result, accurately describing the system requires a smaller active subsystem when using UKS.

## 5 Conclusions

In this study, we presented the extension of the Huzinaga-equation-based PbE method to open-shell systems. While the formulation of the unrestricted PbE approach follows relatively naturally from its closed-shell counterpart, the restricted variant is less straightforward. Our work builds on earlier approaches developed by Roothaan, Filatov, and Shaik,^48–50^ which offer a computationally efficient alternative to unrestricted open-shell methods, while also avoiding spin contamination. We implemented the PbE framework to support both the restricted and unrestricted wave function Ansatz as well as their combinations. This design choice provides flexibility: unrestricted-in-restricted (e.g., UKS/UHF-in-ROKS) embedding can offer computational savings at the supersystem level, while restricted-in-unrestricted (e.g., ROKS/ROHF-in-UKS) embedding can help bypass SCF convergence issues sometimes encountered with restricted open-shell methods.

We assessed the performance of the PbE approach using a range of WFT and DFT methods. As high-level methods, we selected locally approximated CCSD­(T), double-hybrid DFT, and hybrid-GGA DFT, while GGA and hybrid-GGA functionals were used at the low-level. The performance of the embedding method was benchmarked against various focused multilevel strategies, including local correlation-based techniques, ONIOM-ME, ONIOM-EE, and vacuum embedding. The ONIOM framework further allowed us to explore the applicability of the semiempirical GFN2-xTB method at the low-level. To evaluate the multilevel methods, we designed four test reactions that are representative of realistic applications. Each involves medium-sized molecules to keep the computations tractable, while presenting an increasingly challenging task in terms of subsystem partitioning. The selected reactions include the chain growth of PVC and PMMA, the formation of a thiyl radical in a tripeptide containing cysteine, and the singlet–triplet splitting of an N-heterocyclic carbene.

The results of the multilevel approaches targeting LNO–CCSD­(T) indicate that the most reliable method is LNO–CCSD­(T)-in-LMP2, which utilizes focused local approximations. In comparison, PbE and ONIOM approaches based on hybrid-GGA functionals are not competitive in terms of efficiency. For LNO–CCSD­(T)-in-GGA calculations, it is less clear which of the two—PbE or ONIOM—is preferable. While ONIOM can sometimes yield more accurate results and is computationally cheaper, the PbE approach is more robust and, based on our examples, its accuracy can be systematically improved. When ONIOM is used with the GFN2-xTB method at the low-level, achieving accurate results generally requires the use of a somewhat larger active subsystem, but it seems to be a promising alternative of the QM/MM approaches for medium- and large-scale applications because there is no need for complicated parametrization. As for vacuum embedding, it often delivers ONIOM-level accuracy, but the error does not consistently decrease with increasing subsystem size. For multilevel calculations where the high-level method was a hybrid-GGA or double-hybrid DFT, trends similar to the previously discussed LNO–CCSD­(T)-targeting schemes were observed, however, the errors were smaller, and a smaller active subsystem was sufficient to achieve accurate results. Note that this difference between the error-characteristics of WFT-in-WFT and DFT-in-DFT calculations is in line with previous findings,^82^ where the smaller errors observed in DFT-in-DFT were attributed to error cancellation effects.

It is also worth noting that UKS-in-ROKS-type calculations using the ONIOM and PbE methods yield results that are only a few tenths of a kcal/mol less accurate than those from pure UKS-in-UKS embeddings. In contrast, ROKS/ROHF-in-UKS tends to be somewhat more accurate than approaches relying solely on the restricted wave function. In two examples, we demonstrated that ROHF/ROKS-in-UKS can serve as a valuable alternative when the SCF procedure for the full system employing the ROKS wave function encounters convergence issues.

The considered methods—vacuum embedding, ONIOM-ME, ONIOM-EE, PbE, and MLC—reflect a progressive refinement in how subsystem interactions are modeled. Despite this hierarchy, accurate results can often be achieved even with the simpler approaches. For vacuum embedding, ONIOM-ME, and ONIOM-EE, we suggested that this may be due to fortunate error cancellation. However, such compensation is not guaranteed as the error does not necessarily decrease with the size of the active subsystem. A similar behavior may occasionally arise in UKS-in-UKS-type PbE embeddings, however, in the examples presented, such deviations were rare, with the error increasing only marginally—by a few tenths of a kcal/mol. Nevertheless, this conjecture requires further investigation on systems with more extended three-dimension structures.

Additionally, the considerable differences in accuracy observed between the MLC and PbE approaches merit further studies. Our results indicate that PbE can yield reaction energies comparable to those obtained with MLC, provided that the occupied MOs of the extended domain associated with the reactive center are also treated at the high level. This finding suggests that, as with closed-shell systems, the most efficient strategy for open-shell cases is possibly a three-layer approach that utilize the LNO–CCSD­(T)-in-LMP2-in-GGA type embedding.

Taken together, these results demonstrate that the tools presented here are effective for the efficient modeling of open-shell systems. Nevertheless, further testing on larger and more complex molecules is needed in order to formulate more informed recommendations for efficient calculations in large-scale applications.

## Supplementary Material





## Appendix

### Detailed Description of the Projection-Based Embedding Using the Spin-Unrestricted Formalism

To evaluate the composite energy of eq 13, the first step is to minimize *E*_2,UKS_[**D** ^α,AB^, **D** ^β,AB^] under the constraint that the spin orbitals are orthonormal to same spin orbitals. However, orbitals with different spins are not mutually orthogonal, which is the source of the spin contamination anomaly of the spin-unrestricted approaches. Let us define the following Lagrangian

$$L_{2,\mathrm{UKS}}^{\mathrm{AB}}=E_{2,\mathrm{UKS}}[D^{\alpha ,\mathrm{AB}},\;D^{\beta ,\mathrm{AB}}]-\sum_{\sigma }\mathrm{Tr}\{((C^{\sigma }{)}^{T}SC^{\sigma }-1)\epsilon_{2,\mathrm{UKS}}^{\sigma }\}$$
80

where **ϵ**_2,UKS_^σ^ are the matrices of the Lagrange multipliers. A pseudo-eigenvalue equation can be obtained for the MO coefficients if the above equation is minimized with respect to **C**_μ*i*_^α^ and **C**_μ*i*_^β^:

$$F_{2,\mathrm{UKS}}^{\sigma }[D^{\alpha ,\mathrm{AB}},\;D^{\beta ,\mathrm{AB}}]C_{i}^{\sigma }=\sum_{j}SC_{j}^{\sigma }(\epsilon_{2,\mathrm{UKS}}^{\sigma }{)}_{ji}$$
81

where **F**_2,UKS_^σ^ is the Fock (Kohn–Sham) matrix:

$$F_{2,\mathrm{UKS}}^{\sigma }[D^{\alpha ,\mathrm{AB}},\;D^{\beta ,\mathrm{AB}}]=h_{\mathrm{core}}+J[D^{\alpha ,\mathrm{AB}}+D^{\beta ,\mathrm{AB}}]-a_{\mathrm{HF},2}K[D^{\sigma ,\mathrm{AB}}]+(1-a_{\mathrm{HF},2})V_{\mathrm{xc},2}^{\sigma }[D^{\alpha ,\mathrm{AB}},\;D^{\beta ,\mathrm{AB}}]$$
82

Spin-orbitals that minimize the energy can be obtained with the iterative diagonalization of **F**_2, UKS_^σ^[**D** ^α,AB^, **D** ^β,AB^], starting from a reasonable guess, until self-consistency is reached. Subsequently, *E*_2_[**D** ^α,AB^, **D** ^β,AB^] is evaluated, which is followed by the separate localization of the α and β occupied spin orbitals as

$$L_{j}^{\sigma }=\underset{i}{\sum }C_{i}^{\sigma }W_{ij}^{\sigma }$$
83

where **W** ^σ^ is the spin-dependent unitary matrix that transforms the canonical occupied MOs to localized ones, thus **L** ^σ^ are the MO coefficient matrices of the latter. Next, the localized MOs are assigned to subsystems:

$$L^{\sigma }=L^{\sigma ,A}\cup L^{\sigma ,B}$$
84

where **L** ^σ,A^ and **L** ^σ,B^ are the active and environment occupied blocks of **L** ^σ^, respectively. The assignation is typically based on the Mulliken population of the orbitals on a predefined set of atoms that are labeled as active,^13,24^ or the SPADE partitioning scheme.^29^ After the subsystems are defined, the subsystem density matrices of the active and environment regions are obtained using

$$\begin{array}{cc} D^{\sigma ,S}=L^{\sigma ,S}(L^{\sigma ,S}{)}^{T} & S\in \{A,B\} \end{array}$$
85

followed by the computation of the low-level subsystem Fockian, **F**_2, UKS_^σ^[**D** ^α,A^, **D** ^β,A^], the corresponding energy, *E*_2,UKS_[**D** ^α,A^, **D** ^β,A^], and a part of the correction term, $\underset{\sigma }{\sum }\mathrm{Tr}\left\{-D^{\sigma ,A}\frac{\partial E_{12,\mathrm{UKS}}}{\partial D^{\sigma ,A}}\right\}$ . The algorithm continues with the reoptimization of the orbitals of the embedded subsystem using a high-level functional in the field of the embedding potential under the constraint that the MOs of subsystem A are orthonormal to any other orbital of the same spin. The functional with these constraints can be defined with the following Lagrangian

$${\overset{\sim }{L}}_{\mathrm{UKS}}^{A}=E_{\mathrm{PbE},\mathrm{UKS}}-\underset{\sigma }{\sum }\{\mathrm{Tr}(\{({\overset{\sim }{C}}^{\sigma ,A}{)}^{T}S{\overset{\sim }{C}}^{\sigma ,A}-1\}{\overset{\sim }{\epsilon }}_{\mathrm{UKS}}^{\sigma })+\mathrm{Tr}(\{({\overset{\sim }{C}}^{\sigma ,A}{)}^{T}SL^{\sigma ,B}\}{\overset{\sim }{\eta }}_{\mathrm{UKS}}^{\sigma })\}$$
86

where the second term on the right side of the equation expresses the intra-subsystem orthonormality, while the last term ensures the inter-subsystem orthonormality, with ${\overset{\sim }{\epsilon }}_{\mathrm{UKS}}^{\sigma }$ and ${\overset{\sim }{\eta }}_{\mathrm{UKS}}^{\sigma }$ as the corresponding multipliers. Minimizing the Lagrangian of eq 86 with respect to **C̃**_μ*i*_^α^ and **C̃**_μ*i*_^β^, where *i* ∈A, results in the following equations

$$2{\overset{\sim }{F}}_{\mathrm{UKS}}^{\sigma }[{\overset{\sim }{D}}^{\alpha ,A},\;{\overset{\sim }{D}}^{\beta ,A}]{\overset{\sim }{C}}_{i}^{\sigma ,A}=2\underset{j}{\sum }S{\overset{\sim }{C}}_{j}^{\sigma ,A}({\overset{\sim }{\epsilon }}_{\mathrm{UKS}}^{\sigma }{)}_{ji}+\underset{j}{\sum }SL_{j}^{\sigma ,B}({\overset{\sim }{\eta }}_{\mathrm{UKS}}^{\sigma }{)}_{ji}$$
87

where **F̃**_UKS_^σ^ is the embedded Fock matrix:

$${\tilde{F}}_{\mathrm{UKS}}^{\sigma }[{\tilde{D}}^{\alpha ,A},\;{\tilde{D}}^{\beta ,A}]=F_{2,\mathrm{UKS}}^{\sigma }[D^{\alpha ,\mathrm{AB}},\;D^{\beta ,\mathrm{AB}}]-F_{2,\mathrm{UKS}}^{\sigma }[D^{\alpha ,A},\;D^{\beta ,A}]+F_{1,\mathrm{UKS}}^{\sigma }[{\tilde{D}}^{\alpha ,A},\;{\tilde{D}}^{\beta ,A}]$$
88

The embedded Fock matrix also depends on **D** ^α,AB^, **D** ^β,AB^, **D** ^α,A^, and **D** ^β,A^, but this dependency will be omitted in the notation. eq 88 can be recast in order to get a more practical formula:

$${\tilde{F}}_{\mathrm{UKS}}^{\sigma }[{\tilde{D}}^{\alpha ,A},\;{\tilde{D}}^{\beta ,A}]=h_{\mathrm{core},\mathrm{UKS}}^{\sigma ,A‐\mathrm{in}‐B}+J[{\tilde{D}}^{\sigma ,A}+{\tilde{D}}^{\beta ,A}]-a_{\mathrm{HF},1}K[{\tilde{D}}^{\sigma ,A}]+(1-a_{\mathrm{HF},1})V_{\mathrm{xc},1}^{\sigma }[{\tilde{D}}^{\alpha ,A},\;{\tilde{D}}^{\beta ,A}]$$
89

where the spin-dependent embedded core-Hamiltonian, **h**_core, UKS_^σ, A‑in‑B^, can be written as the sum of the core-Hamiltonian and the embedding potential:

$$h_{\mathrm{core},\mathrm{UKS}}^{A‐\mathrm{in}‐B,\sigma }=h_{\mathrm{core}}+F_{2,\mathrm{UKS}}^{\sigma }[D^{\alpha ,\mathrm{AB}},\;D^{\beta ,\mathrm{AB}}]-F_{2,\mathrm{UKS}}^{\sigma }[D^{\alpha ,A},\;D^{\beta ,A}]$$
90

which can replace **h**_core_ in the subsequent calculations. Note that the iterative diagonalization of **F̃** would produce spin orbitals that overlap with the orbitals of subsystem B, and this nonorthogonality would require the treatment of the nonadditive kinetic energy. Instead, let us express the off-diagonal multipliers as

$$2(L_{j}^{\sigma ,B}{)}^{T}{\tilde{F}}_{\mathrm{UKS}}^{\sigma }[{\tilde{D}}^{\alpha ,A},\;{\tilde{D}}^{\beta ,A}]{\tilde{C}}_{i}^{\sigma ,A}=({\tilde{\eta }}_{\mathrm{UKS}}{)}_{ji}$$
91

where **L** ^σ,B^ is not an eigenfunction of **F̃**_UKS_^σ^, thus ${\overset{\sim }{\eta }}_{\mathrm{UKS}}$ is nondiagonal. Let us introduce the following Hermitian operator that couples the orbitals of the active subsystem with the environment MOs:

$$2\{{\overset{\sim }{F}}_{\mathrm{UKS}}^{\sigma }[{\overset{\sim }{D}}^{\alpha ,A},\;{\overset{\sim }{D}}^{\beta ,A}]R^{\sigma ,B}S+SR^{\sigma ,B}{\overset{\sim }{F}}_{\mathrm{UKS}}^{\sigma }[{\overset{\sim }{D}}^{\alpha ,A},\;{\overset{\sim }{D}}^{\beta ,A}]\}{\overset{\sim }{C}}_{i}^{\sigma ,A}=\underset{j}{\sum }SL_{j}^{\sigma ,A}({\overset{\sim }{\eta }}_{\mathrm{UKS}}^{\sigma }{)}_{ji}$$
92

The active-environment block of eq 92 of the operator in the MO basis has the same matrix elements as eq 91, and if eq 92 is subtracted from eq 87, we get the spin-dependent Huzinaga equation

$${\overset{\sim }{H}}_{\mathrm{UKS}}^{\sigma }[{\overset{\sim }{D}}^{\alpha ,A},\;{\overset{\sim }{D}}^{\beta ,A}]{\overset{\sim }{C}}_{i}^{\sigma ,A}=\underset{j}{\sum }S{\overset{\sim }{C}}_{j}^{\sigma ,A}({\overset{\sim }{\epsilon }}_{\mathrm{UKS}}^{\sigma }{)}_{ji}$$
93

where the spin-dependent Huzinaga operator is

$${\tilde{H}}_{\mathrm{UKS}}^{\sigma }[{\tilde{D}}^{\alpha ,A},\;{\tilde{D}}^{\beta ,A}]={\tilde{F}}_{\mathrm{UKS}}^{\sigma }[{\tilde{D}}^{\alpha ,A},\;{\tilde{D}}^{\beta ,A}]-{\tilde{F}}_{\mathrm{UKS}}^{\sigma }[{\tilde{D}}^{\alpha ,A},\;{\tilde{D}}^{\beta ,A}]R^{\sigma ,B}S-SR^{\sigma ,B}{\tilde{F}}_{\mathrm{UKS}}^{\sigma }[{\tilde{D}}^{\alpha ,A},\;{\tilde{D}}^{\beta ,A}]$$
94

**H̃**_UKS_^σ^ has all the occupied orbitals of the corresponding spin as its eigenfunction, including the occupied, localized orbitals of the environment. Using **D** ^σ,A^ as an initial guess, the iterative diagonalization of the Huzinaga operator, utilizing the environment density matrix of the first SCF run as a projector, **R** ^σ,B^ = **D** ^σ,B^, produces spin-orbitals of subsystem A that minimize *E*_PbE,UKS_ while keeping the orthonormality of all the orbitals of a given spin. As a final step, *E*_1,UKS_ can be evaluated with the reoptimized spin-orbitals, and also note that $\mathrm{Tr}\left\{{\tilde{D}}^{\sigma ,A}\frac{\partial E_{12,\mathrm{UKS}}}{\partial D^{\sigma ,A}}\right\}$ is evaluated in every iteration.

### Detailed Derivation of the Unified RO Equation

In this section, we recall the ROKS/ROHF technique of Filatov, Shaik, and Roothaan to support the presentation of the restricted open-shell theory within the PbE framework.^48–50^ The ensemble energy is defined as

$$E_{\Lambda ,\mathrm{ROKS}}[D]=\mathrm{Tr}\left\{D^{c}h_{\mathrm{core}}+\frac{1}{2}D^{c}\left(J[D^{c}]-\frac{1}{2}a_{\mathrm{HF},\Lambda }K[D^{c}]\right)\right\}+\mathrm{Tr}\left\{D^{o}h_{\mathrm{core}}+\frac{1}{2}D^{o}\left(aJ[D^{o}]-b\frac{1}{2}a_{\mathrm{HF},\Lambda }K[D^{o}]\right)\right\}+\mathrm{Tr}\left\{D^{o}\left(J[D^{c}]-\frac{1}{2}a_{\mathrm{HF},\Lambda }K[D^{c}]\right)\right\}+\sum_{L}c_{L}(1-a_{\mathrm{HF},\Lambda })E_{\mathrm{xc},\Lambda }[D_{L}^{\alpha },\;D_{L}^{\beta }]+V_{\mathrm{nn}}$$
95

and the pseudo-eigenvalue equation of the closed- and open-shell can be obtained if the appropriate Lagrangian is minimized:

$$F_{\Lambda ,\mathrm{ROKS}}^{c}\left[D\right]C_{k}=\underset{l}{\sum }SC_{l}{\left(\epsilon_{\Lambda ,\mathrm{ROKS}}\right)}_{lk}+\underset{n}{\sum }SC_{n}{\left(\epsilon_{\Lambda ,\mathrm{ROKS}}\right)}_{nk}$$
96

$$fF_{\Lambda ,\mathrm{ROKS}}^{o}\left[D\right]C_{m}=\underset{n}{\sum }SC_{n}{\left(\epsilon_{\Lambda ,\mathrm{ROKS}}\right)}_{nm}+\underset{l}{\sum }SC_{l}{\left(\epsilon_{\Lambda ,\mathrm{ROKS}}\right)}_{lm}$$
97

where the corresponding Fock matrices are

$$F_{\Lambda ,\mathrm{ROKS}}^{c}\left[D\right]=h_{\mathrm{core}}+J\left[D^{c}\right]-a_{\mathrm{HF},\Lambda }\frac{1}{2}K\left[D^{c}\right]+J\left[D^{o}\right]-a_{\mathrm{HF},\Lambda }\frac{1}{2}K\left[D^{o}\right]+\left(1-a_{\mathrm{HF},\Lambda }\right)V_{\mathrm{xc},\Lambda }^{c}\left[D\right]$$
98

$$F_{\Lambda ,\mathrm{ROKS}}^{o}\left[C\right]=h_{\mathrm{core}}+aJ\left[D^{o}\right]-a_{\mathrm{HF},\Lambda }b\frac{1}{2}K\left[D^{o}\right]+J\left[D^{c}\right]-a_{\mathrm{HF},\Lambda }\frac{1}{2}K\left[D^{c}\right]+\left(1-a_{\mathrm{HF},\Lambda }\right)V_{\mathrm{xc},\Lambda }^{o}\left[D\right]$$
99

The first step of obtaining a unified equation for closed- and open-shell equations is to express the off-diagonal elements of eqs 96 and 97 as

$${\left(\epsilon_{\Lambda ,\mathrm{ROKS}}\right)}_{mk}={\left(C_{m}\right)}^{T}\left(J\left[D^{o}\right]-a_{HF,\Lambda }\frac{1}{2}K\left[D^{o}\right]\right)C_{k}+\left(\left(1-a_{HF,\Lambda }\right){\left(C_{m}\right)}^{T}V_{\mathrm{xc},\Lambda }^{c}[D]\right)C_{k}+{\left(C_{m}\right)}^{T}\left(h_{core}+J\left[D^{c}\right]-a_{HF,\Lambda }\frac{1}{2}K\left[D^{c}\right]\right)C_{k}$$
100

$${\left(\epsilon_{\Lambda ,\mathrm{ROKS}}\right)}_{km}=f{\left(C_{k}\right)}^{T}\left(aJ\left[D^{o}\right]-a_{\mathrm{HF},\Lambda }b\frac{1}{2}K\left[D^{o}\right]\right)C_{m}+f\left(\left(1-a_{\mathrm{HF},\Lambda }\right){\left(C_{k}\right)}^{T}V_{\mathrm{xc},\Lambda }^{o}[D]\right)C_{m}+f{\left(C_{k}\right)}^{T}\left(h_{core}+J\left[D^{c}\right]-a_{\mathrm{HF},\Lambda }\frac{1}{2}K\left[D^{c}\right]\right)C_{m}$$
101

If eq 100 is multiplied with −*f*/(1 – *f*) and eq 101 with 1/(1 – *f*), and the equations are added together, we get

$$(\epsilon_{\Lambda ,\mathrm{ROKS}}{)}_{mk}=-f(C_{m}{)}^{T}\left\{\overset{-}{a}J[D^{o}]-a_{\mathrm{HF},\Lambda }\overset{-}{b}\frac{1}{2}K[D^{o}]\right\}C_{k}-f(C_{m}{)}^{T}\{\overset{-}{c}(1-a_{\mathrm{HF},\Lambda })(V_{\mathrm{xc},\Lambda }^{c}[D]-V_{\mathrm{xc},\Lambda }^{o}[D])\}C_{k}$$
102

where *a̅*, *b̅*, and *c̅* are defined as

$$\overset{-}{a}=\frac{1-a}{1-f},\overset{-}{b}=\frac{1-b}{1-f},\overset{-}{c}=\frac{1}{1-f}$$
103

To express the off-diagonal multipliers in eq 102, the following Hermitian coupling operator definitions are introduced for the Coulomb and exact-exchange operators, respectively,

$$N\left[R_{i}\right]C_{p}=\omega_{i}\left(SR_{i}J\left[D^{o}\right]+\delta_{pi}J\left[D^{o}\right]+J\left[D^{o}\right]R_{i}S\right)C_{p}$$
104

$$M\left[R_{i}\right]C_{p}=\omega_{i}a_{\mathrm{HF},\Lambda }\left(SR_{i}K\left[D^{o}\right]+\delta_{pi}K\left[D^{o}\right]+K\left[D^{o}\right]R_{i}S\right)C_{p}$$
105

where ω_*i*_ is defined as

$$\omega_{i}=\underset{k}{\sum }\delta_{ki}+\underset{n}{\sum }f\delta_{ni}$$
106

The effect of the Coulomb coupling operators on various orbitals can be read as follows, where note that factor *f* is accounted for when the open-shell orbitals are used for the projectors:

$$\left(N\left[R_{k}\right]-\delta_{lk}J\left[D^{o}\right]\right)C_{l}=\left(SR_{k}J\left[D^{o}\right]+J\left[D^{o}\right]R_{k}S\right)C_{l}$$
107

$$N\left[R_{k}\right]C_{m}=\left(SR_{k}J\left[D^{o}\right]+J\left[D^{o}\right]R_{k}S\right)C_{m}$$
108

$$\left(N\left[R_{n}\right]-f\delta_{nm}J\left[D^{o}\right]\right)C_{m}=f\left(SR_{n}J\left[D^{o}\right]+J\left[D^{o}\right]R_{n}S\right)C_{m}$$
109

$$N\left[R_{n}\right]C_{k}=f\left(SR_{n}J\left[D^{o}\right]+J\left[D^{o}\right]R_{n}S\right)C_{k}$$
110

The exact-exchange coupling operators can be written as

$$\left(M_{\Lambda }\left[R_{k}\right]-a_{\mathrm{HF},\Lambda }\delta_{lk}K\left[D^{o}\right]\right)C_{l}=a_{\mathrm{HF},\Lambda }\left(SR_{k}K\left[D^{o}\right]+K\left[D^{o}\right]R_{k}S\right)C_{l}$$
111

$$M_{\Lambda }\left[R_{k}\right]C_{m}=a_{\mathrm{HF},\Lambda }\left(SR_{k}K\left[D^{o}\right]+K\left[D^{o}\right]R_{k}S\right)C_{m}$$
112

$$\left(M_{\Lambda }\left[R_{n}\right]-a_{\mathrm{HF},\Lambda }f\delta_{nm}K\left[D^{o}\right]\right)C_{m}=fa_{\mathrm{HF},\Lambda }\left(SR_{n}K\left[D^{o}\right]+K\left[D^{o}\right]R_{n}S\right)C_{m}$$
113

$$M_{\Lambda }\left[R_{n}\right]C_{k}=fa_{\mathrm{HF},\Lambda }\left(SR_{n}K\left[D^{o}\right]+K\left[D^{o}\right]R_{n}S\right)C_{k}$$
114

and the coupling terms for the exchange–correlation potentials are

$$V_{\Lambda ,i}^{c}\left[D\right]C_{i}=\left(1-a_{\mathrm{HF},\Lambda }\right)\underset{l}{\sum }\left\{SR_{l}\left(V_{\mathrm{xc},\Lambda }^{c}\left[D\right]-V_{\mathrm{xc},\Lambda ,i}\right)+\left(V_{\mathrm{xc},\Lambda }^{c}\left[D\right]-\frac{f}{\omega_{i}}V_{\mathrm{xc},\Lambda ,i}\right)R_{l}S\right\}C_{i}$$
115

$$V_{\Lambda ,i}^{o}\left[D\right]C_{i}=f\left(1-a_{\mathrm{HF},\Lambda }\right)\underset{n}{\sum }\left\{SR_{n}\left(V_{\mathrm{xc},\Lambda ,i}-V_{\mathrm{xc},\Lambda }^{o}\left[D\right]\right)+\left(\frac{1}{\omega_{i}}V_{\mathrm{xc},\Lambda ,i}-V_{\mathrm{xc},\Lambda }^{o}\left[D\right]\right)R_{n}S\right\}C_{i}$$
116

where *V*_xc,Λ,*i*_ is defined as

$$V_{\mathrm{xc},\Lambda ,i}=V_{\mathrm{xc},\Lambda }^{c}\left[D\right]\underset{k}{\sum }\delta_{ki}+\underset{m}{\sum }V_{\mathrm{xc},\Lambda }^{o}\left[D\right]\delta_{mi}$$
117

The equation that express the off-diagonal multipliers of the closed-shell, that is, eq 96, can be written as

$$\left(\underset{m}{\sum }\overset{-}{a}N\left[R_{m}\right]-\underset{m}{\sum }\overset{-}{b}\frac{1}{2}M_{\Lambda }\left[R_{m}\right]+\overset{-}{c}V_{\Lambda ,k}^{o}\right)C_{k}=-\underset{m}{\sum }SC_{m}{\left(\epsilon_{\Lambda ,\mathrm{ROKS}}\right)}_{mk}$$
118

where eqs 110, 114, and 116 is used, and the open-shell part in eq 97 is

$$\left(\underset{l}{\sum }\overset{-}{a}N\left[R_{l}\right]-\underset{l}{\sum }\overset{-}{b}\frac{1}{2}M_{\Lambda }\left[R_{l}\right]+\overset{-}{c}V_{\Lambda ,m}^{c}\right)C_{m}=-\frac{1}{f}\underset{k}{\sum }SC_{k}{\left(\epsilon_{\Lambda ,\mathrm{ROKS}}\right)}_{km}$$
119

where eqs 108, 112, and 115 is utilized, and indeed, the multipliers of eq 102 can be recovered if the above equations are multiplied with the appropriate orbitals. Adding eq 118 and eq 119 to eq 96 and eq 97, respectively, the closed- and open-shell equations can be recast as

$$\left\{h_{\mathrm{core}}+J\left[D^{c}\right]-a_{\mathrm{HF},\Lambda }\frac{1}{2}K\left[D^{c}\right]+J\left[D^{o}\right]-a_{\mathrm{HF},\Lambda }\frac{1}{2}K\left[D^{o}\right]+\left(1-a_{\mathrm{HF},\Lambda }\right)V_{\mathrm{xc},\Lambda }^{c}\left[D\right]\right\}C_{k}+\left\{\underset{m}{\sum }\overset{-}{a}N\left[R_{m}\right]-\underset{m}{\sum }\overset{-}{b}\frac{1}{2}M_{\Lambda }\left[R_{m}\right]+\overset{-}{c}V_{\Lambda ,k}^{o}\right\}C_{k}=\underset{l}{\sum }SC_{l}{\left(\epsilon_{\Lambda ,\mathrm{ROKS}}\right)}_{lk}$$
120

$$\left\{h_{\mathrm{core}}+aJ\left[D^{o}\right]-a_{\mathrm{HF},\Lambda }b\frac{1}{2}K\left[D^{o}\right]+J\left[D^{c}\right]-a_{\mathrm{HF},\Lambda }\frac{1}{2}K\left[D^{c}\right]+\left(1-a_{\mathrm{HF},\Lambda }\right)V_{\mathrm{xc},\Lambda }^{o}\left[D\right]\right\}C_{m}+\left\{\underset{l}{\sum }\overset{-}{a}N\left[R_{l}\right]-\underset{l}{\sum }\overset{-}{b}\frac{1}{2}M_{\Lambda }\left[R_{l}\right]+\overset{-}{c}V_{\Lambda ,m}^{c}\right\}C_{m}=\frac{1}{f}\underset{n}{\sum }SC_{n}{\left(\epsilon_{\Lambda ,\mathrm{ROKS}}\right)}_{nm}$$
121

and these equations are the exact solutions of the variational problem of the shells. In order to get a uniform equation, two additional equations are formed:

$$A_{\Lambda ,k}^{c}C_{k}=\underset{l}{\sum }SC_{l}{\left(\zeta_{\Lambda }\right)}_{lk}$$
122

$$A_{\Lambda ,m}^{o}C_{m}=\frac{1}{f}\underset{n}{\sum }SC_{n}{\left(\zeta_{\Lambda }\right)}_{nm}$$
123

for the closed- and open-shell, respectively, where the operators are

$$A_{\Lambda ,k}^{c}=\sum_{l}\overset{-}{a}(N[R_{l}]-\delta_{lk}J[D^{o}])-\sum_{l}\overset{-}{b}\frac{1}{2}(M_{\Lambda }[R_{l}]-a_{\mathrm{HF},\Lambda }\delta_{lk}K[D^{o}])+(\overset{-}{c}V_{\Lambda ,k}^{c}-(1-a_{\mathrm{HF},\Lambda })V_{\mathrm{xc},\Lambda }^{c}[D])$$
124

$$A_{\Lambda ,m}^{o}=\sum_{n}\overset{-}{a}(N[R_{n}]-f\delta_{nm}J[D^{o}])-\sum_{n}\overset{-}{b}\frac{1}{2}(M_{\Lambda }[R_{n}]-a_{\mathrm{HF},\Lambda }f\delta_{nm}K[D^{o}])+(\overset{-}{c}V_{\Lambda ,m}^{o}-(1-a_{\mathrm{HF},\Lambda })V_{\mathrm{xc},\Lambda }^{o}[D])$$
125

The eigenvalues of these equations can be calculated using eqs 107, 111, and 115:

$$(\zeta_{\Lambda }{)}_{lk}=(C_{l}{)}^{T}A_{\Lambda ,k}^{c}C_{k}=(C_{l}{)}^{T}(2\overset{-}{a}J[D^{o}]-a_{\mathrm{HF},\Lambda }\overset{-}{b}K[D^{o}])C_{k}$$
126

moreover, eqs 109, 113, and 116 are used for the multipliers of the open-shell part:

$$(\zeta_{\Lambda }{)}_{nm}=f(C_{n}{)}^{T}A_{\Lambda ,m}^{o}C_{m}=f(C_{n}{)}^{T}(2\overset{-}{a}J[D^{o}]-a_{\mathrm{HF},\Lambda }\overset{-}{b}K[D^{o}])C_{m}$$
127

Note that the closed-shell/open-shell blocks and the open-shell/open-shell block of the operator in eq 124 are zero in the MO basis. Similarly, the closed-shell/open-shell blocks and the closed-shell/closed-shell block of the operator in eq 125 are also zero, thus these operators only transform orbitals in their corresponding subspaces. Adding eq 124 to eq 120, and eq 125 to eq 121, we get

$$F_{i,\Lambda ,\mathrm{ROKS}}C_{i}^{\prime }=\underset{k}{\sum }SC_{k}^{\prime }\left\{{\left(\epsilon_{\Lambda ,\mathrm{ROKS}}\right)}_{ki}+{\left(\zeta_{\Lambda }\right)}_{ki}\right\}+\frac{1}{f}\underset{m}{\sum }SC_{m}^{\prime }\left\{{\left(\epsilon_{\Lambda ,\mathrm{ROKS}}\right)}_{mi}+{\left(\zeta_{\Lambda }\right)}_{mi}\right\}$$
128

where **C**_*i*_ *′* is not identical with **C**_*k*_ and **C**_*n*_, but related by an unitary transformation, and the ROKS Fock matrix is

$$F_{i,\Lambda ,\mathrm{ROKS}}=h_{\mathrm{core}}+J[D]-a_{\mathrm{HF},\Lambda }\frac{1}{2}K[D]+\overset{-}{c}V_{\Lambda }^{t}+\sum_{j}\left(\overset{-}{a}\{N[R_{j}]-\delta_{ji}J[D^{o}]\}-\overset{-}{b}\frac{1}{2}\{M_{\Lambda }[R_{j}]-a_{\mathrm{HF},\Lambda }\delta_{ji}K[D^{o}]\}\right)$$
129

where **V**_Λ_^t^ is defined as

$$V_{\Lambda }^{t}=\underset{i}{\sum }\left(V_{\Lambda ,i}^{c}+V_{\Lambda ,i}^{o}\right)R_{i}S$$
130

Indeed, the form of the operator for the closed-shell and open-shell orbitals is the same, which can be seen if one adds together the operators in eqs 96, 118, and 122 for the closed-shell operator and eqs 97, 119, and 123 for the open-shell operator, and exploits the following relations: (1 – *a*) = (1 – *f*)*a̅*, (1 – *b*) = (1 – *f*)*b̅*, **J**[**D** ^c^] + **J**[**D** ^o^] – **J**[**D** ^o^] = **J**[**D**] – **J**[**D** ^o^], and the corresponding relations for the exact-exchange part.

Note that the exchange–correlation potential matrix in eq 130 is not symmetric, thus the operator is expanded with the projector of virtual orbitals, and additional terms that does not have an effect on the occupied space

$$V_{\Lambda }^{t\prime }=\underset{p}{\sum }\left(V_{\Lambda ,p}^{c}+V_{\Lambda ,p}^{o}\right)R_{p}S-f\underset{l}{\sum }\underset{s}{\sum }SR_{l}V_{\mathrm{xc},\Lambda }^{c}\left[D\right]R_{s}S+\underset{n}{\sum }\underset{s}{\sum }SR_{n}V_{\mathrm{xc},\Lambda }^{o}\left[D\right]R_{s}S+\frac{1}{\mathrm{c̅}}\underset{t}{\sum }\underset{s}{\sum }SR_{t}V_{\mathrm{xc},\Lambda }^{c}\left[D\right]R_{s}S$$
131

where it is exploited that

$$V_{\mathrm{xc},\Lambda ,s}^{o}=\frac{1}{2f}\sum_{L}c_{L}(n_{s,L}^{\alpha }V_{\mathrm{xc},\Lambda ,L}^{\alpha }[D_{L}^{\alpha },\;D_{L}^{\beta }]+n_{s,L}^{\beta }V_{\mathrm{xc},\Lambda ,L}^{\beta }[D_{L}^{\alpha },\;D_{L}^{\beta }])=0$$
132

because *n*_*s*,*L*_^α^ = *n*_*s*,*L*_^β^ = 0. Subsequently, the expanded operator is symmetrized, which produces the Hermitian, final form of the exchange–correlation potential:

$$\frac{1}{2}\left(V_{\Lambda }^{t\prime }+{\left(V_{\Lambda }^{t\prime }\right)}^{T}\right)=\left(1-a_{\mathrm{HF},\Lambda }\right)\frac{1}{\mathrm{c̅}}V_{\mathrm{xc},\Lambda }^{c}\left[D\right]+\left(1-a_{\mathrm{HF},\Lambda }\right)\gamma^{T}\left(V_{\mathrm{xc},\Lambda }^{c}\left[D\right]-V_{\mathrm{xc},\Lambda }^{o}\left[D\right]\right)R^{o}S+\left(1-a_{\mathrm{HF},\Lambda }\right)SR^{o}\left(V_{\mathrm{xc},\Lambda }^{c}\left[D\right]-V_{\mathrm{xc},\Lambda }^{o}\left[D\right]\right)\gamma$$
133

where the relation of the projectors,

$$1=R^{c}+R^{o}+R^{v}$$
134

$$\begin{array}{ccc} R^{c}=\sum_{k}R_{k}, & R^{o}=\sum_{m}R_{m}, & R^{v}=\sum_{s}R_{s} \end{array}$$
135

is used, and the definition of the **γ** is

$$\gamma =R^{c}S-\frac{1}{\overset{-}{c}}1+\frac{1}{2\overset{-}{c}}R^{o}S$$
136

Using the new definition of the exchange–correlation potential in eq 133, the operator in eq 129 can be rewritten as

$$F_{\Lambda ,\mathrm{ROKS}}\left[D\right]=F_{\Lambda }^{c}\left[D\right]-Q_{\Lambda }\left[D^{o}\right]+T_{\Lambda }\left[D\right]$$
137

where matrices **F**_Λ_^c^, **Q**_Λ_, and **T**_Λ_ are

$$F_{\Lambda }^{c}\left[D\right]=h_{\mathrm{core}}+J\left[D\right]-a_{\mathrm{HF},\Lambda }\frac{1}{2}K\left[D\right]+\left(1-a_{\mathrm{HF},\Lambda }\right)V_{\mathrm{xc},\Lambda }^{c}\left[D\right]$$
138

$$Q_{\Lambda }[D^{o}]=\overset{-}{a}J[D^{o}]-a_{\mathrm{HF},\Lambda }\overset{-}{b}\frac{1}{2}K[D^{o}]$$
139

$$T_{\Lambda }[D]=SR^{c}Q_{\Lambda }[D^{o}]+Q_{\Lambda }[D^{o}]R^{c}S+f(SR^{o}Q_{\Lambda }[D^{o}]+Q_{\Lambda }[D^{o}]R^{o}S)+(1-a_{\mathrm{HF},\Lambda })\overset{-}{c}\gamma^{T}(V_{\mathrm{xc},\Lambda }^{c}[D]-V_{\mathrm{xc},\Lambda }^{o}[D])R^{o}S+(1-a_{\mathrm{HF},\Lambda })\overset{-}{c}SR^{o}(V_{\mathrm{xc},\Lambda }^{c}[D]-V_{\mathrm{xc},\Lambda }^{o}[D])\gamma$$
140

and the final form of the eigenvalue equation is

$$F_{\Lambda ,\mathrm{ROKS}}\left[D\right]C_{i}^{\prime }=\frac{1}{\omega_{i}}\underset{j}{\sum }SC_{j}^{\prime }{\left(\epsilon_{\Lambda ,\mathrm{ROKS}}^{\prime }\right)}_{ji}$$
141

where (ϵ_Λ,ROKS_ *′*)_*ji*_ = (ϵ_Λ,ROKS_)_*ji*_ + (ζ_Λ_)_*ji*_.

### Other Multilevel Techniques

In addition to presenting the new theoretical and algorithmic developments, an informative comparison of the widely used multilevel techniques is also a goal of this paper. Here, a brief overview of the methods is provided that serve as competitors of the Huzinaga-equation-based PbE approach.

The most well-known technique is the ONIOM family of multilevel methods,^19^ and its simplest form, called mechanical embedding (ME), defines the composite energy as follows:

$$E_{\mathrm{ONIOM}‐\mathrm{ME}}=E_{2}[D_{2}^{\mathrm{AB}}]-E_{2}[{\overset{-}{D}}_{2}^{A},\;\{R^{L}\}]+E_{1}[{\overset{-}{D}}_{1}^{A},\;\{R^{L}\}]$$
142

where the bar over the density matrices denotes truncated AO basis set, that is, basis functions are only centered on atoms of the active subsystems. Besides the atoms of the real system, additional link atoms are inserted in the case of covalently bound subsystems to handle the dangling bonds. The link atoms, which are typically hydrogen atoms, are set between atoms of different layers and their position is denoted by {**R** ^L^}. Note that the density matrices in the ONIOM-ME approach are obtained in three separate mean-field calculations, and the subsystem computations are carried out in vacuum. Truncating a system to a computationally tractable size is a common approach in quantum chemical applications that relates to ONIOM-ME: it is called cluster model technique or vacuum-embedding, where only *E*_1_ is evaluated in eq 142. A more sophisticated approach is the electronic embedding (EE) variant of ONIOM defined by energy expression

$$E_{\mathrm{ONIOM}‐\mathrm{EE}}=E_{2}[D_{2}^{\mathrm{AB}}]-E_{2}[{\overset{-}{D}}_{2}^{A},\;\{R^{L}\},\;\{R^{B}\},\;\{q^{B}\}]+E_{1}[{\overset{-}{D}}_{1}^{A},\;\{R^{L}\},\;\{R^{B}\},\;\{q^{B}\}]$$
143

where subsystem calculations are carried out in the presence of point charges, which are placed at the positions of the environment atoms, {**R** ^B^}, with magnitudes {*q* ^B^}. These charges can either be predefined or derived from the low-level supersystem calculation. In practice, the charges that are the closest to the link atoms are omitted, and a charge redistribution scheme is often applied to ensure integer charges for the subsystems.

The most sophisticated multilevel method in this study relies on our local natural orbital (LNO)-based correlation technique. The details of the algorithm can be found in ref 60, here only its core components are presented. In the case of LNO–CCSD­(T), the energy is expressed as the sum of the HF energy, *E*_HF_, and the correlation energy as

$$E_{\mathrm{LNO}‐\mathrm{CCSD}\left(T\right)}=E_{\mathrm{HF}}+\overset{\mathrm{weak}}{\underset{ij}{\sum }}E_{\mathrm{PD},ij}^{\mathrm{MP2}}+\underset{i}{\sum }E_{\mathrm{ED},i}^{\mathrm{MP2}}+\underset{i}{\sum }E_{\mathrm{LIS},i}^{\mathrm{CCSD}\left(T\right)}$$
144

where PD, ED, and LIS stand for pair domain, extended domain, and local interacting subspace, respectively. The summations are performed over localized occupied orbitals. After solving the HF equations and evaluating *E*_HF_, the occupied MOs are localized, and projected atomic orbitals (PAOs) are used as virtuals. For each localized MO (LMO), a primary domain is constructed, consisting of the single occupied MO and PAOs that are considered close to this MO. Then, atoms are assigned to the occupied MO and virtual orbitals by the Boughton–Pulay (BP) algorithm,^83^ and subsequently, the LMO and the PAOs are projected onto the union of the AOs of the assigned atoms. Lastly, the projected MOs are orthonormalized and canonicalized. For each pair of LMO, a PD is constructed as the union of primary domains, and the multipole-approximated MP2 energy (*E*_PD,*ij*_^MP2^) is calculated within the domain. Moreover, the LMO pairs are categorized as strongly and weakly interacting pairs. The first summation in eq 144 runs over the latter. Then, EDs are constructed for each LMO using its strongly interacting pairs, and the MP2 correlation energy contribution of the LMO (*E*_ED,*i*_^MP2^) is calculated in the ED. Next, a smaller domain termed LIS is built in each ED using MP2 natural orbitals of the ED, and the CCSD­(T) method is only utilized in this reduced space to calculate correlation energy contribution of the LMO (*E*_LIS,*i*_^CCSD(T)^). Note that the described procedure can naturally be adapted to focused models by using the high-level correlation method only for those orbitals that are localized in the active subsystem, while the environment orbitals are treated at the MP2 level. This approach will be denoted as multilevel local correlation (MLC) method, and the specific technique used in this paper will be termed as LNO–CCSD­(T)-in-LMP2, where LMP2 stands for local MP2.

## Notes

The results of all the evaluated techniques (PDF)
Test system geometries in *xyz* format (ZIP)
