# Supplementary material for: Extending the Projection-Based Embedding Technique to Open-Shell Systems Using the Huzinaga Equation
Source: J Chem Theory Comput. 2025 Jul 17;21(15):7394–418. doi: 10.1021/acs.jctc.5c00687 (PMC12355723; doi:10.1021/acs.jctc.5c00687)
Supplement: Supplementary file 1 [file ct5c00687_si_001.pdf]

Extending The Projection-Based Embedding Technique to Open-Shell Systems Using The Huzinaga Equation – Electronic Supplementary Material

Bence Hégely[*,†,‡,¶] and Mihály Kállay[*,†,‡,¶]

†*Department of Physical Chemistry and Materials Science, Faculty of Chemical Technology and Biotechnology, Budapest University of Technology and Economics, Műegyetem rkp. 3., H-1111 Budapest, Hungary*

‡*HUN-REN–BME Quantum Chemistry Research Group, Műegyetem rkp. 3., H-1111 Budapest, Hungary*

¶*MTA–BME Lendület Quantum Chemistry Research Group, Műegyetem rkp. 3., H-1111 Budapest, Hungary*

E-mail: hegely.bence@vbk.bme.hu; kallay.mihaly@vbk.bme.hu

Contents

1 Results for test reaction I **3**

2 Results for test reaction II **7**

3 Results for test reaction III **11**

4 Results for test reaction IV **15**

1 Results for test reaction I

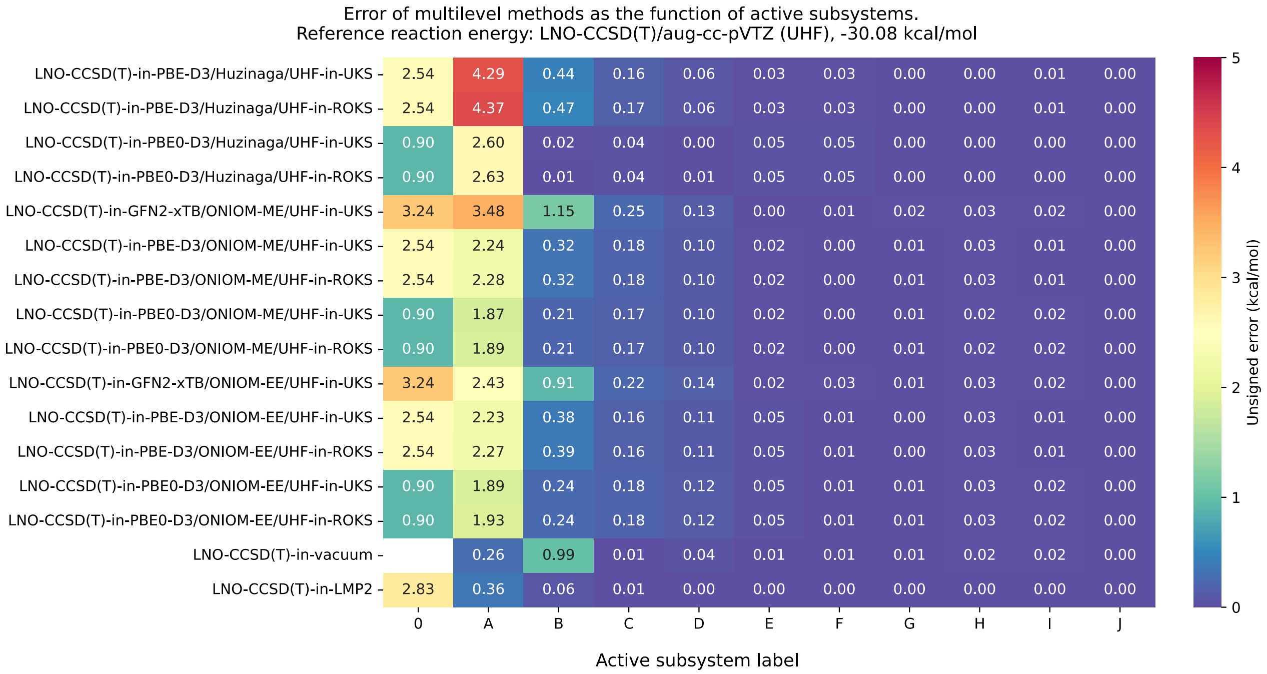

Figure S1: Unsigned errors of the multilevel methods as a function of the active subsystems for test reaction I. The meaning of the active subsystem labels is shown in Fig. 1, moreover, label "0" denotes results of the low-level methods. The leftmost column shows the type of the wave function Ansatz used for the open-shell molecules and the theoretical method applied at the low-level.

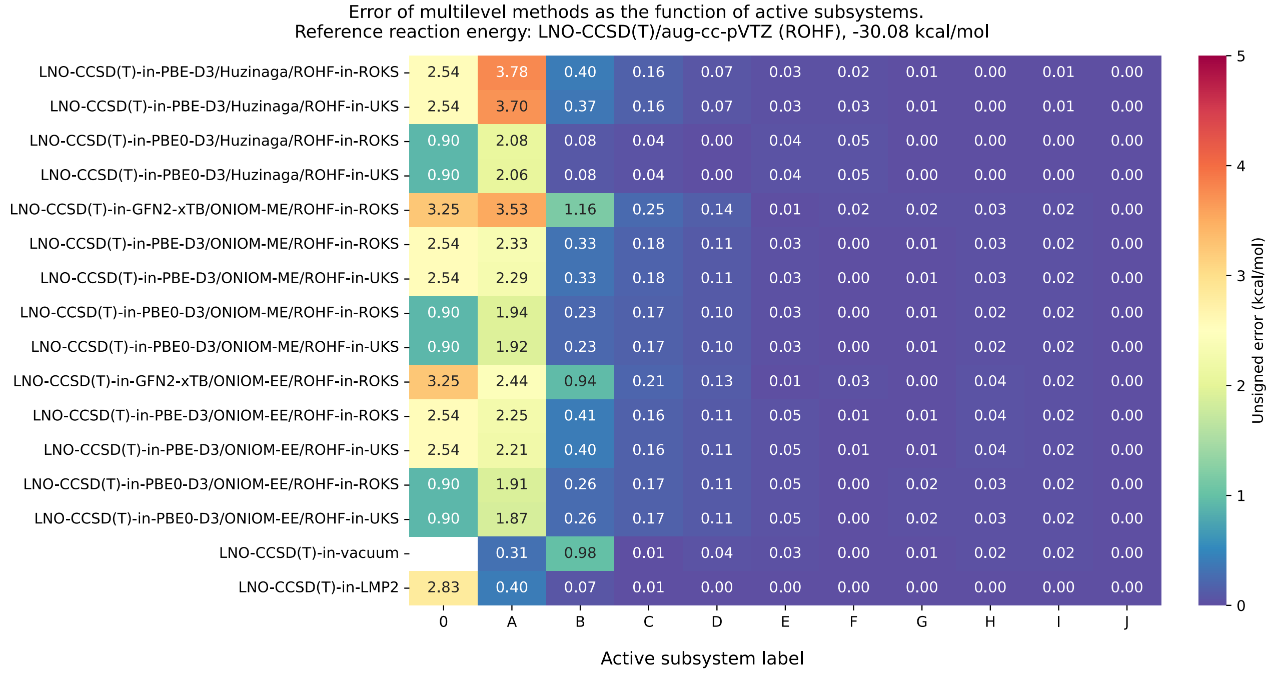

Figure S2: Unsigned errors of the multilevel methods as a function of the active subsystems for test reaction I. For a detailed explanation of the axes, please refer to Fig. S1.

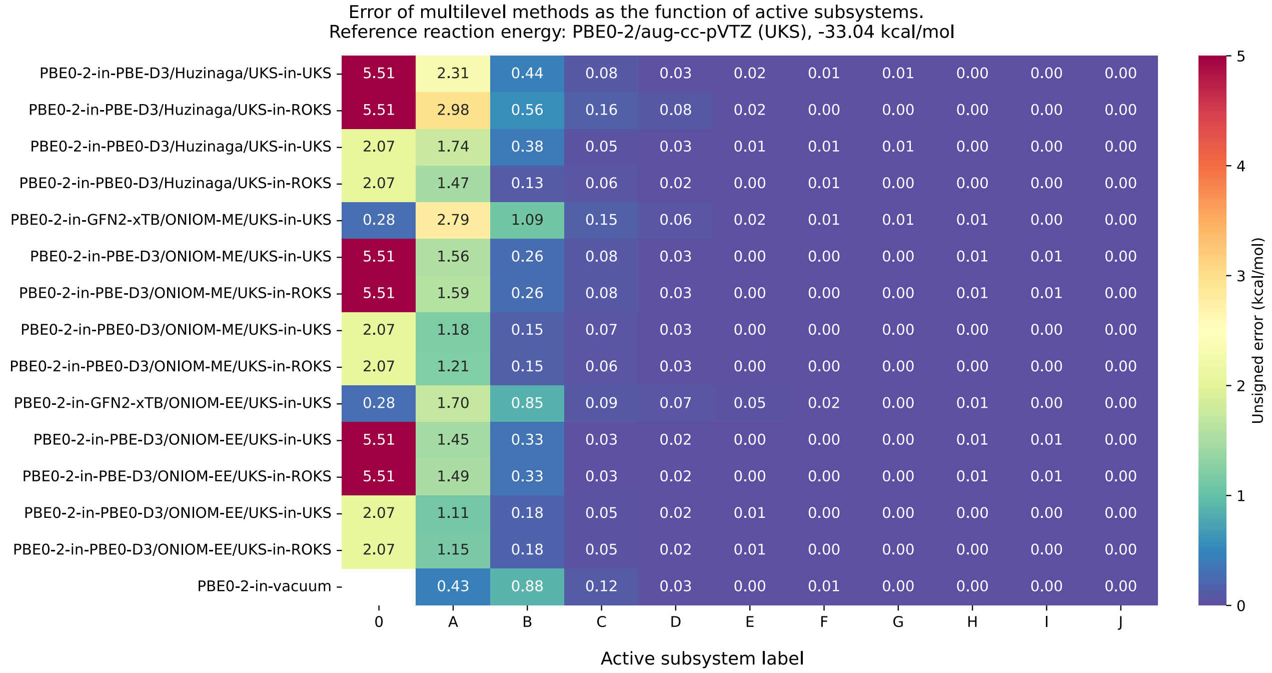

Figure S3: Unsigned errors of the multilevel methods as a function of the active subsystems for test reaction I. For a detailed explanation of the axes, please refer to Fig. S1.

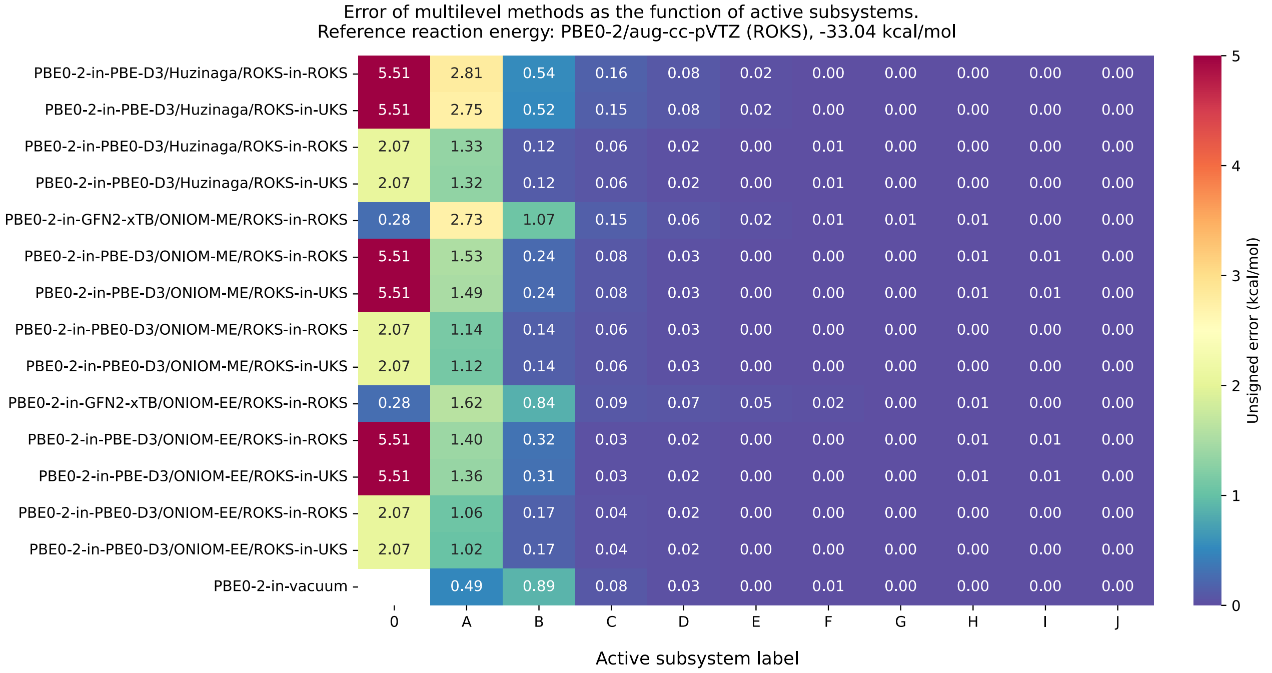

Figure S4: Unsigned errors of the multilevel methods as a function of the active subsystems for test reaction I. For a detailed explanation of the axes, please refer to Fig. S1.

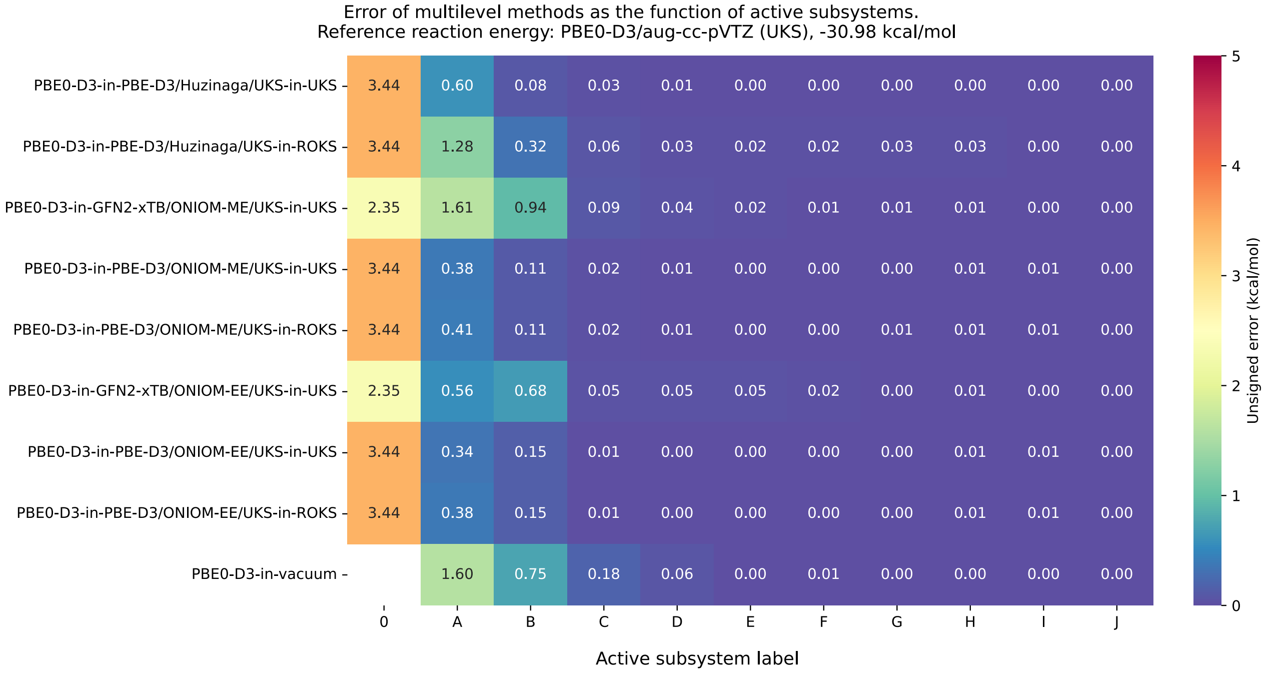

Figure S5: Unsigned errors of the multilevel methods as a function of the active subsystems for test reaction I. For a detailed explanation of the axes, please refer to Fig. S1.

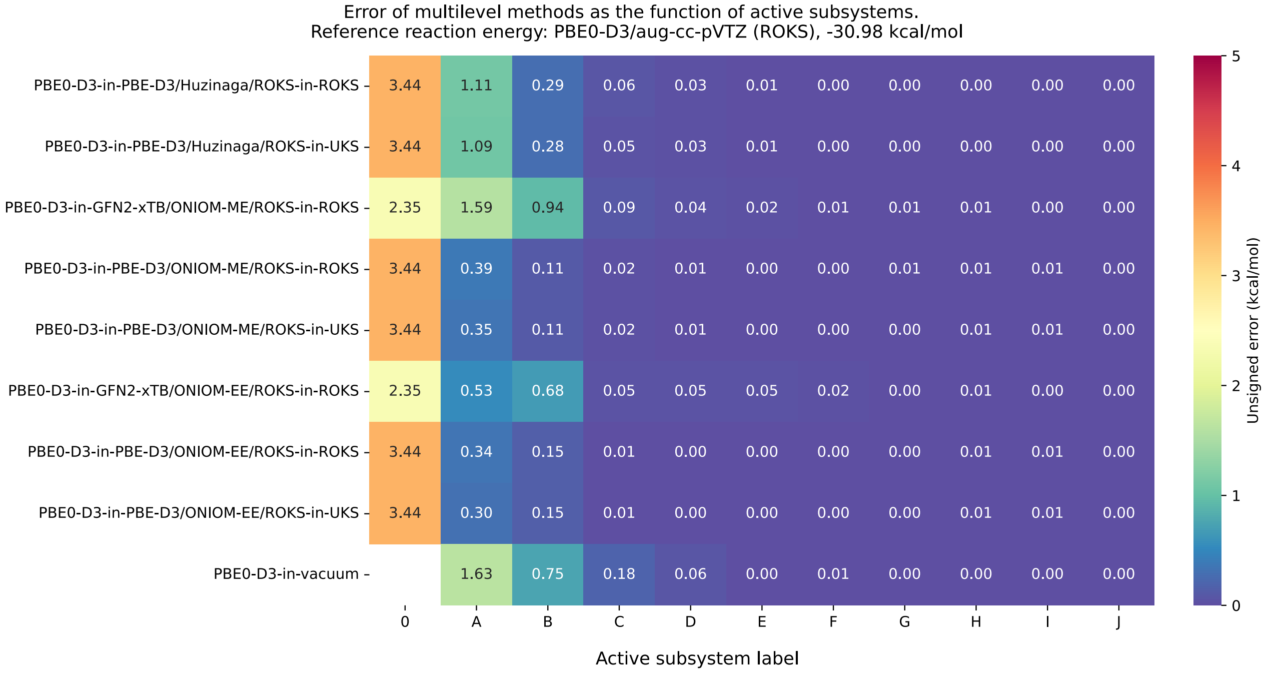

Figure S6: Unsigned errors of the multilevel methods as a function of the active subsystems for test reaction I. For a detailed explanation of the axes, please refer to Fig. S1.

2 Results for test reaction II

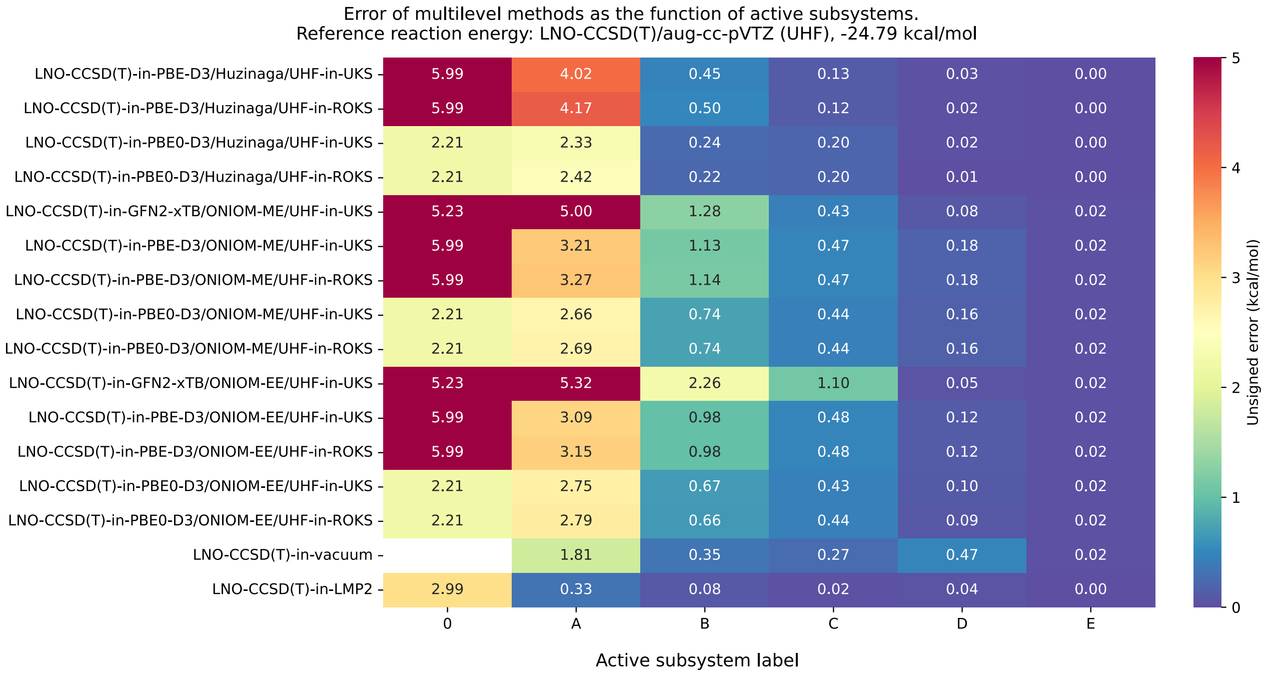

Figure S7: Unsigned errors of the multilevel methods as a function of the active subsystems for test reaction II. The meaning of the active subsystem labels is shown in Fig. 4, moreover, label "0" denotes results of the low-level methods. The leftmost column shows the type of the wave function Ansatz used for the open-shell molecules and the theoretical method applied at the low-level.

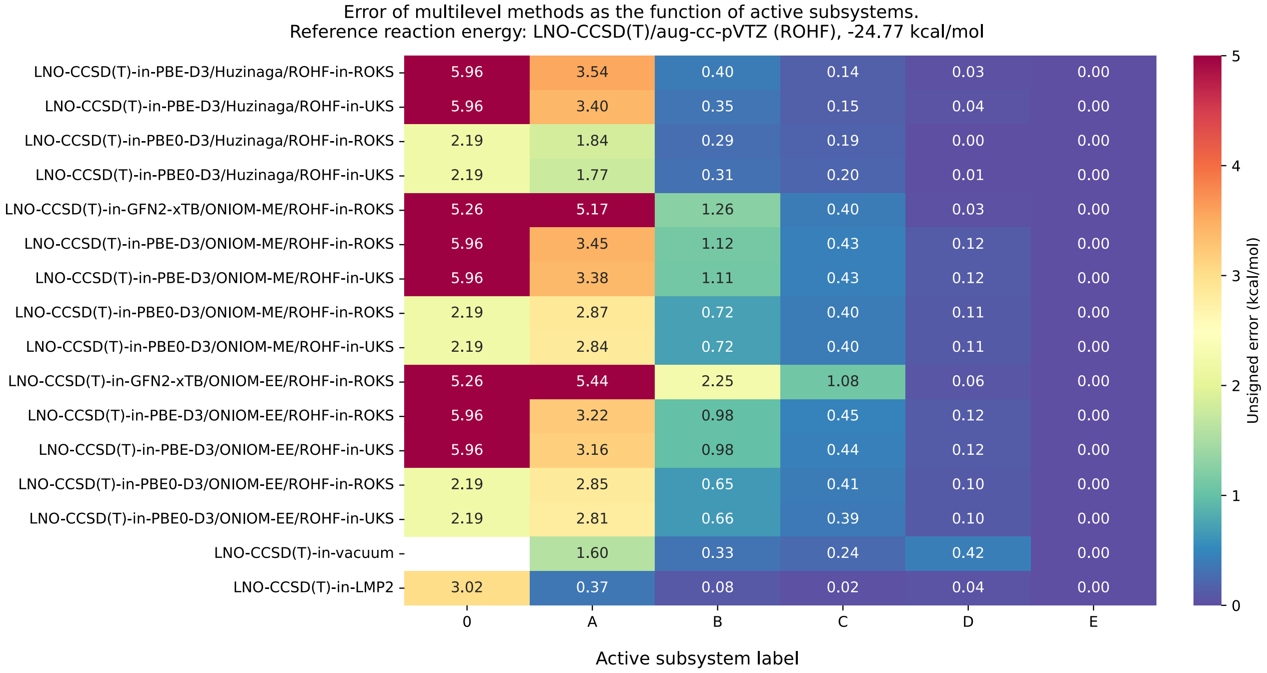

Figure S8: Unsigned errors of the multilevel methods as a function of the active subsystems for test reaction II. For a detailed explanation of the axes, please refer to Fig. S7.

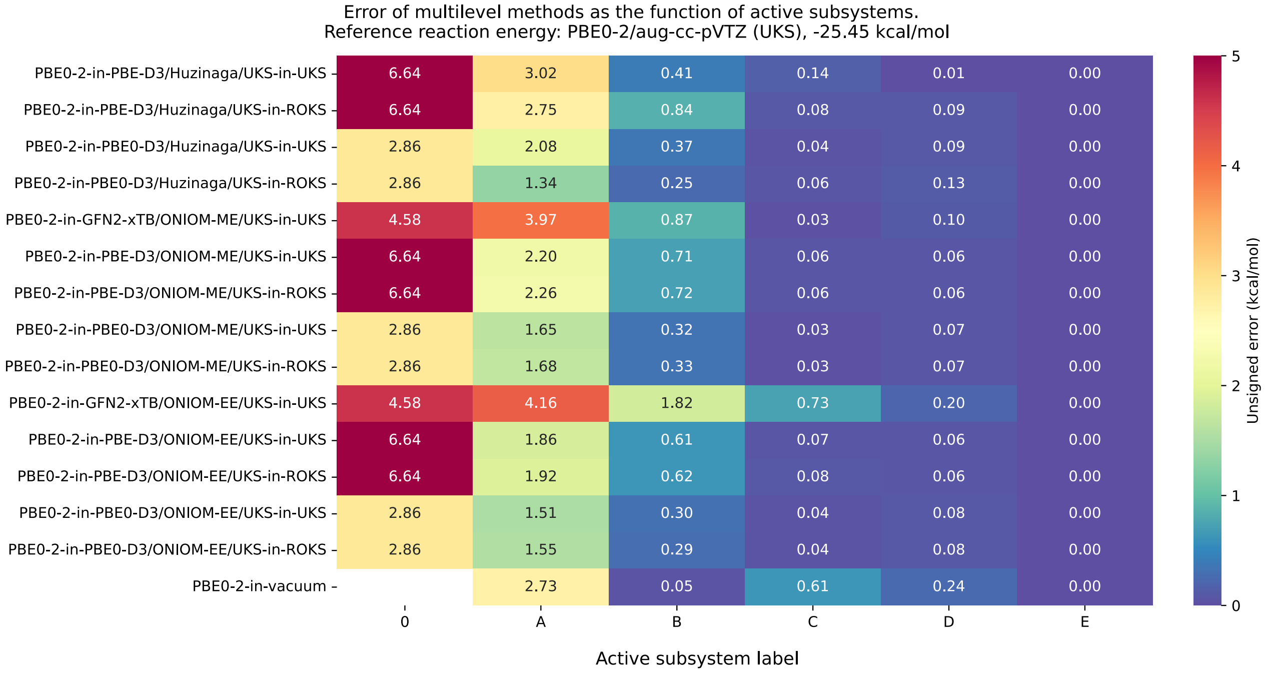

Figure S9: Unsigned errors of the multilevel methods as a function of the active subsystems for test reaction II. For a detailed explanation of the axes, please refer to Fig. S7.

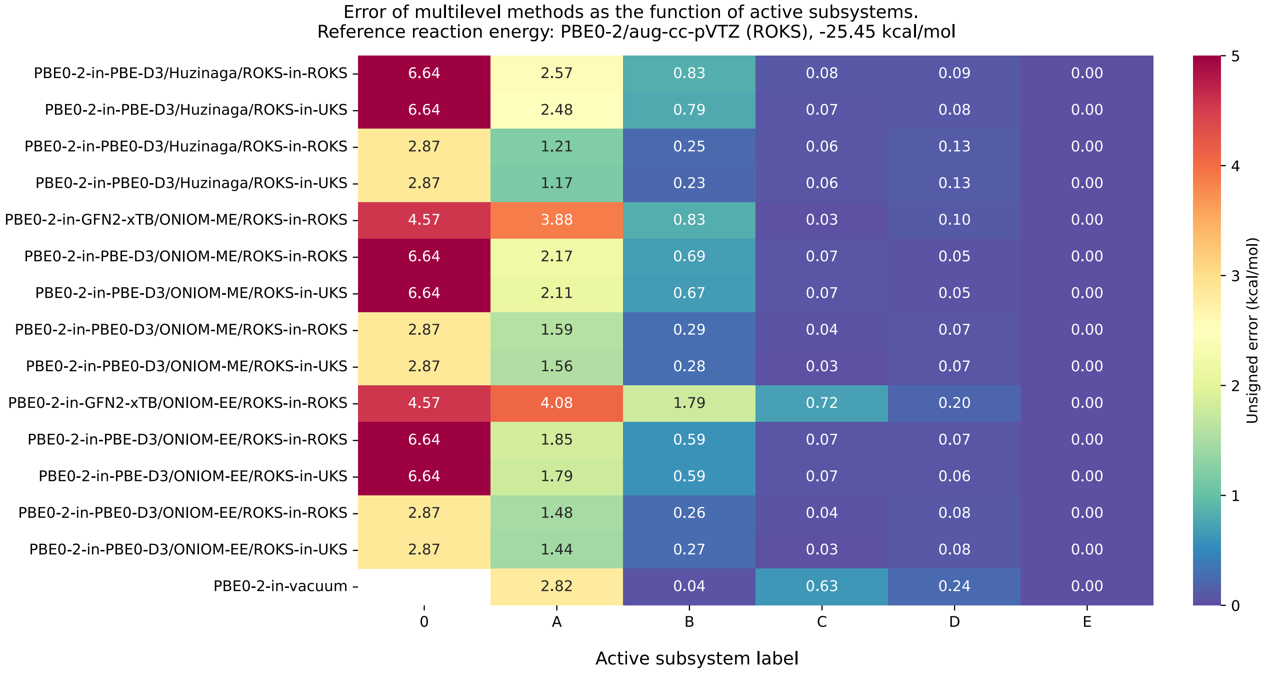

Figure S10: Unsigned errors of the multilevel methods as a function of the active subsystems for test reaction II. For a detailed explanation of the axes, please refer to Fig. S7.

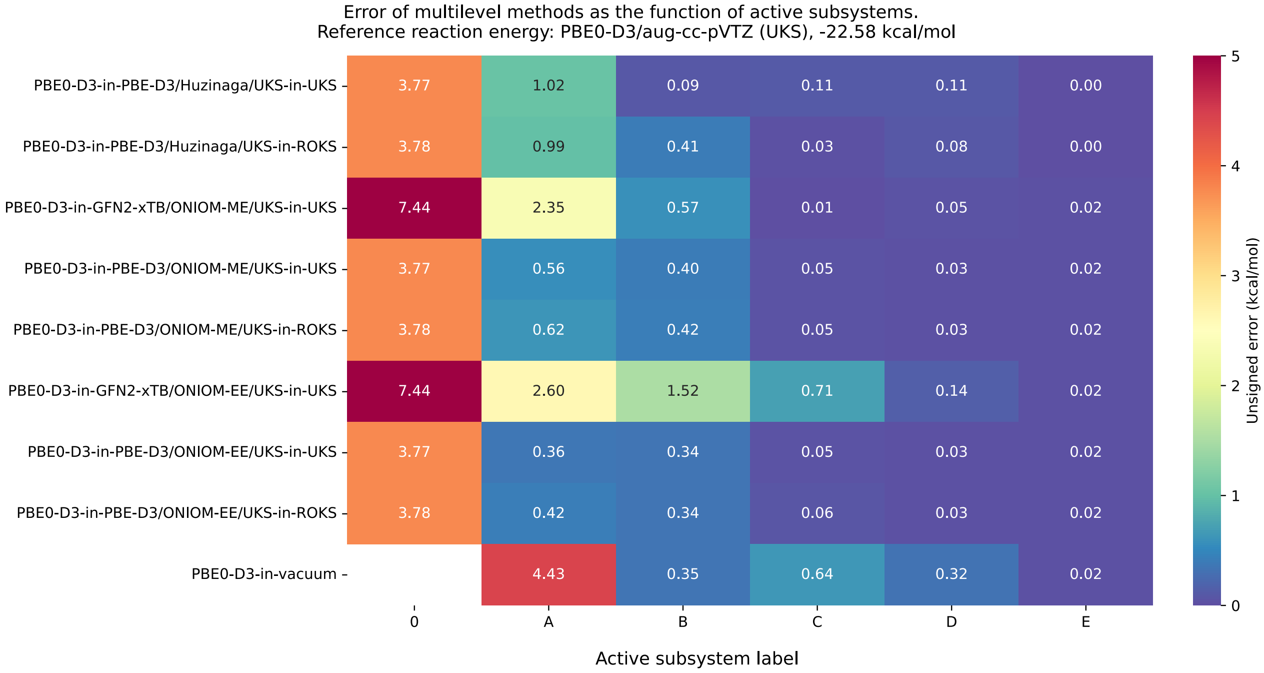

Figure S11: Unsigned errors of the multilevel methods as a function of the active subsystems for test reaction II. For a detailed explanation of the axes, please refer to Fig. S7.

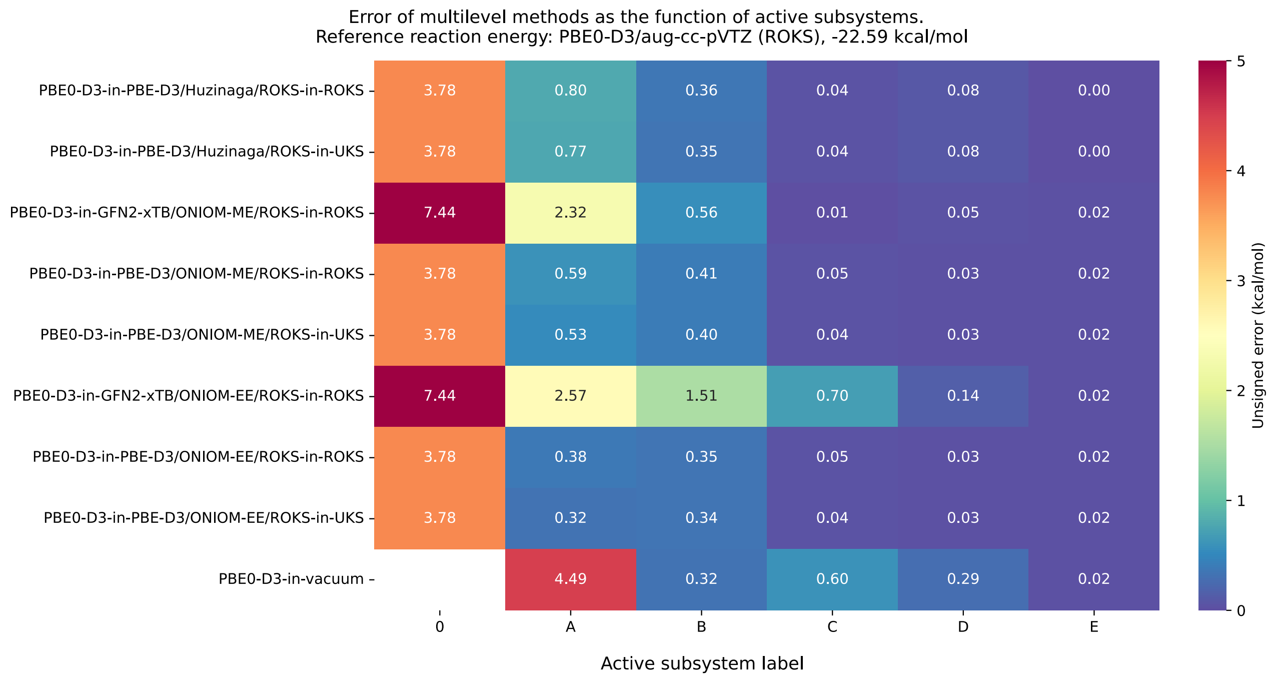

Figure S12: Unsigned errors of the multilevel methods as a function of the active subsystems for test reaction II. For a detailed explanation of the axes, please refer to Fig. S7.

3 Results for test reaction III

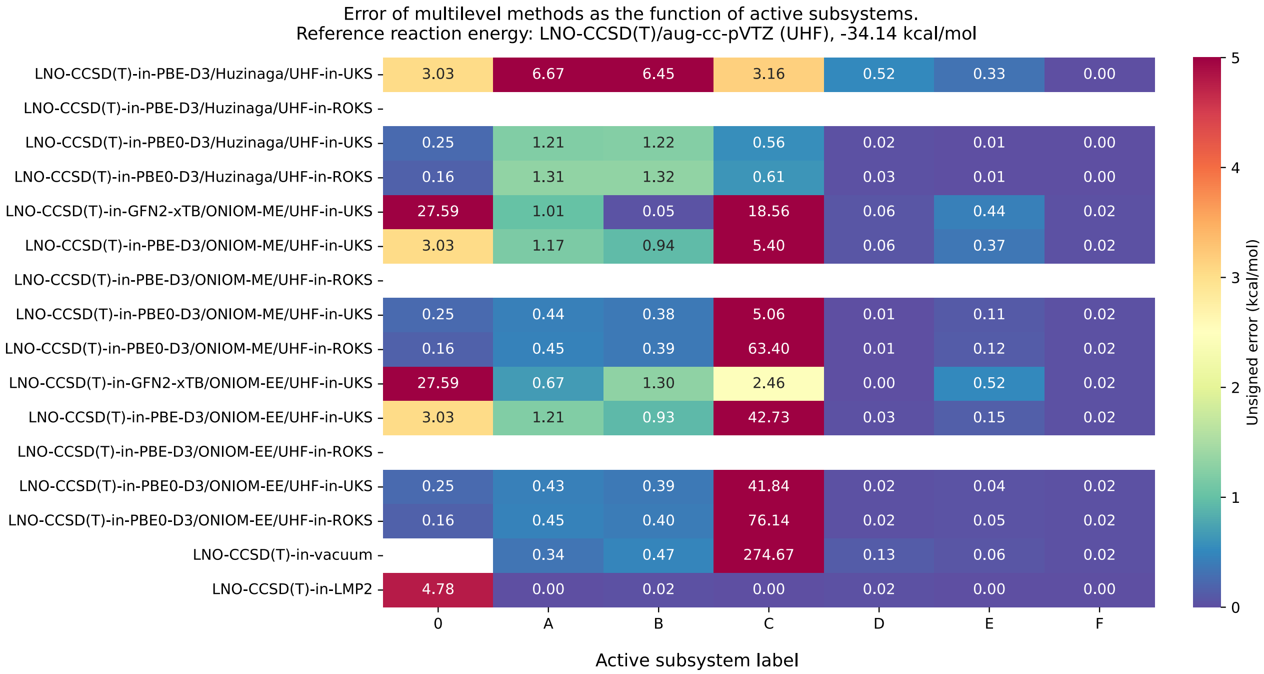

Figure S13: Unsigned errors of the multilevel methods as a function of the active subsystems for test reaction III. The meaning of the active subsystem labels is shown in Fig. 7, moreover, label "0" denotes results of the low-level methods. The leftmost column shows the type of the wave function Ansatz used for the open-shell molecules and the theoretical method applied at the low-level. Note that empty rows indicate low-level methods that did not yield SCF solutions for the doublet state.

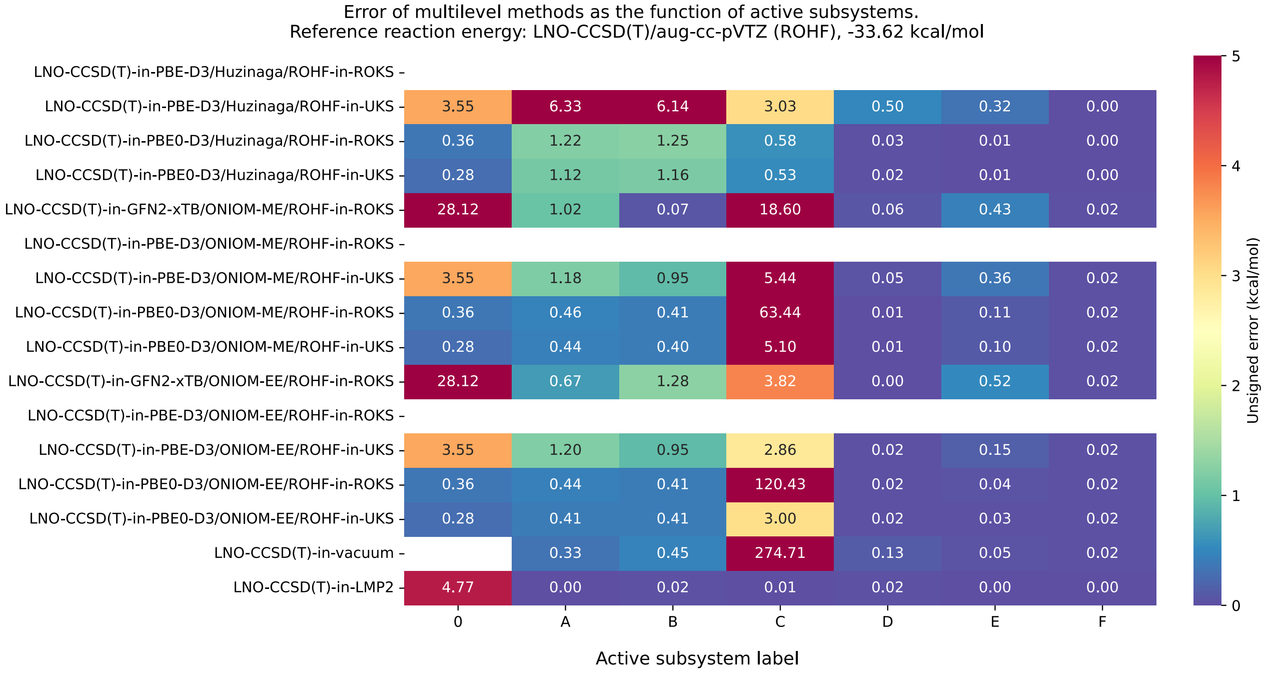

Figure S14: Unsigned errors of the multilevel methods as a function of the active subsystems for test reaction III. For a detailed explanation of the axes, please refer to Fig. S13.

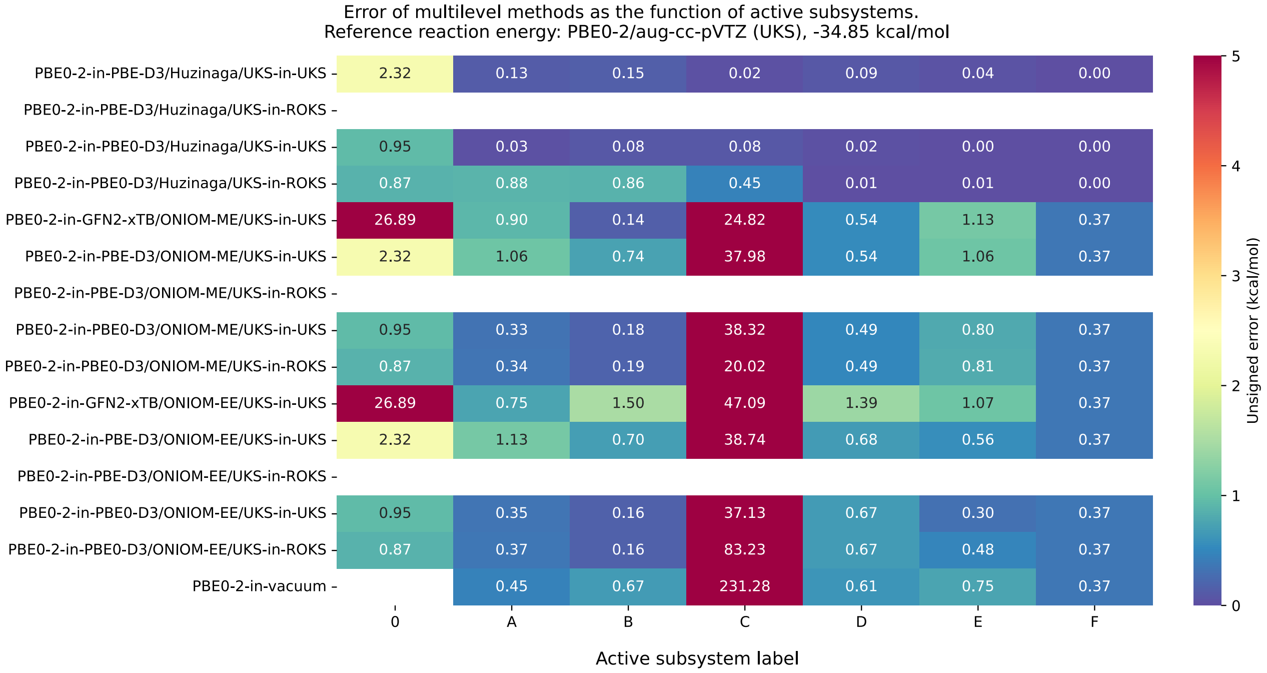

Figure S15: Unsigned errors of the multilevel methods as a function of the active subsystems for test reaction III. For a detailed explanation of the axes, please refer to Fig. S13.

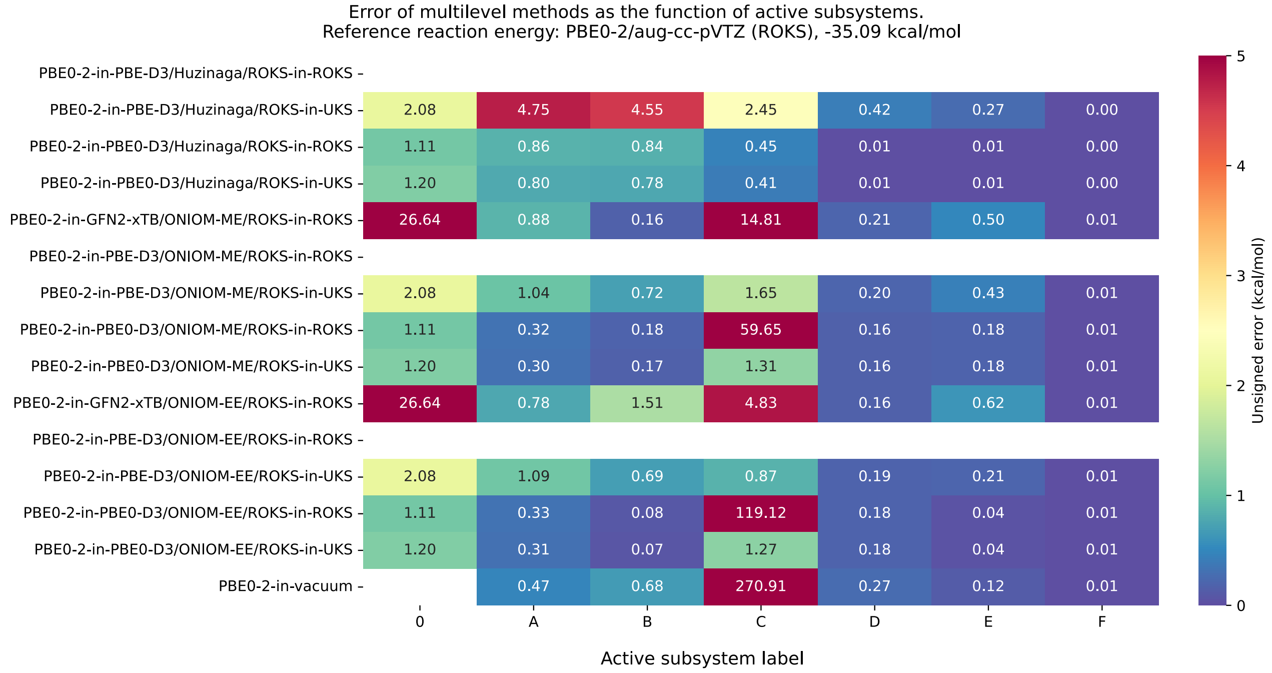

Figure S16: Unsigned errors of the multilevel methods as a function of the active subsystems for test reaction III. For a detailed explanation of the axes, please refer to Fig. S13.

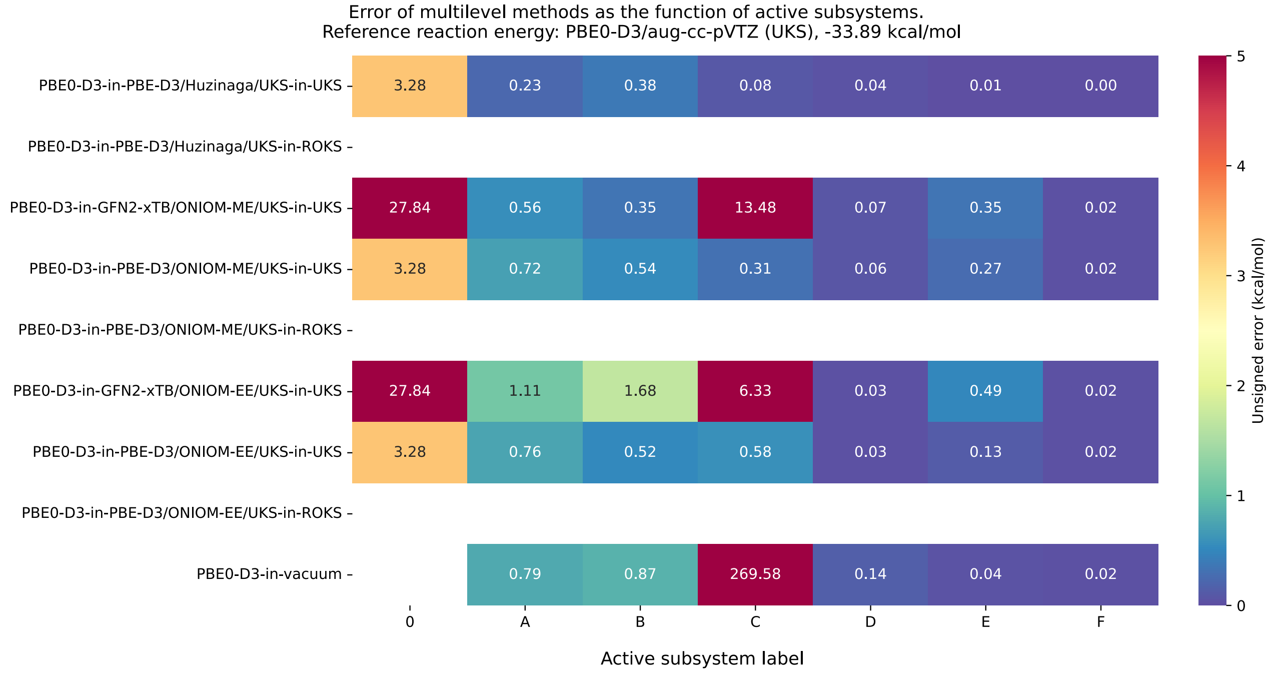

Figure S17: Unsigned errors of the multilevel methods as a function of the active subsystems for test reaction III. For a detailed explanation of the axes, please refer to Fig. S13.

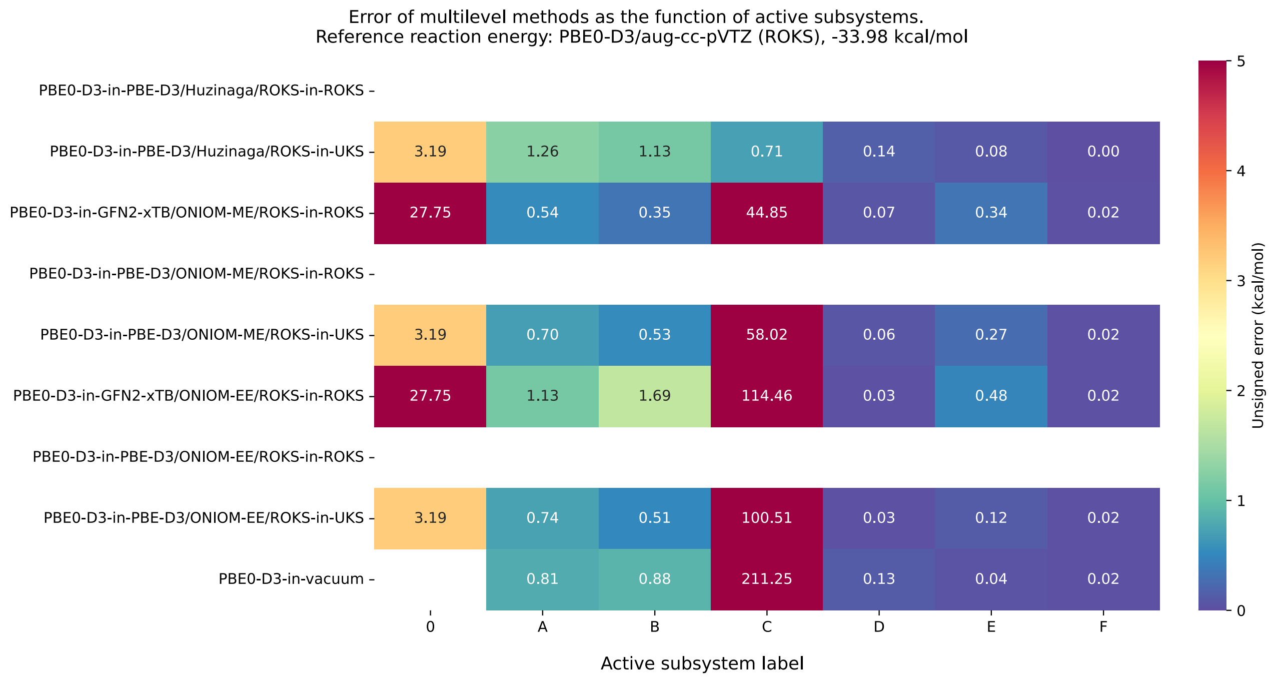

Figure S18: Unsigned errors of the multilevel methods as a function of the active subsystems for test reaction III. For a detailed explanation of the axes, please refer to Fig. S13.

4 Results for test reaction IV

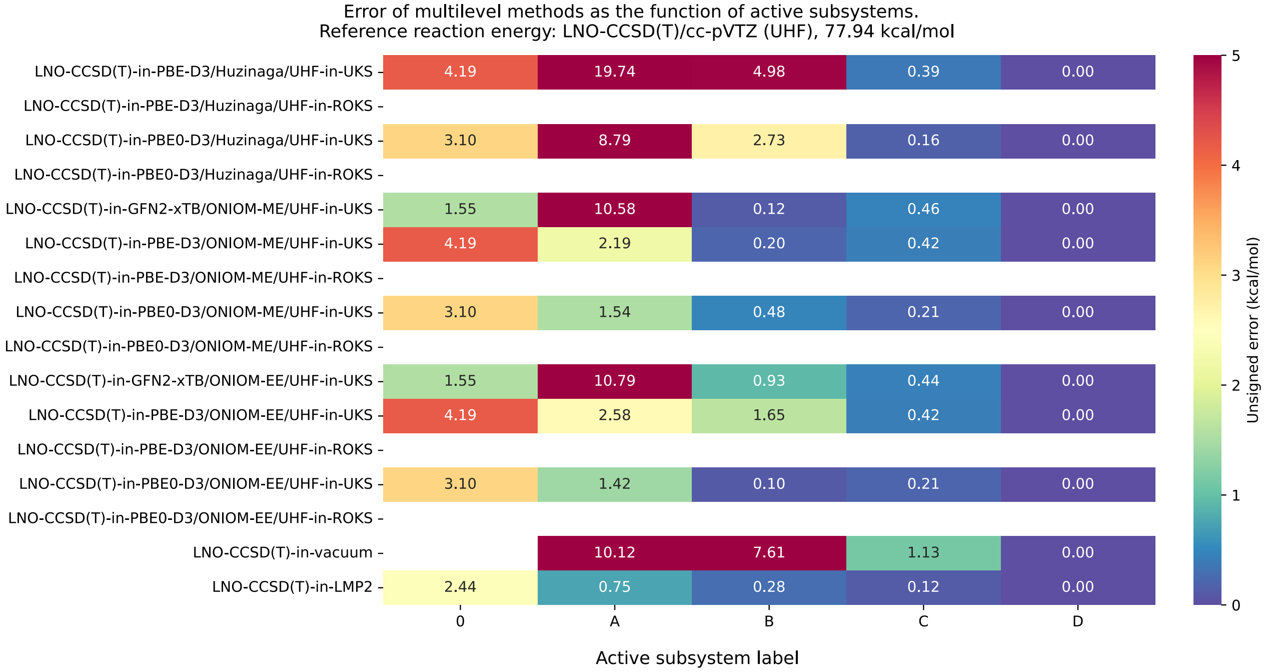

Figure S19: Unsigned errors of the multilevel methods as a function of the active subsystems for test reaction IV. The meaning of the active subsystem labels is shown in Fig. 10, moreover, label "0" denotes results of the low-level methods. The leftmost column shows the type of the wave function Ansatz used for the open-shell molecules and the theoretical method applied at the low-level. Note that empty rows indicate low-level methods that did not yield SCF solutions for the triplet state.

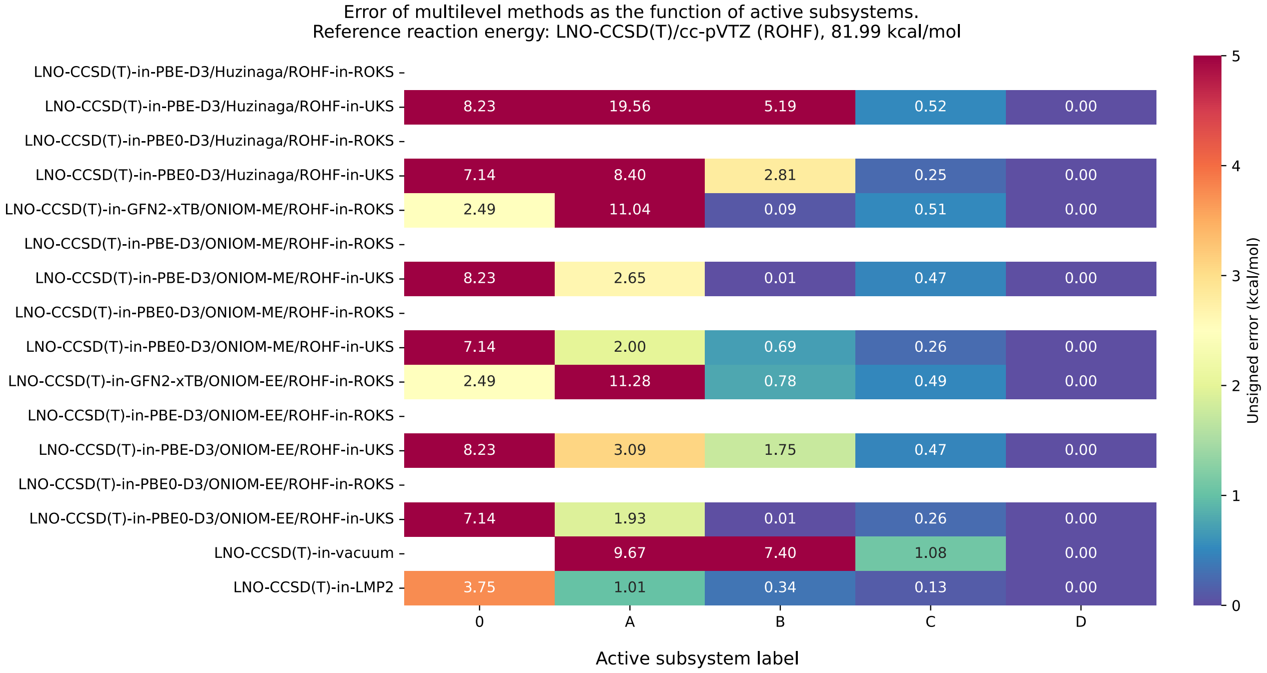

Figure S20: Unsigned errors of the multilevel methods as a function of the active subsystems for test reaction IV. For a detailed explanation of the axes, please refer to Fig. S19.

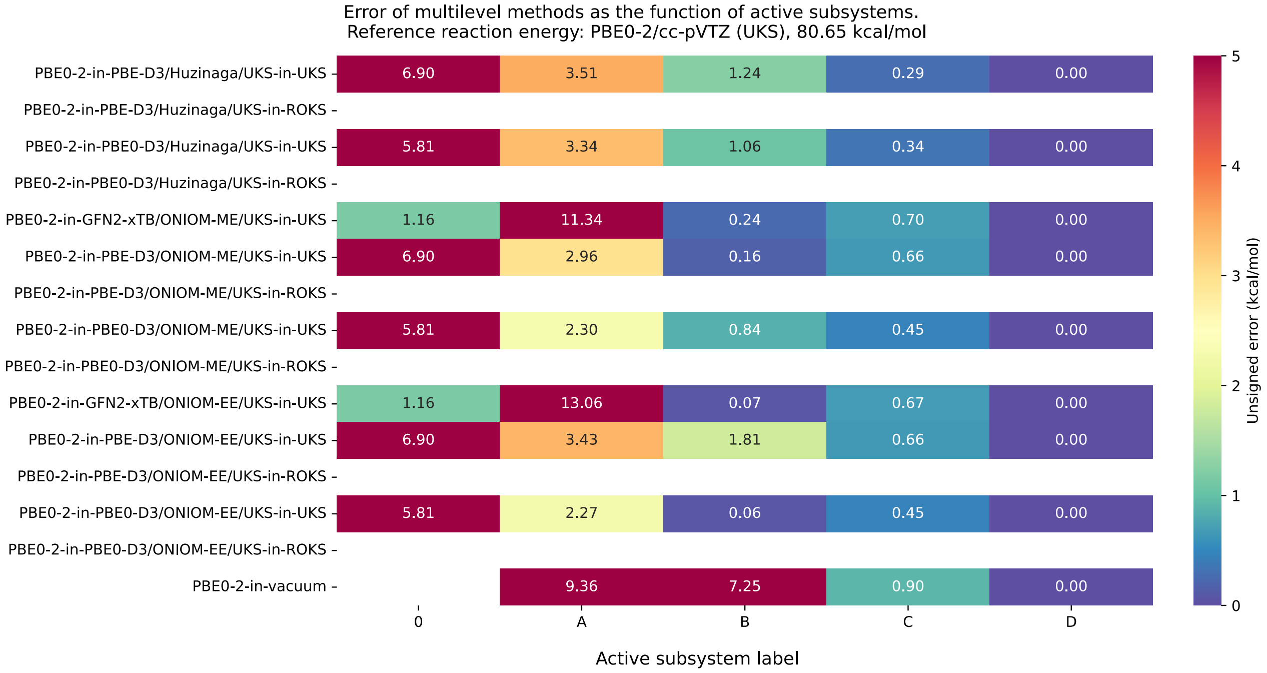

Figure S21: Unsigned errors of the multilevel methods as a function of the active subsystems for test reaction IV. For a detailed explanation of the axes, please refer to Fig. S19.

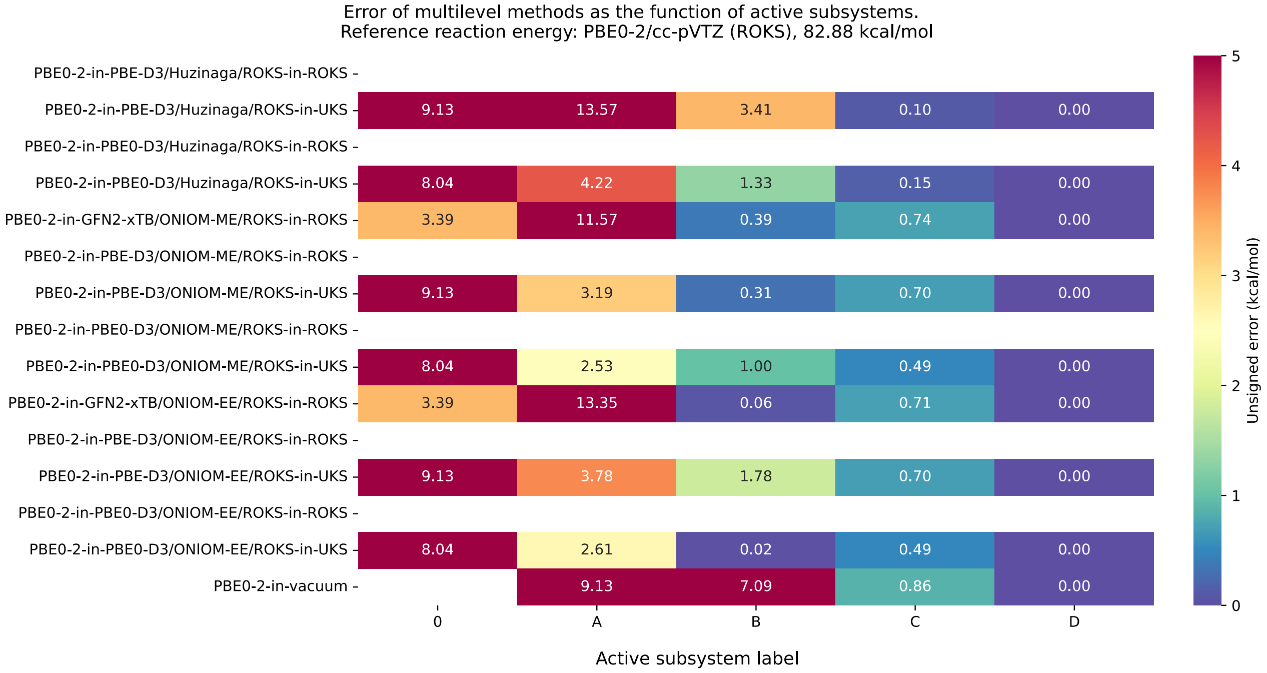

Figure S22: Unsigned errors of the multilevel methods as a function of the active subsystems for test reaction IV. For a detailed explanation of the axes, please refer to Fig. S19.

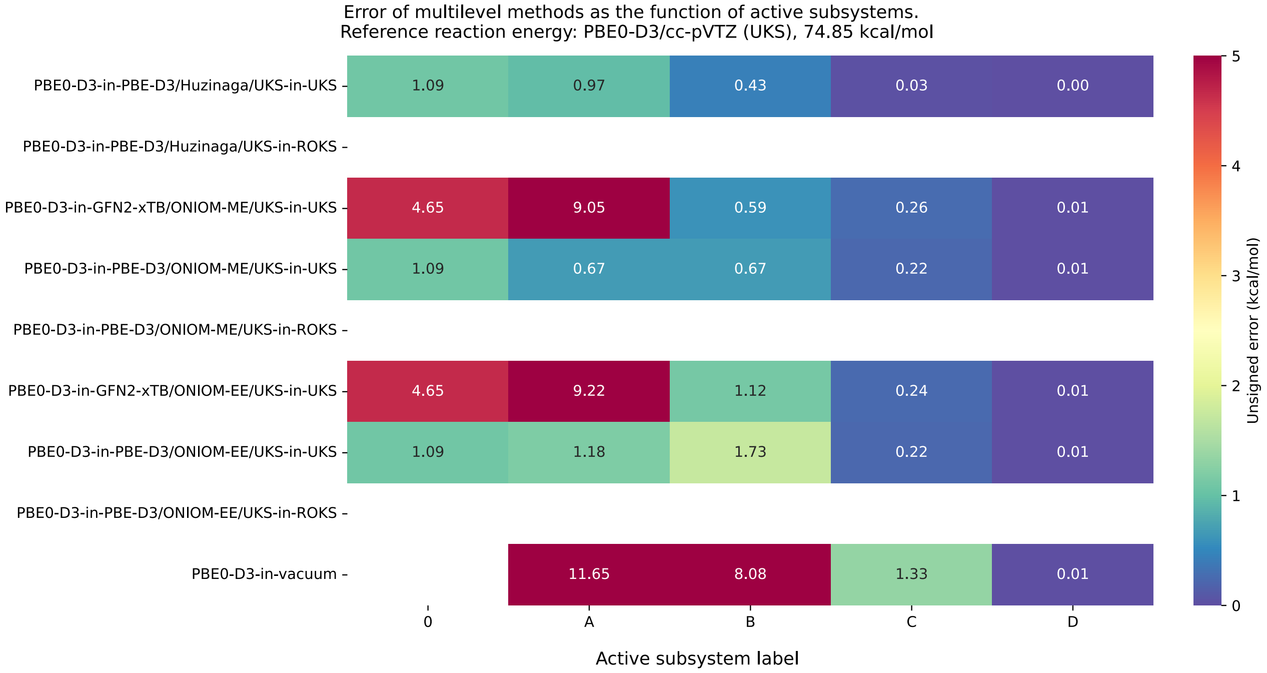

Figure S23: Unsigned errors of the multilevel methods as a function of the active subsystems for test reaction IV. For a detailed explanation of the axes, please refer to Fig. S19.
